# Erratum to “NUFIP1-Mediated Ribophagy Alleviates PANoptosis of CD4^+^ T Lymphocytes in Sepsis via the cGAS-STING Pathway”

**DOI:** 10.34133/research.1173

**Published:** 2026-03-03

**Authors:** Pengyue Zhao, Jingyan Li, Pengyi He, Yao Wu, Liyu Zheng, Xingpeng Yang, Jiaqi Yang, Ze Fu, Yun Xia, Ning Chen, Ning Dong, Zhiwen Luo, Renqi Yao, Xiaohui Du, Yongming Yao

**Affiliations:** ^1^ Medical Innovation Research Division and Fourth Medical Center of the Chinese PLA General Hospital, Beijing 100853, China.; ^2^ Department of General Surgery, First Medical Center of the Chinese PLA General Hospital, Beijing 100853, China.; ^3^ Department of Emergency, Second Hospital of Hebei Medical University, Shijiazhuang 050000, China.; ^4^ Department of Anesthesiology, Zhongnan Hospital of Wuhan University, Wuhan 430000, China.; ^5^ Department of Sports Medicine, Huashan Hospital, Fudan University, Shanghai 200040, China.; ^6^ National Clinical Research Center for Geriatric Diseases, the Chinese PLA General Hospital, Beijing 100853, China.

The authors have identified several errors in the Research Article entitled “NUFIP1-mediated Ribophagy Alleviates PANoptosis of CD4^+^ T Lymphocytes in Sepsis via the cGAS-STING Pathway” [1].

Specifically, the Western blot bands of β-actin and Bax in the Fig. 4E panel and the SYTOX green images for the CLP group (Fig. S3A) were incorrectly presented. After carefully re-examining the original data, we determined that these discrepancies resulted from operator error during the figure assembly process in Adobe Illustrator.

**Fig. 4.**
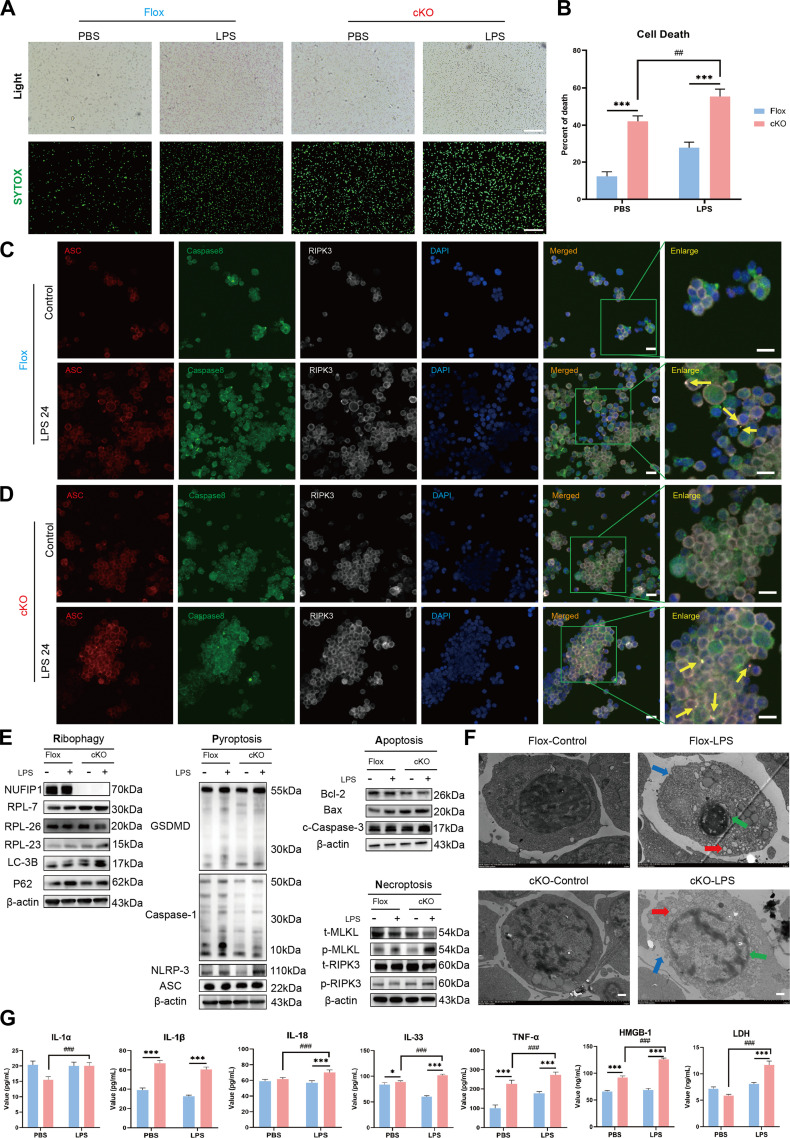
Impact of conditional deletion of *NUFIP1* on PANoptosis of splenic CD4^+^ T lymphocytes in septic mice. (A and B) The necrosis of splenic CD4^+^ T lymphocytes in mice stimulated with LPS was detected by SYTOX-Green. The scale bar represents 100 μm. *n* = 3 technical repetitions. (C) The expression and co-localization of ASC/caspase-8/RIPK3 in splenic CD4^+^ T lymphocytes of Flox mice stimulated with LPS were detected by LSCM. The yellow arrows represent PANoptosomes, and the scale bar represents 25 μm. (D) The expression and co-localization of ASC/caspase-8/RIPK3 in splenic CD4^+^ T lymphocytes of cKO mice stimulated with LPS were detected by LSCM. The yellow arrows represent PANoptosomes, and the scale bar represents 25 μm. (E) Expression of PANoptosis-related proteins in splenic CD4^+^ T lymphocytes of cKO mice stimulated with LPS under WB. (F) Morphological characteristics of PANoptosis in splenic CD4^+^ T lymphocytes of Flox and cKO mice stimulated with LPS under TEM. The red arrow denotes the intracellular vesicles associated with pyroptosis; the green arrow signifies the nuclear condensation and chromatin shrinkage characteristic of cell apoptosis; the blue arrow indicates the loss of cell membrane integrity in necroptosis, and the scale bar represents 500 nm. (G) Cytokine levels in the culture supernatant of splenic CD4^+^ T cells were measured by ELISA in Flox and cKO groups. *n* = 6 technical repetitions. Data were expressed as means ± SEM. A 2-way ANOVA test was applied to test the statistical significance. *P < 0.05, ***P < 0.001 compared with the Flox group. ^##^*P* < 0.01, ^###^*P* < 0.001 compared with the cKO-PBS group.

In Fig. 4F, the TEM images for the Flox groups were inadvertently duplicated from the Control group shown in Fig. 2I. This occurred during the selection of images from different folders while preparing the figures. A similar duplication error is present in the HE staining images of Fig. 8C.

**Fig. 8.**
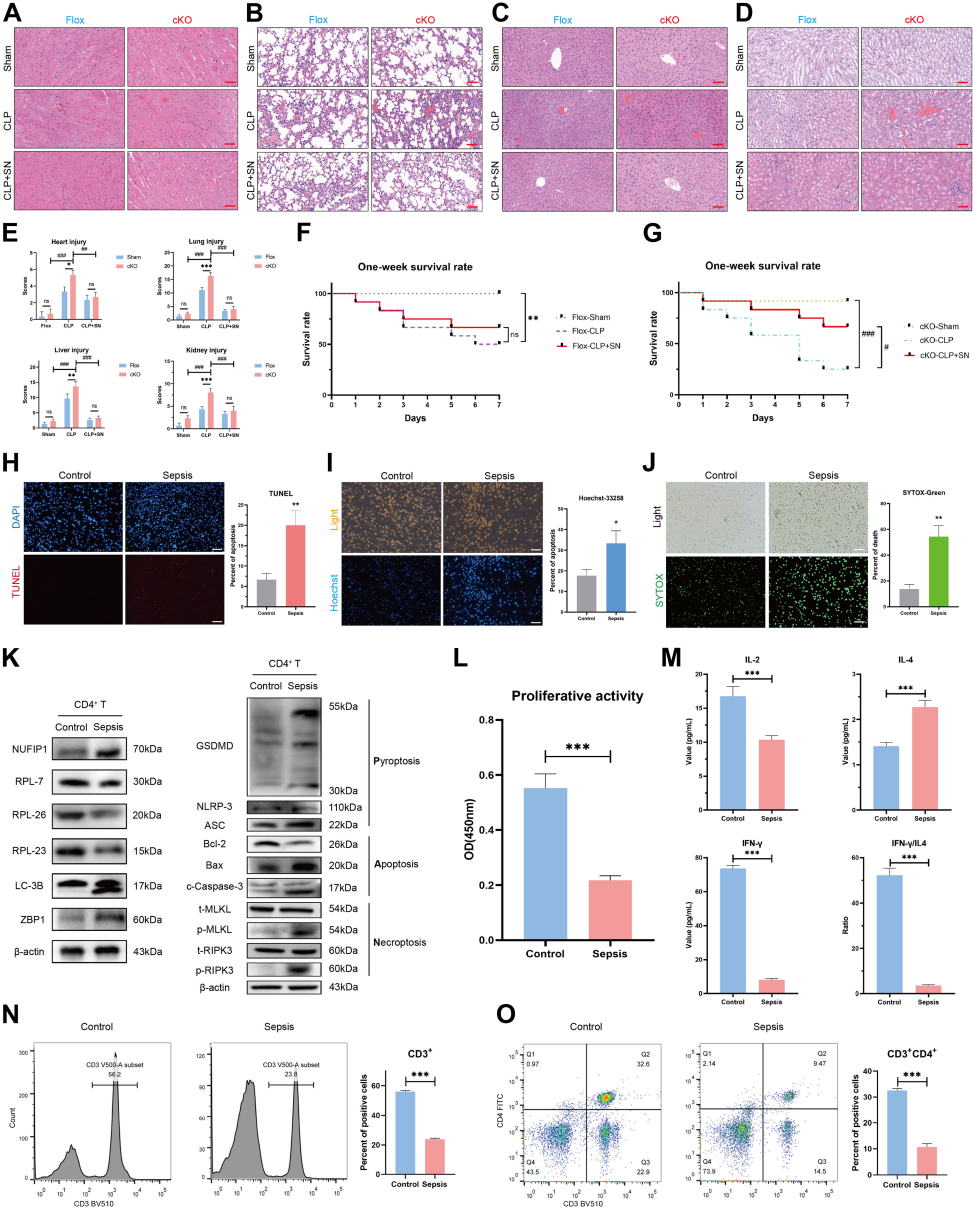
Regulatory effects of cGAS-STING signaling on peripheral immune status in septic mice and ribophagy, PANoptosis, and immune function of peripheral blood CD4^+^ T lymphocytes in sepsis patients. (A to E) H&E staining assessed the effects of SN-011 on multiple organ damage, including, heart (A), lung (B), liver (C), and kidney (D) in different groups of mice. The scale bar represents 50 μm. *n* = 3 biological independent samples. (Two-way ANOVA was applied to test the statistical significance. ^##^*P* < 0.01, ^###^*P* < 0.001 compared with the cKO-CLP groups). (F and G) The impact of SN-011 on the one-week survival rate of Flox (F) and cKO (G) mice. *n* = 10 biological independent samples. (** *P*<0.01 compared with the sham group; ^#^*P*<0.05, ^###^*P* < 0.001 compared with the cKO-CLP group). (H) The apoptosis of CD4^+^ T lymphocytes in the peripheral blood of patients with and without sepsis was detected by TUNEL. The scale bar represents 100 μm. *n* = 3 technical repetitions. (I) The apoptosis of CD4^+^ T lymphocytes in the peripheral blood of patients with and without sepsis was determined by Hoechst 33258. The scale bar represents 100 μm. *n* = 3 technical repetitions. (J) Necrosis of CD4^+^ T lymphocytes in the peripheral blood of patients with and without sepsis was detected by SYTOX-Green. The scale bar represents 100 μm. *n* = 3 technical repetitions. (K) The expression of ribophagy and PANoptosis-related proteins in the peripheral blood CD4^+^ T lymphocytes of patients with and without sepsis was analyzed by WB. (L) The proliferative activity of CD4^+^ T lymphocytes in the peripheral blood of patients with and without sepsis was detected by CCK-8. *n* = 3 technical repetitions. (M) Serum cytokine levels in patients with and without sepsis were measured by ELISA. *n* = 5 technical repetitions. (N and O) The proportions of CD3^+^ T (N) and CD3^+^CD4^+^ T (O) lymphocytes in the peripheral blood of patients with and without sepsis were analyzed by flow cytometry. *n* = 3 technical repetitions. Data were expressed as means ± SEM. An unpaired 2-sided Student’s t test was applied to test the statistical significance. *P < 0.05, **P < 0.01, ***P < 0.001.

To facilitate verification, all original data corresponding to the affected figures have been provided for review. The authors would like to emphasize that these inadvertent errors do not affect the data analysis, results, or overall conclusions of the study. Corrected versions of the Figures 4 and 8 are below, and the online HTML and PDF versions of the article have been updated accordingly. Additionally, the Supplementary Materials package has been updated to reflect the revised Figure S3.
