# Supplementary material for: NUFIP1-Mediated Ribophagy Alleviates PANoptosis of CD4+ T Lymphocytes in Sepsis via the cGAS-STING Pathway
Source: Research (Wash D C). 2025 Sep 23;8:0895. doi: 10.34133/research.0895 (PMC12454940; doi:10.34133/research.0895)
Supplement: 20250923-1 [file research.0895.v1.pdf]

Research
A SCIENCE PARTNER JOURNAL

RESEARCH ARTICLE

# NUFIP1-Mediated Ribophagy Alleviates PANoptosis of CD4$^+$ T Lymphocytes in Sepsis via the cGAS-STING Pathway

**Pengyue Zhao[1,2†], Jingyan Li[1,3†], Pengyi He[1,2†], Yao Wu[1], Liyu Zheng[1], Xingpeng Yang[1,2], Jiaqi Yang[1,2], Ze Fu[1,2], Yun Xia[4], Ning Chen[1], Ning Dong[1], Zhiwen Luo[5*], Renqi Yao[1,2*], Xiaohui Du[2*], and Yongming Yao[1,6*]**

[1]Medical Innovation Research Division and Fourth Medical Center of the Chinese PLA General Hospital, Beijing 100853, China. [2]Department of General Surgery, First Medical Center of the Chinese PLA General Hospital, Beijing 100853, China. [3]Department of Emergency, Second Hospital of Hebei Medical University, Shijiazhuang 050000, China. [4]Department of Anesthesiology, Zhongnan Hospital of Wuhan University, Wuhan 430000, China. [5]Department of Sports Medicine, Huashan Hospital, Fudan University, Shanghai 200040, China. [6]National Clinical Research Center for Geriatric Diseases, the Chinese PLA General Hospital, Beijing 100853, China.

*Address correspondence to: c_ff@sina.com (Y.Y.); duxiaohui301pla@sina.com (X.D.); yaorenqixx1995@163.com (R.Y.); zhiwen.luo_fudan@hotmail.com (Z.L.)
†These authors contributed equally to this work.

**Citation:** Zhao P, Li J, He P, Wu Y, Zheng L, Yang X, Yang J, Fu Z, Xia Y, Chen N, et al. NUFIP1-Mediated Ribophagy Alleviates PANoptosis of CD4$^+$ T Lymphocytes in Sepsis via the cGAS-STING Pathway. *Research* 2025;8:Article 0895. https://doi.org/10.34133/research.0895

Submitted 19 June 2025
Revised 24 August 2025
Accepted 25 August 2025
Published 23 September 2025

Copyright © 2025 Pengyue Zhao et al. Exclusive licensee Science and Technology Review Publishing House. No claim to original U.S. Government Works. Distributed under a Creative Commons Attribution License (CC BY 4.0).

T lymphocyte dysfunction represents a pivotal determinant of immunosuppression in sepsis. Our previous studies demonstrated that nuclear fragile X mental retardation-interacting protein 1 (NUFIP1)-mediated ribophagy conferred cytoprotection against apoptosis in CD4$^+$ T lymphocytes during sepsis, thereby preserving host immunocompetence. Despite growing evidence linking PANoptosis to the pathogenesis of various diseases, the potential role of ribophagy in modulating CD4$^+$ T lymphocytes' PANoptosis in sepsis remains largely unclear. In the present study, we employed both lipopolysaccharide-stimulated Jurkat T cells and cecal ligation and puncture (CLP)-induced sepsis models to demonstrate marked exacerbation of CD4$^+$ T lymphocyte PANoptosis following *NUFIP1* knockdown (KD), associated with impaired immune function, as evidenced by diminished cytokine production and T cell proliferation. Tandem mass tagging (TMT) proteomic analysis identified Z-nucleic acid binding protein 1 (ZBP1)-mediated PANoptosome formation and the cyclic GMP-AMP synthase–stimulator of interferon genes (cGAS-STING) pathway as critical nodes in ribophagy-dependent cytoprotection. Mechanistically, sepsis-induced ribosome collision activated the cGAS-STING signaling axis, which in turn recruited NUFIP1 to STING protein complexes. Clinical analysis of septic patients revealed enhanced ribophagy and PANoptosis in peripheral blood CD4$^+$ T cells, consistent with the experimental findings. These results suggest that NUFIP1-mediated ribophagy alleviates CD4$^+$ T lymphocyte PANoptosis in sepsis via the cGAS-STING pathway, highlighting the therapeutic potential of targeting ribophagy and PANoptosis pathways to mitigate immune paralysis and improve the outcomes following septic insults.

## Introduction

Sepsis, a life-threatening pathological condition characterized by multi-organ system failure resulting from a dysregulated host response to infection, constitutes a formidable global health challenge [1]. Epidemiological data indicate a staggering burden of 48.9 million incident cases and 11 million attributable deaths worldwide [2]. Consequently, unraveling the intricate pathophysiological mechanisms underlying sepsis and optimizing therapeutic strategies represent paramount challenges in critical care medicine, necessitating a paradigm shift toward precision-based interventions.

Sepsis-induced immunosuppression has been demonstrated to be a pivotal determinant of late-stage morbidity and mortality [3,4]. As central orchestrators of adaptive immunity, T lymphocytes maintain immune homeostasis through the secretion of cytokines, antigen presentation, and the regulation of effector cell function. Accumulating evidence from both clinical and preclinical studies suggests a causal link between T cell dysfunction and immune dysregulation in sepsis characterized by

reduced CD4$^+$ T cell counts and proliferative activities [5,6]. Our previous study revealed that excessive apoptosis of CD4$^+$ T cells compromised their numerical and functional integrity, manifesting as reduced proliferative capacity, impaired effector function, and a shift toward helper T cell 2 (Th2) polarization, thereby exacerbating sepsis-induced immune depression [7]. Thus, therapeutic interventions aimed at restoring T cell homeostasis and function represent a critical frontier in the management of sepsis-associated immunosuppression.

Programmed cell death (PCD), a fundamental regulatory mechanism governing organismal development and homeostatic equilibrium, serves as an indispensable defense against pathogen elimination and a critical component of immunological surveillance [8]. Increasing evidence suggests that molecular effectors of distinct PCD modalities form an interconnected network, wherein a single pathogenic or sterile insult can simultaneously activate multiple PCD cascades through cross-talk pathways [9]. Our recent studies have elucidated a novel paradigm in the pathophysiology of sepsis, demonstrating co-activation of divergent PCD programs in immune cell subsets. Notably, CD4$^+$ T lymphocytes undergo apoptosis, pyroptosis, and cuproptosis, while dendritic cells display pyroptosis, necroptosis, and ferroptosis under septic conditions [10–13]. The dysregulation of these multifaceted PCD pathways has been correlated with immunopathological sequelae observed in septic patients, underscoring the therapeutic potential of precision-targeted modulation of multiple PCD pathways to restore immune homeostasis in sepsis. However, the limitations of single PCD modulation in immune cells to fully account for sepsis-induced immune dysfunction and multi-organ failure underscore the necessity for paradigm-shifting advancements in diagnostic biomarkers and therapeutic strategy [14]. PANoptosis, characterized by synchronized execution of pyroptosis, apoptosis, and necroptosis through shared morphological and molecular signatures, has revolutionized cell death research by providing a unified framework for understanding coordinated cell demise programs [15]. Systematic exploration of PANoptosis across disease spectra has revealed its pathogenic involvement in a variety of human diseases [16–20]. Notably, the functional role of PANoptosis in immune cell populations remains underexplored, particularly regarding the characterization of CD4$^+$ T lymphocytes' PANoptosis kinetics and regulatory networks during sepsis progression. Given that PANoptosis-induced immunopathology directly contributes to T cell exhaustion and cytokine storm in septic patients, targeted inhibition of PANoptosis signaling may emerge as a novel therapeutic avenue to reconstitute T cell immunity and prevent sepsis-induced immunosuppression.

Autophagy functions as a critical sentinel mechanism preserving cellular homeostasis by degrading misfolded proteins and recycling damaged organelles [21]. Preclinical studies demonstrated compensatory up-regulation of organelle-specific autophagy in sepsis, correlating with improved multi-organ function and survival outcomes [22]. However, while these findings underscore the cytoprotective role of autophagy networks, the functional hierarchy and spatiotemporal regulation of ribophagy in sepsis-associated immunometabolic reprogramming remain enigmatic [23]. This knowledge gap underscores the need for further investigation into the potential role of ribophagy as both a quality control pathway and an immunomodulatory target during septic progression.

Ribosomes are indispensable for maintaining translational fidelity, and translation arrest or ribosome failure-induced ribosome collision leads to incomplete synthesized polypeptide chains and ribosomal RNA (rRNA) leakage into the cytoplasm. The leaked rRNA is recognized by cyclic guanosine 3′,5′-monophosphate (GMP)-adenosine 5′-monophosphate (AMP) synthase (cGAS) as a danger signal, and its negatively charged phosphate backbone directly binds to the positively charged DNA-binding domain of cGAS. This binding triggers conformational changes in cGAS, catalyzes the synthesis of the second messenger 2′,3′-cyclic guanosine monophosphate-adenosine monophosphate (cGAMP), and subsequently activates the stimulator of interferon genes (STING)–TANK-binding kinase 1 (TBK1)–interferon regulatory factor 3 (IRF3) pathway, driving the expression of inflammatory factors [24]. To mitigate such threats, eukaryotic cells have evolved a hierarchical ribosome quality control system (RQCS) that orchestrates ribosomal surveillance across their lifecycle from coordinated biogenesis to regulated turnover [25]. Within this system, ribophagy has emerged as a pivotal selective autophagy dedicated to ribosomal clearance. Initially characterized in *Saccharomyces cerevisiae* through seminal work by Kraft et al., the mechanistic underpinnings of mammalian ribophagy were elucidated with the identification of nuclear fragile X mental retardation-interacting protein 1 (NUFIP1) as its cognate receptor, establishing molecular targets for pathway modulation [26,27]. Our previous study demonstrated that NUFIP1-dependent ribophagy exerted potent cytoprotective effects on the immune response of CD4$^+$ T lymphocytes secondary to septic challenge [11]. Integrating the recently proposed concept of PANoptosis and the concurrent existence of multiple interconnected PCD modes in immune cells, we hypothesize that NUFIP1-mediated ribophagy might play a regulatory role in PANoptosis of CD4$^+$ T lymphocytes during sepsis.

To validate the hypothesis above, both the cecal ligation and puncture (CLP) model in vivo and lipopolysaccharide (LPS)-treated cells in vitro were employed to simulate septic conditions. Lentiviral transfection and the generation of $Cd4^{cre}Nufip1^{fl/fl}$ animals were utilized to manipulate *NUFIP1*, investigating its impact on CD4$^+$ T lymphocyte PANoptosis, immune activity, multi-organ damage, and survival rate. Subsequently, proteomic sequencing was conducted to identify key molecules and potential signaling pathways. Polysome profiling was performed to detect ribosome collisions, and co-immunoprecipitation (Co-IP) was used to explore the interaction between NUFIP1 and key molecules. Small-molecule inhibitor rescue experiments were carried out to further elucidate the precise molecular mechanisms. In addition, CD4$^+$ T lymphocytes were isolated from peripheral blood samples in sepsis and nonsepsis patients, validating the significance of ribophagy and PANoptosis in clinical cases.

## Results

### PANoptosis of CD4$^+$ T lymphocytes in sepsis

We found that prolonged LPS stimulation and CLP-induced sepsis resulted in a transient increase followed by a decrease in the apoptotic ratio, SYTOX-Green necrosis ratio of CD4$^+$ T lymphocytes, and levels of key PANoptosis-related proteins, including gasdermin D (GSDMD), nucleotide binding oligomerization domain-like receptor protein 3 (NLRP3), apoptosis speck-like protein containing a caspase recruitment domain

(ASC), BCL2-associated X protein (Bax), cleaved caspase-3 (c-caspase-3), phosphorylated mixed family kinase-like domain (p-MLKL), and phosphorylated receptor-interacting protein kinase 3 (p-RIPK3). A statistically significant peak was observed at 24 h (Fig. 1A to C and Fig. S1A to F). The dose–response experiment revealed that the expression of PANoptosis-related proteins exhibited the most significant elevation upon stimulation with 500 ng/ml LPS (Fig. S1G and H).

To verify $CD4^+$ T lymphocyte PANoptosis in sepsis, based on time– and dose–response experiments, laser confocal microscopy was used to detect the expression and colocalization of key PANoptosis proteins in $CD4^+$ T lymphocytes. Our findings revealed a notable increase in the expression of ASC/caspase-8/RIPK3, key PANoptosis proteins, within T cells during sepsis. Moreover, the colocalization of these proteins was more pronounced, with distinct PANoptosomes indicated by a yellow arrow (Fig. 1D to F). The Western blot (WB) results confirmed a significant elevation in the expression of $CD4^+$ T lymphocyte PANoptosis-related protein in the sepsis group compared to the control group (Fig. 1G). Enzyme-linked immunosorbent assay (ELISA) results showed a significant increase in secretion of pyroptosis-related cytokines, including interleukin-1β (IL-1β), IL-18, IL-1α, IL-33, tumor necrosis factor-α (TNF-α), and high mobility group box one protein (HMGB1), along with necrosis-related cytokine lactate dehydrogenase (LDH) in both $CD4^+$ T lymphocyte culture supernatant and serum of mice in the sepsis group compared to the control group (Fig. 1H and I). Transmission electron microscopy (TEM) illustrated that $CD4^+$ T lymphocytes from the sepsis group displayed distinct morphological features indicative of pyroptosis, apoptosis, and necrotic apoptosis. Specifically, cells undergoing pyroptosis exhibited intracellular vesicles and pyroptosomes in the cytoplasm, along with the formation of pores on the cell membrane. Apoptotic cells showed nucleus shrinkage, chromatin condensation, and apoptosomes in the cytoplasm while maintaining an intact cell membrane. Cells undergoing necroptosis displayed a combination of characteristics from both apoptosis and necrosis, primarily characterized by the loss of membrane integrity and nuclear dissolution (Fig. 1J to L).

## Sepsis induces activation of ribophagy in $CD4^+$ T lymphocytes

The impact of NUFIP1-mediated ribophagy on $CD4^+$ T lymphocyte PANoptosis in sepsis was investigated by establishing a lentiviral transfection model in Jurkat T cells. Results depicted in Fig. 2A demonstrated successful transfection of Jurkat T cells with the lentivirus carrying *NUFIP1* gene KD and overexpression, as evidenced by high transfection efficiency. A significant decrease in NUFIP1 protein expression was observed in the KD group and a marked increase in the overexpression group compared to the control group (Fig. 2B). As shown in Fig. 2C, the expression and colocalization of the ribophagy receptor NUFIP1, lysosome-specific marker protein lysosomal-associated membrane protein 2 (LAMP-2), and autophagy protein light chain 3B (LC-3B) in Jurkat T cells were significantly enhanced at 24 h after LPS stimulation. Similar observations were made in splenic $CD4^+$ T lymphocytes subjected to LPS stimulation in vitro and CLP in vivo (Fig. 2D and E). Significant expression of the ribophagy-specific receptor NUFIP1 and autophagy-associated protein LC-3B was observed in $CD4^+$ T lymphocytes after sepsis, with a corresponding decrease in the expression of ribosomal self-proteins ribosomal protein L7 (RPL7), RPL23, and RPL26 (Fig. 2F to H). TEM confirmed the presence of typical bilayer autophagosomes containing degraded ribosomes in $CD4^+$ T lymphocytes from the sepsis group, providing further evidence of ribophagy activation (Fig. 2I).

## NUFIP1-mediated ribophagy alleviates $CD4^+$ T lymphocyte ZBP1-PANoptosis in sepsis

Confocal laser microscopy revealed a significant enhancement in the expression and colocalization of PANoptotic proteins ASC/caspase-8/RIPK3 following *NUFIP1* KD (Fig. 3A and B). Furthermore, WB results showed activation of PANoptosis-related proteins in Jurkat T cells upon *NUFIP1* KD, contrasting with reduced levels upon *NUFIP1* overexpression (Fig. 3C). TEM observations indicated evident PANoptotic morphological alterations in *NUFIP1*-KD Jurkat T cells (Fig. 3D). Recent studies have implicated Z-nucleic acid binding protein 1 (ZBP1), absent in melanoma 2 (AIM2), RIPK1, and NLRP12 as crucial proteins involved in the development of PANoptosomes [16,28]. Quantification of these proteins in Jurkat T cells showed that LPS increased NUFIP1, NLRP12, and ZBP1 protein levels to some extent. Silencing the *NUFIP1* gene in Jurkat T cells led to a significant decrease in NUFIP1 expression and an increase in NLRP12 and ZBP1 levels, while RIPK1 and AIM2 expressions remained relatively constant (Fig. 3E and F). We next generated a conditional knockout (cKO) mouse model for the *NUFIP1* gene ($Cd4^{cre}Nufip1^{fl/fl}$) and its corresponding control mouse ($Nufip1^{fl/fl}$). $CD4^+$ T lymphocytes were isolated from the spleens and cultured in vitro. WB was used to assess the levels of these crucial proteins responsible for the generation of PANoptosomes. Up-regulated expression of NUFIP1 and ZBP1 was observed after treatment with LPS in vitro and CLP in vivo, with statistically significant differences. Upon cKO of *NUFIP1* in $CD4^+$ T lymphocytes of mice, the expression of ZBP1 showed further enhancement. However, the expression patterns of NLRP12, AIM2, and RIPK3, the other 3 pivotal molecules associated with PANoptosomes, did not align with the expected outcomes (Fig. 3G to J). To further elucidate the role of ZBP1-mediated PANoptosomes in alleviating $CD4^+$ T cells PANoptosis via ribophagy, we performed a Co-IP assay to assess the interaction between NUFIP1 and ZBP1. The results demonstrated a significant interaction between NUFIP1 and ZBP1 in both Jurkat T cells and primary $CD4^+$ T lymphocytes isolated from mouse spleen (Fig. S2). Moreover, to elucidate the impact of NUFIP1-mediated ribophagy on the PANoptotic response of $CD4^+$ T lymphocytes, we isolated splenic $CD4^+$ T lymphocytes of mice subjected to LPS stimulation and the CLP model. The results revealed a significant increase in $CD4^+$ T lymphocyte PANoptosis in cells with *NUFIP1* deficiency (Fig. 4). Similar findings were observed in the CLP-induced sepsis model (Fig. S3).

## Impact of conditional deletion of *NUFIP1* on immune response of $CD4^+$ T lymphocytes, organ injury, and the 1-week survival rate of mice in sepsis

Next, $Cd4^{cre}Nufip1^{fl/fl}$ mice were utilized to explore the influence of conditional *NUFIP1* knockout on $CD4^+$ T cell immune function, multi-organ damage, and prognosis in sepsis. Proliferative activity of $CD4^+$ T lymphocytes in sepsis was assessed using

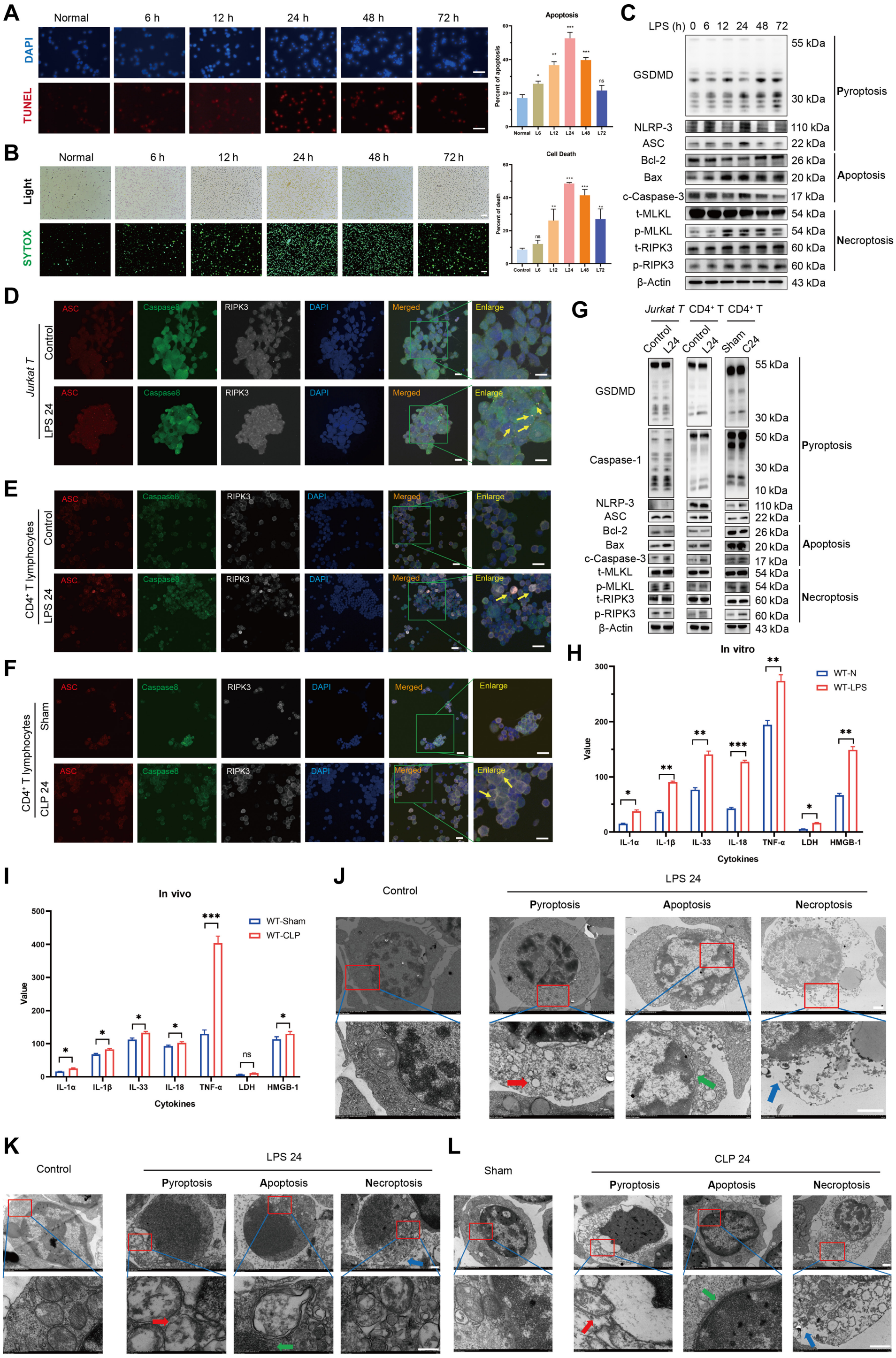


**Fig. 1.** PANoptosis of CD4$^+$ T lymphocytes in sepsis. (A) Apoptosis of Jurkat T cells was detected by TUNEL after LPS stimulation at different time points. The scale bar represents 100 μm. $n = 3$ technical repetitions. (B) Necrosis of Jurkat T cells was detected by SYTOX-Green after LPS stimulation at different time points. The scale bar represents 100 μm. $n = 3$ technical repetitions. (C) WB determined the expression of PANoptosis-related proteins in Jurkat T cells after LPS stimulation at different time points. (D) The expression and colocalization of PANoptosis core protein ASC/caspase-8/RIPK3 in Jurkat T cells stimulated with LPS were determined by LSCM. The yellow arrows represent PANoptosomes, and the scale bar represents 25 μm. (E) The expression and colocalization of ASC/caspase-8/RIPK3 in splenic CD4$^+$ T cells stimulated with LPS were determined by LSCM. (F) Expression and colocalization of ASC/caspase-8/RIPK3 in splenic CD4$^+$ T cells by LSCM after CLP operation. (G) WB detected the expression of CD4$^+$ T lymphocyte PANoptosis-related protein in sepsis. (H) Cytokine secretion levels of splenic CD4$^+$ T cells in culture supernatant were measured by ELISA. $n = 6$ technical repetitions. (I) Serum cytokine levels of mice in different groups were measured by ELISA. $n = 6$ technical repetitions. (J) The morphological characteristics of PANoptosis in Jurkat T cells were observed by TEM. The red arrow denotes the intracellular vesicles associated with pyroptosis; the green arrow signifies the nuclear condensation and chromatin shrinkage characteristic of cell apoptosis; the blue arrow indicates the loss of cell membrane integrity in necroptosis; and the scale bars represent 500 nm (top) and 100 nm (bottom). (K) The morphological characteristics of PANoptosis in splenic CD4$^+$ T lymphocytes in vitro were observed by TEM. (L) The morphological characteristics of PANoptosis in splenic CD4$^+$ T lymphocytes in vivo were observed by TEM. Data were expressed as means ± SEM. An unpaired 2-sided Student's $t$ test was applied to test the statistical significance. *$P < 0.05$, **$P < 0.01$, ***$P < 0.001$.

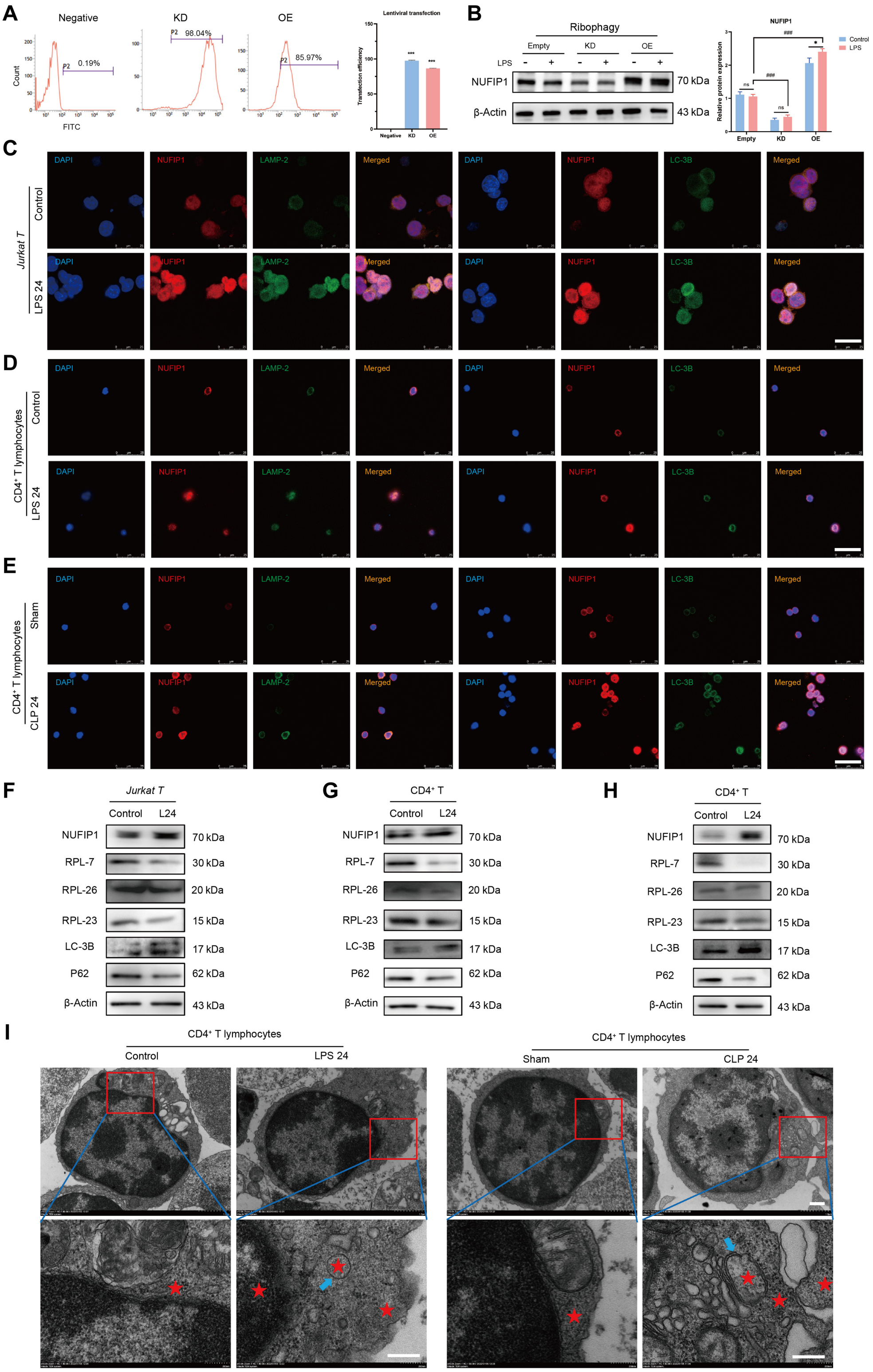


**Fig. 2.** Sepsis induces activation of ribophagy in CD4$^+$ T lymphocytes. (A) The transfection efficiency of lentivirus in different groups was measured by flow cytometry. $n = 3$ technical repetitions. (B) The expression of NUFIP1 protein in different groups of Jurkat T cells was detected by WB. $n = 3$ technical repetitions. (C) The expression and colocalization of NUFIP1, LAMP2, and LC3B in Jurkat T cells stimulated with LPS were detected by LSCM. The scale bar represents 25 μm. (D) The expression and colocalization of NUFIP1, LAMP2, and LC3B in splenic CD4$^+$ T cells stimulated with LPS were detected by LSCM. The scale bar represents 25 μm. (E) The expression and colocalization of NUFIP1, LAMP2, and LC3B in splenic CD4$^+$ T cells by LSCM after CLP operation. The scale bar represents 25 μm. (F) The expression of ribophagy-related proteins in Jurkat T cells stimulated with LPS was detected by WB. (G) The expression of ribophagy-related proteins in splenic CD4$^+$ T cells stimulated with LPS was detected by WB. (H) The expression of ribophagy-related proteins in splenic CD4$^+$ T cells was detected by WB after CLP operation. (I) The morphological changes of CD4$^+$ T lymphocyte organelles and ribophagy in sepsis were determined by TEM. The red stars indicate the ribosomes, and the blue arrows represent autophagosomes. The scale bars represent 500 nm (top) and 100 nm (bottom). Data were expressed as means ± SEM. An unpaired 2-sided Student's *t* test was applied to test the statistical significance. $^*P < 0.05$, $^{***}P < 0.001$. $^{\#\#\#}P < 0.001$ compared with the empty-LPS group.

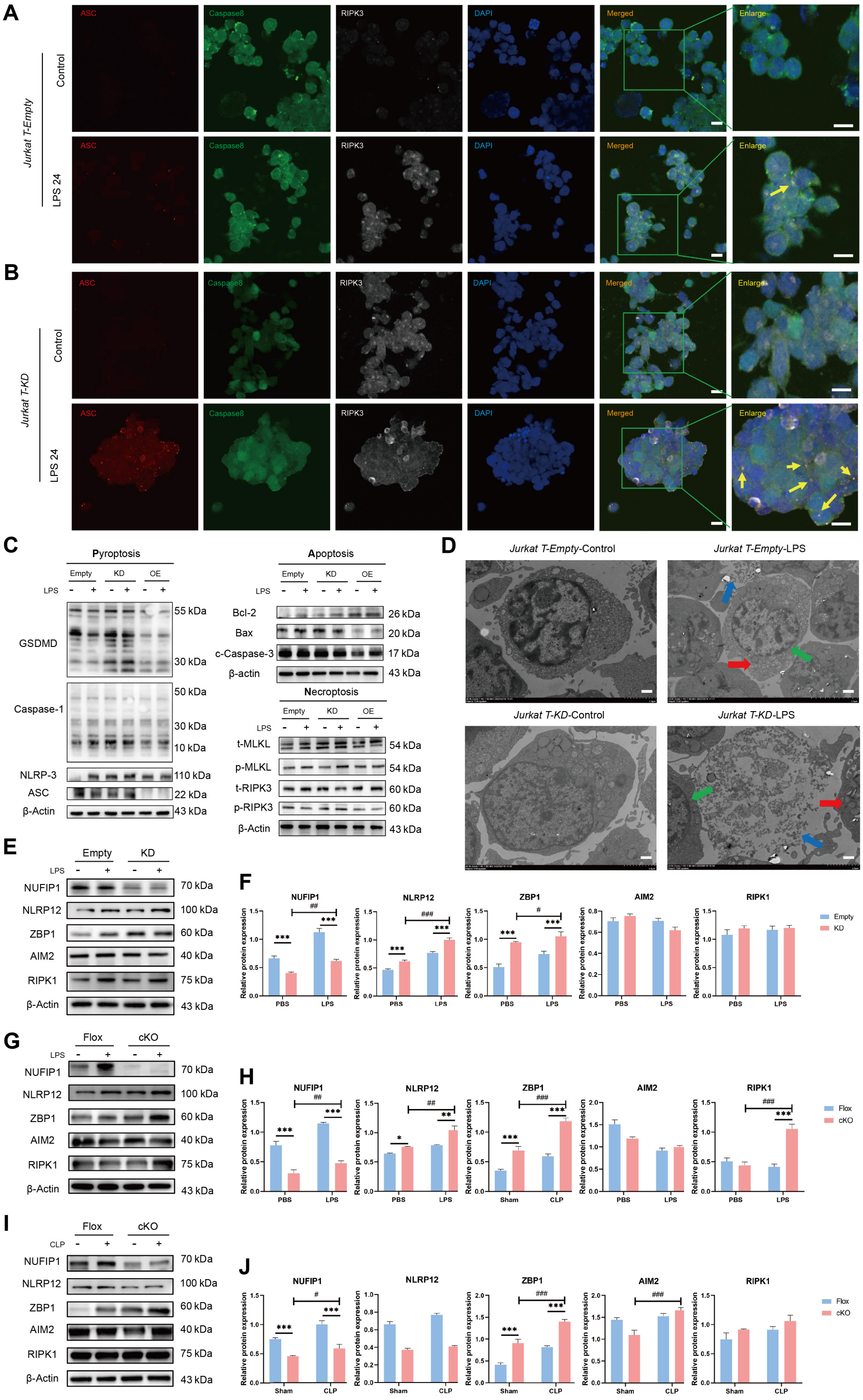


**Fig. 3.** Effect of NUFIP1-mediated ribophagy on ZBP1-PANoptosis in sepsis. (A) The expression and colocalization of ASC/caspase-8/RIPK3 in Jurkat-empty cells stimulated with LPS were determined by LSCM. The yellow arrows represent PANoptosomes, and the scale bar represents 25 μm. (B) The expression and colocalization of ASC/caspase-8/RIPK3 in Jurkat-KD cells stimulated with LPS were determined by LSCM. The yellow arrows represent PANoptosomes, and the scale bar represents 25 μm. (C) The expression of PANoptosis-related proteins in Jurkat T cells after transfection with lentivirus was detected by WB. (D) The morphological characteristics of PANoptosis in Jurkat T cells after transfection with lentivirus were observed by TEM. The red arrow denotes the intracellular vesicles associated with pyroptosis; the green arrow signifies the nuclear condensation and chromatin shrinkage characteristic of cell apoptosis; the blue arrow indicates the loss of cell membrane integrity in necroptosis; and the scale bar represents 500 nm. (E and F) Expression of 3 key proteins mediating PANoptosome formation in Jurkat T cells of empty and KD groups under LPS stimulation by WB. $n = 3$ technical repetitions. (G and H) Expression of key proteins mediating PANoptosome formation in splenic CD4$^+$ T cells of the Flox and cKO groups under LPS stimulation by WB. $n = 3$ technical repetitions. (I and J) The expression of key proteins mediating PANoptosome formation in splenic CD4$^+$ T cells of the Flox and cKO groups was examined by WB after CLP operation. $n = 3$ technical repetitions. Data were expressed as means ± SEM. A 2-way ANOVA test was applied to test the statistical significance. $^{***}P < 0.001$ compared with the empty/Flox group; $^{\#}P < 0.05$, $^{\#\#}P < 0.01$, $^{\#\#\#}P < 0.001$ compared with the KD-PBS/cKO-PBS/cKO-sham group.

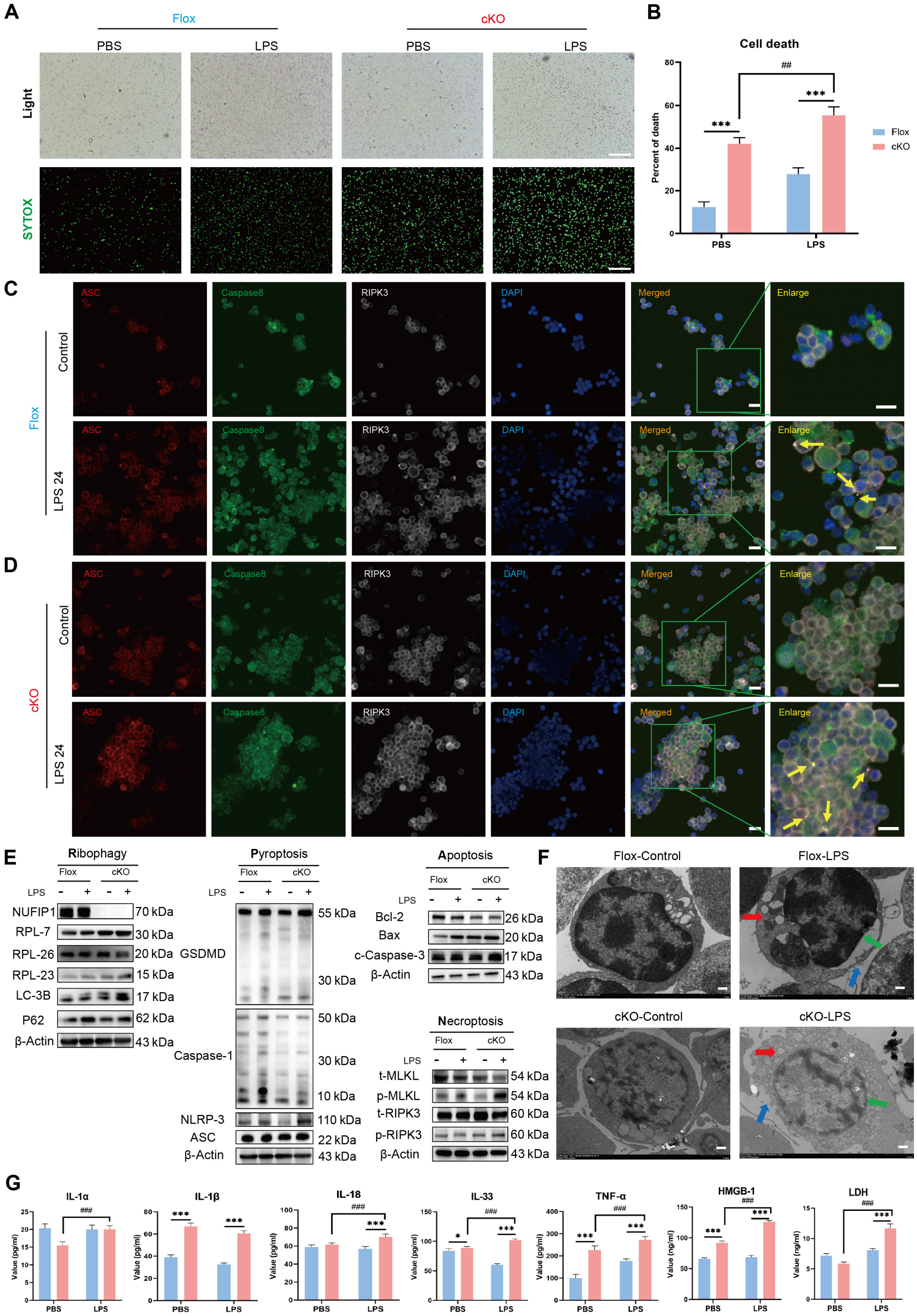


**Fig. 4.** Impact of conditional deletion of *NUFIP1* on PANoptosis of splenic CD4$^+$ T lymphocytes in septic mice. (A and B) The necrosis of splenic CD4$^+$ T lymphocytes in mice stimulated with LPS was detected by SYTOX-Green. The scale bar represents 100 μm. $n = 3$ technical repetitions. (C) The expression and colocalization of ASC/caspase-8/RIPK3 in splenic CD4$^+$ T lymphocytes of Flox mice stimulated with LPS were detected by LSCM. The yellow arrows represent PANoptosomes, and the scale bar represents 25 μm. (D) The expression and colocalization of ASC/caspase-8/RIPK3 in splenic CD4$^+$ T lymphocytes of cKO mice stimulated with LPS were detected by LSCM. The yellow arrows represent PANoptosomes, and the scale bar represents 25 μm. (E) Expression of PANoptosis-related proteins in splenic CD4$^+$ T lymphocytes of cKO mice stimulated with LPS under WB. (F) Morphological characteristics of PANoptosis in splenic CD4$^+$ T lymphocytes of Flox and cKO mice stimulated with LPS under TEM. The red arrow denotes the intracellular vesicles associated with pyroptosis; the green arrow signifies the nuclear condensation and chromatin shrinkage characteristic of cell apoptosis; the blue arrow indicates the loss of cell membrane integrity in necroptosis; and the scale bar represents 500 nm. (G) Cytokine levels in the culture supernatant of splenic CD4$^+$ T cells were measured by ELISA in Flox and cKO groups. $n = 6$ technical repetitions. Data were expressed as means ± SEM. A 2-way ANOVA test was applied to test the statistical significance. $^*P < 0.05$, $^{***}P < 0.001$ compared with the Flox group. $^{\#\#}P < 0.01$, $^{\#\#\#}P < 0.001$ compared with the cKO-PBS group.

cell counting kit-8 (CCK-8), showing a significant decrease in proliferation following LPS stimulation or CLP in both the Flox and cKO groups (Fig. 5A and Fig. S4A). ELISA indicated a shift toward Th2 differentiation in sepsis, leading to an immunosuppressive state. Subsequent experiments demonstrated a more pronounced reduction in IL-2 and interferon-γ (IFN-γ) secretion by splenic $CD4^+$ T lymphocytes after *NUFIP1* cKO, reinforcing the Th2 differentiation trend (Fig. 5B and Fig. S4B). Flow cytometry results showed a significant decrease in the proportions of $CD3^+$ T and $CD3^+CD4^+$ T lymphocytes in peripheral blood mononuclear cells (PBMCs) post-CLP operation, with a more substantial decline observed after *NUFIP1* knockout (Fig. 5C to F). Moreover, flow cytometry analysis of $CD4^+$ T cell subsets demonstrated that LPS stimulation and CLP-induced sepsis resulted in an elevated proportion of T helper 2 (Th2)/Th1 cell subsets (Fig. 5G and H and Fig. S4C and D). Furthermore, the percentages of regulatory T (Treg) and Th17 cell subsets, indicative of immunosuppressive function, were also significantly increased (Fig. 5I to L and Fig. S4E to H). Following cKO of the *NUFIP1*, the alterations in the proportions of the aforementioned lymphocyte subgroups were further exacerbated, suggesting a more pronounced impairment of $CD4^+$ T lymphocyte immune function. Hematoxylin and eosin (H&E) staining of heart, lung, liver, and kidney tissues revealed exacerbated damage following *NUFIP1* cKO (Fig. 5M to Q). Survival rate analysis highlighted a significant reduction in 1-week survival rates post-CLP, with a further decrease observed in $Cd4^{cre}Nufip1^{fl/fl}$ mice compared to $Nufip1^{fl/fl}$ mice (Fig. 5R).

## cGAS-STING signaling is critical for NUFIP1-mediated ribophagy in regulating $CD4^+$ T lymphocyte PANoptosis upon sepsis challenge

As illustrated in Fig. S5A, a total of 904 up-regulated proteins and 954 down-regulated proteins were identified, with the top 20 proteins detailed in Table S3. Subsequently, cluster analysis was conducted on the relative protein content in each sample, revealing variations in protein expression between different sample comparisons through a cluster heat map. Interestingly, significant differences were observed in Jurkat T cells between the normal and KD groups under LPS stimulation (Fig. S5B). A comprehensive bioinformatics analysis was conducted based on these differentially expressed proteins. Gene ontology (GO) analysis, depicted in Fig. 6A, highlighted that upon *NUFIP1* KD in Jurkat T cells under LPS stimulation, biological processes such as translation regulation, ribosome generation, autophagy, protein synthesis, folding, and degradation were impacted. Notably, alterations were observed in cell components like ribosomes, nuclei, peroxisomes, and autophagosomes, along with shifts in molecular functions related to protein folding reactions, ribosome assembly, and degradation, suggesting that NUFIP1-mediated ribophagy significantly influenced $CD4^+$ T lymphocyte protein quality control, ribosome homeostasis, and autophagy. Kyoto encyclopedia of genes and genome (KEGG) pathway enrichment analysis (Fig. 6B) revealed that upon *NUFIP1* KD in Jurkat T cells after LPS stimulation, signaling pathways associated with pathogen infections (*Yersinia*, *Shigella*, *Salmonella*, *Escherichia coli*, *Coronavirus*), endoplasmic reticulum protein processing, autophagy, apoptosis, and cGAS-STING signaling were significantly enriched. Combining the identified top 20 differentially expressed proteins and KEGG pathway enrichment analysis, it was inferred that the cGAS-STING signaling pathway might play a potential role in $CD4^+$ T lymphocyte PANoptosis, regulated by NUFIP1-mediated ribophagy in sepsis.

WB analysis showed that stimulation with LPS resulted in up-regulated expression of NUFIP1, ZBP1, and proteins associated with cGAS-STING, including cGAS, phosphorylated STING (p-STING), phosphorylated TBK1 (p-TBK1), and phosphorylated IRF3 (p-IRF3) (Fig. 6C). Knocking down the *NUFIP1* gene in Jurkat T cells led to a significant reduction in NUFIP1 expression, while significantly increased levels of ZBP1 protein and the cGAS-STING signaling-related proteins were observed (Fig. 6D, $P < 0.05$). Conversely, overexpression of the *NUFIP1* gene in Jurkat T cells enhanced NUFIP1 expression but decreased the protein levels of ZBP1 and cGAS-STING pathway. Subsequently, WB was employed to assess the expression of proteins in splenic $CD4^+$ T lymphocytes of $Cd4^{cre}Nufip1^{fl/fl}$ and $Nufip1^{fl/fl}$ mice. Upon *NUFIP1* knockout, regardless of LPS stimulation or CLP operation, a decrease in NUFIP1 expression was observed, while ZBP1 and cGAS-STING-related proteins increased significantly, with statistical significance (Fig. 6E and F and Fig. S6A and B).

## Polysome profiling and Co-IP reveal the molecular mechanism underlying NUFIP1-mediated ribophagy in activating cGAS-STING signaling

To explore the molecular mechanism underlying ribophagy-induced cGAS-STING activation, we investigated ribosomal dynamics and protein interactions. Polysome profiling was used to assess ribosomal collision events in septic $CD4^+$ T lymphocytes. As illustrated in Fig. 6G and H, LPS-stimulated Jurkat T cells and $CD4^+$ T lymphocytes isolated from CLP mice exhibited significantly elevated ribosomal collision frequencies compared to untreated controls. Genetic modulation experiments revealed that small interfering RNA (siRNA)-mediated *NUFIP1* KD exacerbated ribosomal collisions. In contrast, lentiviral *NUFIP1* overexpression effectively mitigated this response (Fig. 6I). Consistent observations were observed in $CD4^+$ T lymphocytes isolated from septic mice (Fig. S6C and D). In addition, Co-IP assays were performed to examine physical interactions between NUFIP1 and STING. Significant NUFIP1-STING complex formation was observed in both Jurkat T cells and murine $CD4^+$ T lymphocytes, as evidenced by immunoblotting of precipitated complexes (Fig. 6J and Fig. S6E).

## Impact of cGAS-STING modulation on PANoptosis and immune function of $CD4^+$ T cells, multiple organ injury, and 1-week survival in septic mice

To further investigate the potential role of the cGAS-STING signaling in regulating PANoptosis, we used a specific small-molecule inhibitor to modulate the related pathways in these current experiments. SN-011 is a specific inhibitor of the cGAS-STING signaling that competitively binds to the STING protein, thereby inhibiting its activation. As shown in Fig. 7A and B and Fig. S7A and B, increasing concentrations of SN-011 resulted in a gradual weakening of STING protein expression, with a similar trend observed in downstream molecules TBK1 and IRF3. Notably, Jurkat T cells and splenic $CD4^+$ T lymphocytes in mice exhibited significant inhibition at concentrations of 20 and 5 μM, respectively. Compared with the Flox group,

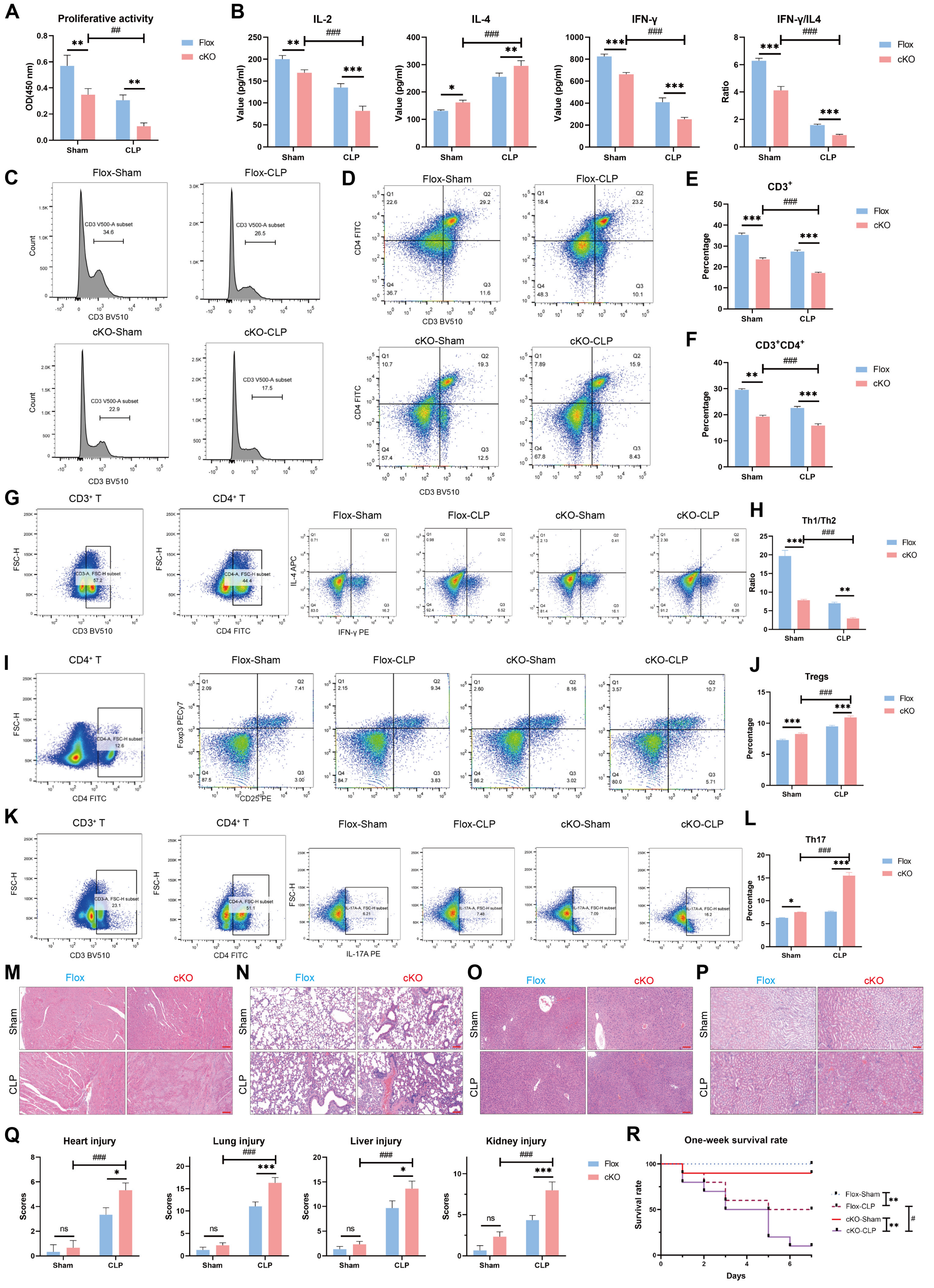


**Fig. 5.** Impact of conditional deletion of *NUFIP1* on immune response of CD4$^{+}$ T lymphocytes, organ injury, and the 1-week survival rate of mice in sepsis. (A) The proliferative activity of splenic CD4$^{+}$ T cells was measured by CCK-8 after CLP operation. $n = 3$ technical repetitions. (B) Serum cytokine levels in the Flox and cKO groups were measured by ELISA after CLP operation. $n = 4$ technical repetitions. (C and E) Proportion of CD3$^{+}$ T lymphocytes in the peripheral blood of mice in different groups detected by flow cytometry. $n = 3$ technical repetitions. (D and F) The proportion of CD3$^{+}$CD4$^{+}$ T lymphocytes in the peripheral blood of mice in different groups was detected by flow cytometry. $n = 3$ technical repetitions. (G and H) The ratio of Th1/Th2 of splenic CD4$^{+}$ T cells was detected by flow cytometry. $n = 3$ technical repetitions. (I and J) Percentage of Tregs of splenic CD4$^{+}$ T cells detected by flow cytometry. $n = 3$ technical repetitions. (K and L) Percentage of Th17 cells of splenic CD4$^{+}$ T cells detected by flow cytometry. $n = 3$ technical repetitions. (M to Q) H&E staining assessment of various organ lesions, including heart (M), lung (N), liver (O), and kidney (P) in different groups of mice. The scale bar represents 50 μm. $n = 3$ biological independent samples. (R) One-week survival curves of different groups of mice (**$P < 0.01$ compared with the sham group; $^{\#}P < 0.05$ compared with the Flox-CLP group). $n = 10$ biological independent samples. Data were expressed as means ± SEM. A 2-way ANOVA test was applied to test the statistical significance. *$P < 0.05$, **$P < 0.01$, ***$P < 0.001$ compared with the Flox group. $^{\#\#}P < 0.01$, $^{\#\#\#}P < 0.001$ compared with the cKO-sham group.

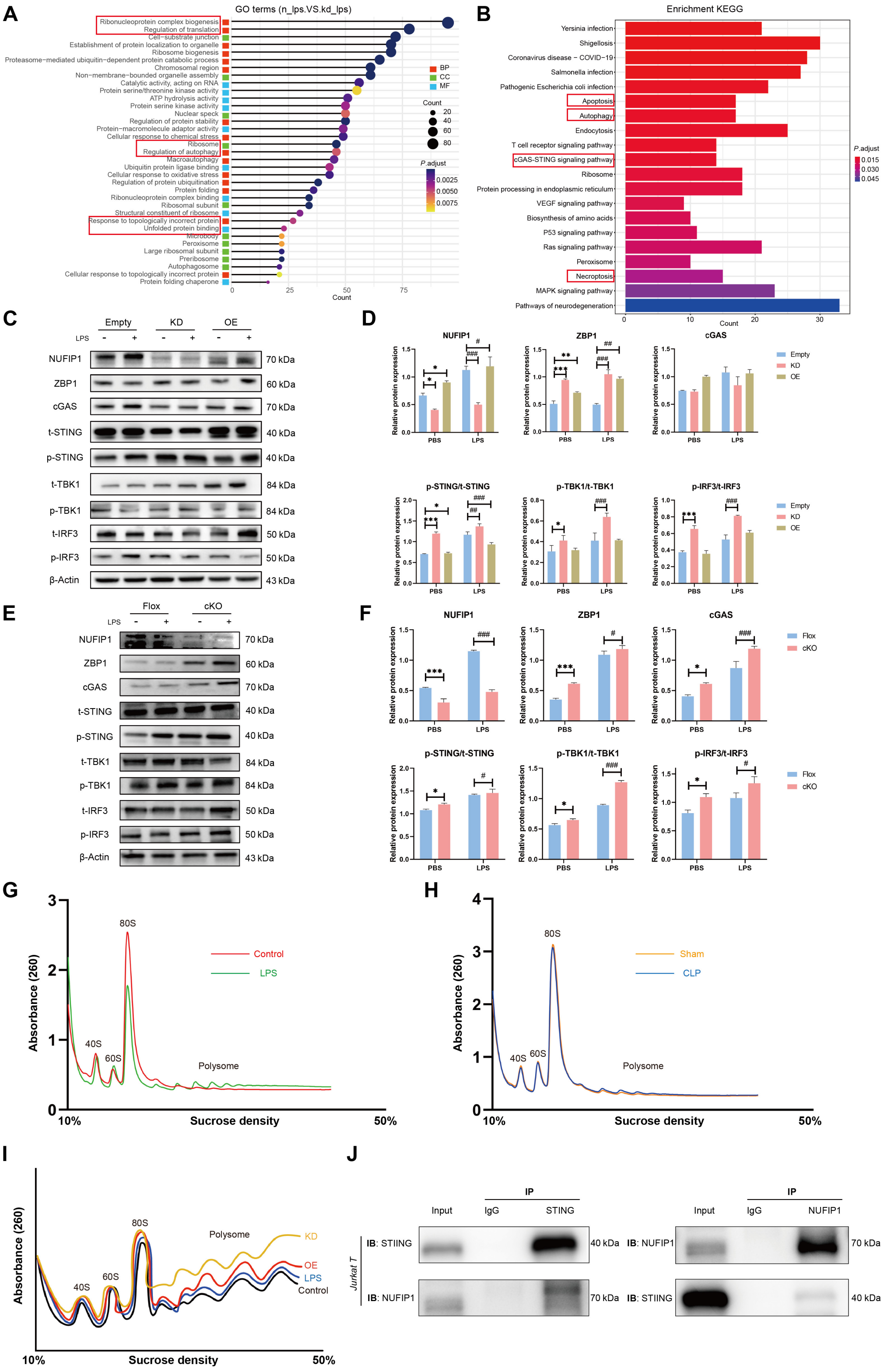

**Fig. 6.** *NUFIP1* deficiency disrupts ribophagy and ribosomal collision to trigger cGAS-STING-dependent PANoptosis in septic CD4$^+$ T lymphocytes. (A) Differentially expressed protein GO pathway enrichment maps of Jurkat T cells in the normal control and KD groups stimulated with LPS. (B) Differentially expressed protein KEGG pathway enrichment map of Jurkat T cells in the normal control and KD groups stimulated with LPS. (C and D) Expression of cGAS-STING signaling-related proteins in different groups of Jurkat T cells under LPS stimulation by WB. $n = 3$ technical repetitions. (E and F) Expression of cGAS-STING signaling-related proteins in splenic CD4$^+$ T cells of the Flox and cKO groups under LPS stimulation by WB. $n = 3$ technical repetitions. (G) Ribosomal collision events of Jurkat T cells assessed by polysome profiling under PBS or LPS. (H) Ribosomal collision events of CD4$^+$ T cells assessed by polysome profiling under sham or CLP. (I) The effects of *NUFIP1* gene interference on ribosomal collisions within Jurkat T cells were assessed by polysome profiling. (J) The interaction between NUFIP1 and STING in Jurkat T cells was detected by Co-IP. Data were expressed as means ± SEM. A 2-way ANOVA test was applied to test the statistical significance. $^*P < 0.05$, $^{**}P < 0.01$, $^{***}P < 0.001$ compared with the empty-PBS/Flox-PBS group; $^{\#}P < 0.05$, $^{\#\#}P < 0.01$, $^{\#\#\#}P < 0.001$ compared with the empty-LPS/Flox-LPS group.

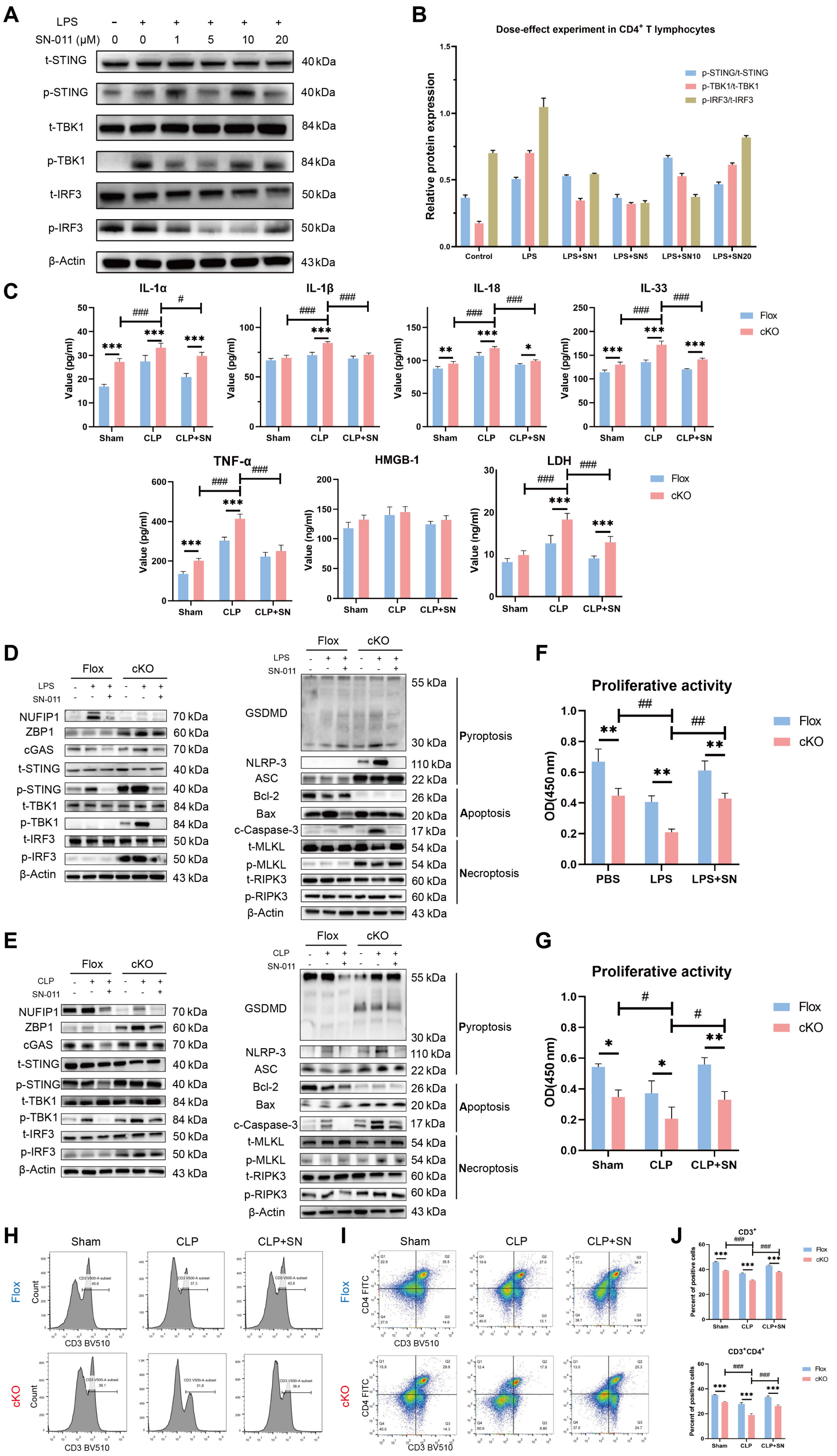

**Fig. 7.** Regulatory effects of cGAS-STING signaling on CD4$^+$ T lymphocyte PANoptosis and immune response in septic mice. (A and B) The optimal concentration of SN-011 on splenic CD4$^+$ T lymphocytes under LPS stimulation was determined by WB. $n = 3$ technical repetitions. (C) Serum cytokine levels in the Flox and cKO groups were measured by ELISA after CLP. $n = 6$ technical repetitions. (D) The effects of SN-011 on the cGAS-STING pathway and PANoptosis-related protein expression in splenic CD4$^+$ T lymphocytes under LPS stimulation were detected by WB. (E) The effects of SN-011 on cGAS-STING signaling and PANoptosis-related protein expression in splenic CD4$^+$ T lymphocytes by WB after CLP. (F and G) The effect of SN-011 on the proliferative activity of splenic CD4$^+$ T lymphocytes in sepsis mice was measured by CCK-8. $n = 3$ technical repetitions. (H to J) The effect of SN-011 on the proportion of CD3$^+$ T (H) and CD3$^+$CD4$^+$ T lymphocytes (I) in the peripheral blood of mice of different groups was detected by flow cytometry. $n = 3$ technical repetitions. Data were expressed as means ± SEM. A 2-way ANOVA was applied to test the statistical significance. $^*P < 0.05$, $^{**}P < 0.01$, $^{***}P < 0.001$ compared with the Flox group. $^{\#}P < 0.05$, $^{\#\#}P < 0.01$, $^{\#\#\#}P < 0.001$ compared with the cKO-LPS/CLP groups.

serum levels of IL-1β, IL-18, IL-1α, IL-33, TNF-α, HMGB1, and LDH were significantly elevated in the *NUFIP1* cKO group. Following treatment with SN-011, marked decreases in the serum levels of these cytokines were observed in both the Flox and *NUFIP1* cKO groups (Fig. 7C). Then, WB was utilized to evaluate the impact of SN-011 on the $CD4^+$ T lymphocyte signaling pathway and PANoptosis-related protein expression during sepsis. As shown in Fig. S7C, a significant decrease in NUFIP1 expression was observed in Jurkat T cells in the KD group. Conversely, considerable up-regulation of ZBP1 and cGAS-STING-associated proteins, including cGAS, STING, TBK1, and IRF3, was observed. Besides, the expression of PANoptosis-related proteins, including GSDMD, NLRP3, ASC, Bax, c-caspase-3, p-MLKL, and p-RIPK3, was elevated in Jurkat T cells of the KD group. Treatment with SN-011 resulted in significant inhibition of cGAS-STING-related proteins, which was associated with reduced expression of PANoptosis-related proteins. Consistent results were observed in the expression of splenic $CD4^+$ T lymphocytes following LPS challenge in vitro and CLP operation in vivo (Fig. 7D and E).

Next, we sought to assess the impact of the cGAS-STING signaling pathway on T cell immune function, peripheral immunosuppression, multiple organ injury, and the prognosis of septic mice lacking the *NUFIP1* gene. Inhibition of the cGAS-STING signaling pathway by SN-011 led to significant restoration in the proliferation of splenic $CD4^+$ T lymphocytes, an increase in the proportions of $CD3^+$ T and $CD3^+CD4^+$ T cells in PBMCs, and a notable reduction in multi-organ damage (Figs. 7F to J and 8A to E). Furthermore, treatment with SN-011 significantly improved the survival rates of mice at 1 week, particularly in those with *NUFIP1* cKO (Fig. 8F and G).

## Clinical significance of ribophagy and PANoptosis in peripheral blood $CD4^+$ T lymphocytes of individuals with sepsis

To investigate alterations in PANoptosis in patients with and without septic complications, terminal deoxynucleotidyl transferase-mediated deoxyuridine triphosphate nick end labeling (TUNEL) and Hoechst 33258 staining methods were performed to identify apoptosis, while SYTOX-Green staining was utilized to detect cell necrosis. Results revealed a significant increase in the percentage of apoptosis and necrosis in $CD4^+$ T lymphocytes of septic patients compared to the control subjects (Fig. 8H to J). Meanwhile, WB was carried out to assess ribophagy and PANoptosis-related proteins in peripheral blood $CD4^+$ T lymphocytes of patients. Significant elevation of various ribophagy and PANoptosis markers, including LC-3B, NUFIP1, ZBP1, GSDMD, NLPR-3, ASC, Bax, c-caspase-3, p-MLKL, and p-RIPK3, was observed in septic patients, along with reduced levels of ribosomal proteins RPL7, RPL23, RPL26, and anti-apoptotic protein Bcl-2 (Fig. 8K). Besides, functional analysis of $CD4^+$ T lymphocytes in septic patients demonstrated decreased proliferative and secretion activities, Th2 polarization, and reduced proportions of $CD3^+$ T and $CD3^+CD4^+$ T lymphocytes, suggesting a pronounced immunosuppressive state in these patients (Fig. 8L to O).

## Discussion

In the current study, we found that NUFIP1-mediated ribophagy modulated $CD4^+$ T lymphocyte PANoptosis in sepsis, consequently augmenting T cell-mediated immunity and preserving systemic immune homeostasis. Mechanistically, the involvement of ZBP1-PANoptosomes and the cGAS-STING signaling cascade appeared crucial to this mechanism. Ribosomal collision within $CD4^+$ T lymphocytes and the interaction between NUFIP1 and STING proteins were critical for initiating the cGAS-STING signaling via NUFIP1-mediated ribophagy (Fig. 9). Clinical validation further supported these findings, showing increased PANoptosis and ribophagy in peripheral blood $CD4^+$ T lymphocytes from septic patients alongside compromised immune function.

The present study has advanced our understanding of PANoptosis in septic pathophysiology, suggesting the synergistic role of TNF-α and IFN-γ in driving inflammatory cell death and tissue injury [29]. Therapeutic targeting of the cytokine-mediated PANoptosis pathway could ameliorate outcomes in LPS-induced inflammatory models, with potential relevance for COVID-19-associated coagulopathy. A recent report linked PANoptosis to sepsis-associated encephalopathy [30], suggesting that cortical neuron PANoptosis could be orchestrated by toll-like receptor 9 via activation of p38–mitogen-activated protein kinase pathway. This paradigm has since inspired extensive investigations into PANoptosis-driven pathologies in severe complications, including disseminated intravascular coagulation, lung injury, cardiomyopathy, and acute kidney injury, collectively underscoring the therapeutic potential of PANoptosis modulation in the management of critical illness [31–36]. Despite these advancements, critical gaps persist in our understanding of PANoptosis dynamics in immune cells, particularly in sepsis. While many studies have predominantly focused on endothelial cells, neuronal cells, and hepatocytes, the possible role and regulatory mechanisms of PANoptosis in host immune response remain unexplored. In this regard, it has been reported that inhibition of mitochondrial DNA oxidation in macrophages could markedly attenuate PANoptosis, implying potential macrophage-centric therapeutic strategies [37–39]. Herein, our findings represent a paradigm shift by investigation of $CD4^+$ T lymphocyte PANoptosis in septic models. Through multi-dimensional analyses, we have uncovered novel links between PANoptotic T cell demise, abnormal immune response, and cytokine dysregulation, implicating T cell PANoptosis as a contributor to sepsis-induced immunosuppression.

Ribophagy, an emerging paradigm in organelle autophagy research, functions as a pivotal regulatory mechanism in RQCS. This process maintains proteostasis and cellular survival through selective degradation of aberrant ribosomes, constituting a critical line of defense against translational stress-induced apoptosis. Despite its fundamental biological importance, the pathophysiological roles of ribophagy in human diseases remain substantially unexplored, particularly regarding its regulatory capacity in sepsis-induced immune dysfunction. Our previous studies have demonstrated that NUFIP1-mediated ribophagy could attenuate $CD4^+$ T lymphocyte apoptosis via suppression of the endoplasmic reticulum stress-dependent apoptotic cascade, suggesting its potential involvement in broader cell death regulatory networks [11]. Given the functional interdependence between apoptosis and PANoptosis in the development of sepsis, we hypothesized that ribophagy might exert modulatory effects on $CD4^+$ T lymphocyte PANoptosis. In the current study, the results substantiated that ribophagy was associated with $CD4^+$ T lymphocytes' PANoptosis. Notably, NUFIP1-mediated ribophagy of $CD4^+$ T lymphocytes exhibited a significant impact on host immunosuppression status, multiple organ damage, and

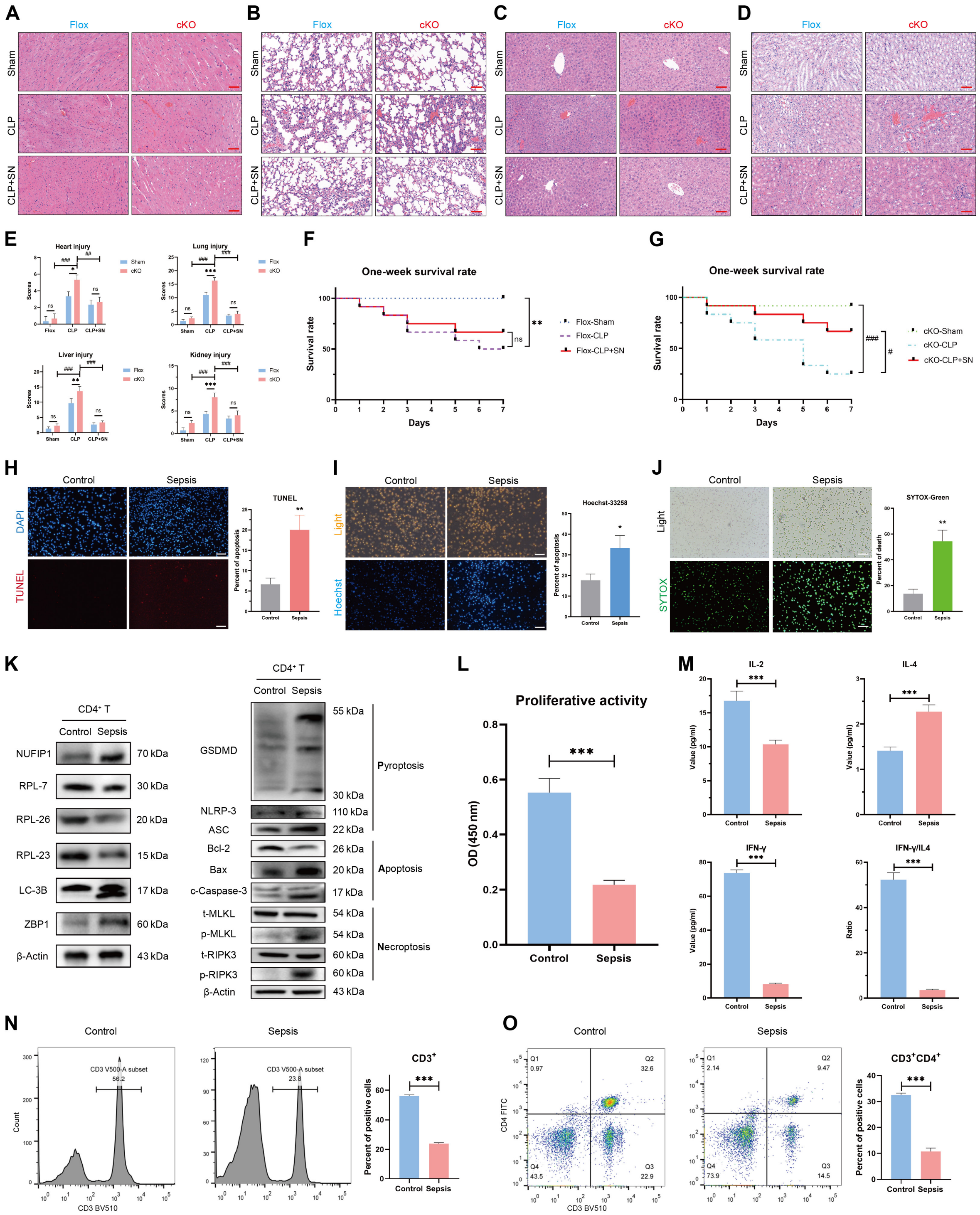


**Fig. 8.** Regulatory effects of cGAS-STING signaling on peripheral immune status in septic mice and ribophagy, PANoptosis, and immune function of peripheral blood CD4$^+$ T lymphocytes in sepsis patients. (A to E) H&E staining assessed the effects of SN-011 on multiple organ damage, including heart (A), lung (B), liver (C), and kidney (D) in different groups of mice. The scale bar represents 50 μm. $n = 3$ biological independent samples (two-way ANOVA was applied to test the statistical significance; $^{\#\#}P < 0.01$, $^{\#\#\#}P < 0.001$ compared with the cKO-CLP groups). (F and G) Impact of SN-011 on the 1-week survival rate of Flox (F) and cKO (G) mice. $n = 10$ biological independent samples ($^{**}P < 0.01$ compared with the sham group; $^{\#}P < 0.05$, $^{\#\#\#}P < 0.001$ compared with the cKO-CLP group). (H) The apoptosis of CD4$^+$ T lymphocytes in the peripheral blood of patients with and without sepsis was detected by TUNEL. The scale bar represents 100 μm. $n = 3$ technical repetitions. (I) The apoptosis of CD4$^+$ T lymphocytes in the peripheral blood of patients with and without sepsis was determined by Hoechst 33258. The scale bar represents 100 μm. $n = 3$ technical repetitions. (J) Necrosis of CD4$^+$ T lymphocytes in the peripheral blood of patients with and without sepsis was detected by SYTOX-Green. The scale bar represents 100 μm. $n = 3$ technical repetitions. (K) The expression of ribophagy and PANoptosis-related proteins in the peripheral blood CD4$^+$ T lymphocytes of patients with and without sepsis was analyzed by WB. (L) The proliferative activity of CD4$^+$ T lymphocytes in the peripheral blood of patients with and without sepsis was detected by CCK-8. $n = 3$ technical repetitions. (M) Serum cytokine levels in patients with and without sepsis were measured by ELISA. $n = 5$ technical repetitions. (N and O) The proportions of CD3$^+$ T (N) and CD3$^+$CD4$^+$ T (O) lymphocytes in the peripheral blood of patients with and without sepsis were analyzed by flow cytometry. $n = 3$ technical repetitions. Data were expressed as means ± SEM. An unpaired 2-sided Student's $t$ test was applied to test the statistical significance. $^*P < 0.05$, $^{**}P < 0.01$, $^{***}P < 0.001$.

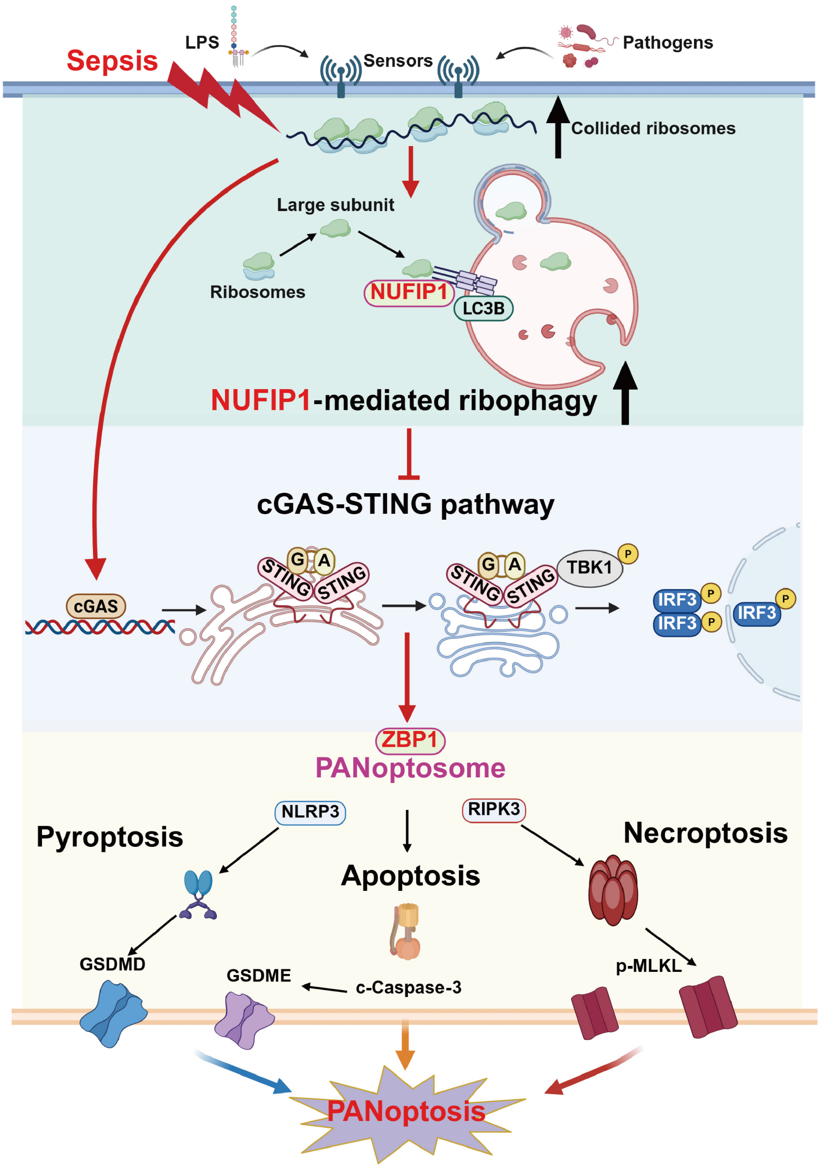


**Fig. 9.** Mechanism schematic diagram of the current study. Illustrations were created with BioRender.

sepsis prognosis. These observations further underscore the pleiotropic protective effects of ribophagy in sepsis pathophysiology. NUFIP1 is the first confirmed receptor mediating mammalian ribophagy [27]. It has been demonstrated that NUFIP1 interacts with LC3B to facilitate ribophagy and promote cell survival upon starvation. Recently, Rpl12 has been identified as a highly conserved ribophagy receptor from yeast to mammals, elucidating its mechanism of action in ribophagy under starvation conditions [40]. Thus, further investigation is warranted to determine whether Rpl12 is involved in ribophagy and PANoptosis of immune cells in sepsis.

The PANoptosome serves as a molecular scaffold for PANoptosis execution, coordinating the assembly of key regulatory proteins [41]. Currently, 4 critical regulators of PANoptosome formation have been identified, including ZBP1, AIM2, RIPK1, and NLRP12 [28,42–44]. Of these, ZBP1 functions as the primary upstream regulator, operating as a pathogen-associated molecular pattern recognition receptor that initiates PANoptosome assembly through its NACHT and LRR domains. In this study, we performed immunoblot analysis of ZBP1, AIM2, RIPK1, and NLRP12 expressions in Jurkat T cells following *NUFIP1* KD and in splenic $CD4^{+}$T lymphocytes from $Cd4^{cre}Nufip1^{fl/fl}$ mice. Significant up-regulation of ZBP1 protein expression was observed post-*NUFIP1* depletion, while AIM2, RIPK1, and NLRP12 levels remained unchanged. Thus, the differential regulation suggests that ZBP1 might play a unique role in PANoptosis activation under septic conditions. Subsequent Co-IP experiments further validated the intimate interaction between NUFIP1 and ZBP1. The pathological relevance of ZBP1-mediated PANoptosis is well documented across multiple diseases, including asthma, acute myeloid leukemia, ultraviolet radiation-induced skin damage, tumorigenesis, and spinal cord injury [45–48]. A recent report indicated that lactacemia-induced histone deacetylation could activate cold-inducible RNA-binding proteins in macrophages, in turn triggering PANoptosis in pulmonary vascular endothelial cells, resulting from sepsis [49]. This work corroborates our observation of ZBP1 up-regulation in immune cells and identifies a novel cross-talk between metabolic dysregulation and PANoptosis

execution. Moreover, ZBP1 interacts with cGAS through its Zα2 domain, establishing a liquid–liquid phase separation within infected fibroblasts. This process facilitates the recruitment of TBK1-RIPK1, thereby sustaining IFN-β expression and playing a critical role in antiviral immunity [50]. This investigation partially elucidates the potential mechanism by which ZBP1-PANoptosome modulates the activation of the cGAS-STING pathway in the setting of sepsis. In summary, the convergence of these findings positions ZBP1 as a central hub in septic immunopathology, where its dual roles in pathogen sensing and cell death execution create therapeutic targeting opportunities.

Mechanistically, we proposed a regulatory role of cGAS-STING signaling axis in ribophagy-dependent modulation of T lymphocyte PANoptosis based on the following evidence. Firstly, the sequencing of the top 20 differentially expressed proteins revealed multiple cGAS fragments. Besides, KEGG pathway analysis highlighted significant enrichment of the cGAS-STING signaling pathway. Finally, cGAS, as a canonical cytosolic DNA sensor, detects pathogen-derived double-stranded DNA (dsDNA) and endogenous damage-associated molecular patterns released during sepsis-induced cellular stress. This signaling transduction might contribute to the pathogenesis of sepsis, where pathogens disrupt homeostasis through membrane permeabilization and an inflammatory cascade, implying biological plausibility for pathway involvement [51]. Herein, significant up-regulation of cGAS, p-STING, p-TBK1, and p-IRF3 was observed following *NUFIP1* KD in Jurkat T cells and $Cd4^{cre}Nufip1^{fl/fl}$ mice. Several studies have indicated that the cGAS-STING pathway operates through a quaternary signaling cascade in sepsis, including pathogen recognition, inflammatory response, initiation of cellular demise, and immune response [52–54]. Therefore, the targeted modulation of this pathway via small-molecule inhibitors or gene editing may offer a novel approach for immune precision therapy in sepsis.

Recent studies have demonstrated that viral protein translation stress disrupts ribosomal homeostasis through collision-induced translational arrest, thereby activating cGAS via dsDNA sensing [24]. The pathogen–nucleic acid interaction results in a feedback loop where ribosome collisions not only amplify cGAS activation thresholds but also induce autophagic degradation of collided ribosomes. Our experiments employed polysome profiling and Co-IP assays to delineate the molecular interplay between NUFIP1-mediated ribophagy and cGAS-STING activation. It was found that sepsis induced marked ribosome collisions in $CD4^+$ T lymphocytes, which aligned with previous studies indicating that ribosome dynamics could exert dual effects as both cGAS co-activators and autophagy inducers. Mechanistic studies uncovered a previously unrecognized regulatory axis wherein NUFIP1 physically interacted with STING. To further corroborate this finding, SN-011, a small-molecule competitive inhibitor of STING, was used in subsequent studies. It has been demonstrated that SN-011 specifically binds to the transmembrane domain of STING. Through hydrophobic interactions and hydrogen bond networks, SN-011 stabilizes STING in an inactive monomeric conformation, thereby inhibiting its endoplasmic reticulum-to-Golgi apparatus transport [55]. Moreover, SN-011 occupies the STING dimer interface, thus preventing cGAMP-induced STING dimer formation [56]. In this study, GAS-STING signaling inhibition by SN-011 significantly attenuated T cell PANoptosis in sepsis, concurrently restoring proliferation and secretory function. Compared with septic mice without SN-011 treatment, there was a marked reduction in the severity of organ damage and a significant improvement in the 1-week survival rate of treated mice subjected to septic challenge.

Clinical validation was implemented to investigate the occurrence of PANoptosis and ribophagy in peripheral blood $CD4^+$ T lymphocytes among septic patients. We found that the levels of NUFIP1 and ZBP1 in the $CD4^+$ T lymphocytes of septic patients significantly increased compared to those without sepsis, corresponding to experimental data in murine models, thereby underscoring their conserved biomarker potential for the diagnosis of sepsis. Moreover, significant elevation in PANoptosis and decreased immune function of $CD4^+$ T lymphocytes were observed in the peripheral blood of septic patients, suggesting a pathophysiological axis linking ribophagy-mediated cell death to lymphocyte dysfunction in sepsis. These clinical observations provide a mechanistic framework for developing dual-modality therapeutics targeting immunosuppression and PCD.

Nevertheless, our findings should be considered in light of several limitations. Firstly, our study is contextualized within $CD4^+$ T cell responses, reflecting their dominant role in sepsis pathogenesis. However, $CD8^+$ effector T cells may contribute to late-phase immune exhaustion and warrant future investigation. This limitation does not diminish the core mechanism but highlights a pathway for broader exploration. Secondly, while CLP provides a validated platform for initial PANoptosis characterization, its integration with sterile and respiratory models, alongside human ex vivo data, may ensure that the mechanistic insights translate to the spectrum of human sepsis pathophysiology, including heterogeneous triggers like viral infections or tissue injury. Thirdly, the use of Jurkat T cells in proteomic analysis may limit the generalizability of our data to $CD4^+$ T lymphocytes within murine spleens. Fourthly, the precise mechanism by which the NUFIP1-STING complex regulates the interplay between ribophagy and the cGAS-STING pathway remains to be fully elucidated, necessitating further investigation. Fifthly, while we corroborated the critical role of ZBP1 in NUFIP1-mediated ribophagy associated with $CD4^+$ T lymphocytes' PANoptosis in sepsis, the precise interactional dynamics and underlying molecular mechanisms between these proteins remain to be elucidated. Advanced structural biology approaches may facilitate the resolution of these complexities. Lastly, the diagnostic potential of crucial proteins associated with ribophagy and PANoptosis in the risk stratification and prognostic prediction of sepsis patients requires thorough validation in multicenter, large-scale clinical trials.

## Conclusion

Overall, our findings provide compelling evidence that sepsis can induce $CD4^+$ T lymphocyte PANoptosis. At the same time, NUFIP1-mediated ribophagy serves as a protective mechanism, alleviating immune dysfunction, mitigating organ lesions, and improving survival rate post-septic challenge. Mechanistically, ribosomal stalling within $CD4^+$ T lymphocytes and the interaction between NUFIP1 and STING proteins play a potential role in initiating the cGAS-STING cascade through ribophagy. Taken together, the present study unveils a previously unrecognized regulatory axis linking $CD4^+$ T lymphocyte ribophagy and PANoptosis to sepsis-induced immunoparalysis, which might be valuable to develop precision immunomodulatory strategies targeting both excessive inflammatory response and host immune dysfunction in this life-threatening condition.

## Materials and Methods

### Cell culture

Jurkat T cells were procured from Chinese Type Culture Collection (CTCC, Shanghai, China). The lentiviral vectors containing the *NUFIP1* gene KD and overexpression were developed by GeneChem Co. Ltd. (Shanghai, China). These lentiviruses were utilized to modulate the expression of the NUFIP1 protein in the Jurkat T cells through transfection following the manufacturer's guidelines. Cell culture medium consisted of RPMI 1640 (Gibco, Waltham, USA) supplemented with 100 U/ml penicillin (Solarbio, Beijing, China), 100 μl/ml streptomycin (Solarbio), and 10% fetal bovine serum (Gibco, Waltham, USA). The cells were maintained in a 5% $CO_2$, 37 °C humidified incubator (Napco, Japan) during the culture process.

### Animals

Male C57 wild-type mice, aged 6 to 8 weeks, were procured from SiBeiFu Company (Beijing, China). $CD4^+$ T lymphocyte-specific *NUFIP1* cKO mice ($Cd4^{cre}Nufip1^{fl/fl}$) and their corresponding control mice ($Nufip1^{fl/fl}$) were generated and raised to 6 to 8 weeks of age by Cyagen Biosciences Inc. (Suzhou, Jiangsu, China). The mice weighed approximately 20 g, meeting the specific pathogen-free grade requirements. The laboratory animal housing room was maintained at a temperature of 25 °C, with 55% humidity, and unrestricted access to food and water. All experimental protocols were conducted by the International Guidelines for the Care and Use of Laboratory Animals and were approved by the Animal Ethics Committee of the Chinese PLA General Hospital under the approval number 2024-X20-40, Beijing, China.

### Procedures for CLP

Before abdominal disinfection of a 2.5-$cm^2$ surgical field, anesthesia was induced through intraperitoneal administration of 4% chloral hydrate combined with sodium pentobarbital (60 mg/kg body weight). Following aseptic preparation, a midline longitudinal incision measuring 1.0 cm was created through the abdominal wall. The distal cecum was partially ligated approximately 1 cm from its apex and then perforated using a 21-gauge hypodermic needle to facilitate fecal extrusion for sepsis induction. After cecal repositioning, sequential closure of the peritoneal and cutaneous layers was performed. Besides, a subcutaneous injection of 0.9% saline solution (1 ml) was administered in the cervical area. Model validation criteria included behavioral observations (reduced activity, diarrheal presentation, and piloerection) combined with survival analysis showing 30% to 50% mortality within the 7-d postoperative period following CLP operation. Sham-operated controls received identical surgical interventions, excluding cecal ligation and perforation.

### Isolation of mouse splenic $CD4^+$ T lymphocytes

Splenic tissues were aseptically harvested from euthanized mice and immediately rinsed multiple times with ice-cold phosphate-buffered saline (PBS). Following mechanical dissociation through sterile mesh filtration, the homogenized tissue underwent washing with PBS. The resultant cellular suspension was mixed with murine-specific lymphocyte separation medium at a 1:1 volumetric ratio, followed by density gradient centrifugation (15 min at 3,000 rpm) for mononuclear cell isolation. $CD4^+$ T lymphocytes were enriched using magnetic-activated cell sorting (MACS), magnetic separation (MS) columns, and anti-CD4 microbeads according to the manufacturer's instructions (Miltenyi Biotec, Bergisch Gladbach, Germany). Purified lymphocytes were maintained in RPMI 1640 complete medium under standard culture conditions (37 °C, 5% $CO_2$). For cellular activation, splenic $CD4^+$ T cells underwent 24-h pretreatment with 5 μg/ml concanavalin A (Con A; Solarbio, Beijing, China) before functional assays.

### Time– and dose–response experiments of LPS stimulation

To determine optimal stimulation parameters, Jurkat T cells and splenic $CD4^+$ T lymphocytes were exposed to 1,000 ng/ml LPS (Sigma-Aldrich, St Louis, MO) for multiple time intervals (6 to 72 h), with PBS-treated controls serving as baseline references. Subsequent dose–response profiling was conducted by treatment with LPS ranging from 0 to 1,000 ng/ml during the 24-h window identified as physiologically relevant. Following parameter optimization using this sequential screening approach, cellular samples were harvested at predetermined experimental endpoints for downstream molecular analyses.

### TUNEL apoptosis assay

Apoptosis detection was implemented using a commercial TUNEL kit (Beyotime Biotechnology, China) following standardized procedures. Key steps of the protocol included the following: (a) cell preparation: pre-processed $CD4^+$ T lymphocyte suspensions ($>2 \times 10^6$ cells) underwent sequential PBS washing cycles; (b) fixation and permeabilization: cellular fixation was achieved through 30-min incubation in 4% paraformaldehyde (PFA), followed by membrane permeabilization using 0.3% Triton X-100/PBS (5 min, room temperature); (c) staining workflow: after reconstituting kit components per manufacturer specifications, samples were washed with PBS twice before application of 50 μl of TUNEL reaction mixture. Samples were incubated in the dark at 37 °C for 1 h and subsequently rinsed with PBS; (d) analytical preparation: cells were resuspended in 250 to 500 μl of PBS for fluorescence microscopy.

### SYTOX-Green staining experiment

A SYTOX-Green (Thermo Fisher, USA) working solution was prepared by diluting SYTOX-Green nucleic acid dye stock (5 mM) to 50 nM in dimethyl sulfoxide for subsequent applications. Following cell culture, cells were stained with 100 nM SYTOX-Green working solution and incubated in a cell culture incubator for the designated period. Subsequently, at least $2 \times 10^6$ cells were harvested in flow tubes, rinsed thrice with PBS buffer solution, and centrifuged at 1,500 rpm for 5 min each. The supernatant was discarded, retaining 100 μl of the residual liquid, and the cells were resuspended thoroughly. For slide preparation, 40 μl of the cell suspension was dispensed onto the slide and covered with a coverslip. Finally, the total cell counts were determined using an optical microscope, and SYTOX-Green-positive cells were visualized and imaged under a fluorescence microscope.

### WB analysis

T cell populations ($>6 \times 10^6$ cells per Eppendorf tube) underwent lysis in ice-cold radioimmunoprecipitation assay buffer

supplemented with protease inhibitor cocktail (1:50) and phosphatase inhibitor (1:100). (a) Cellular lysis was performed by intermittent vortexing during 30-min ice incubation, followed by 3 freeze–thaw cycles (liquid nitrogen/room temperature) and high-speed centrifugation (14,000*g*, 30 min, 4 °C) for supernatant collection. (b) For electrophoresis, protein lysates were denatured with a 4:1 ratio of sodium dodecyl sulfate (SDS)-loading buffer (95 °C, 5 min) and quantified via a bicinchoninic acid (BCA) assay standard curve. Samples were resolved through SDS–polyacrylamide gel electrophoresis (PAGE) (12% separating gel) under reducing conditions. (c) Immunoblotting was performed using semidry electrophoretic transfer to nitrocellulose membranes. Membranes were blocked with 5% bovine serum albumin (BSA)/tris-buffered saline with Tween 20 (TBST) blocking buffer for 1 h, followed by blocking with primary antibodies (1:1,000 dilution; Table S1) and β-actin (mouse monoclonal antibody) as loading control, and then incubated with species-matched horseradish peroxidase-conjugated secondary antibodies (1:5,000). Chemiluminescent detection was performed using Amersham Imager 600 (GE Healthcare).

## Laser scanning confocal microscopy

The colocalization of PANoptosis-related proteins was examined using laser scanning confocal microscopy (LSCM) (Leica, Mannheim, Germany). The procedure involved several steps, with each step followed by washing with PBS 3 times and centrifugation at 1,500 rpm for 5 min each time. Initially, a minimum of $1 \times 10^6$ cells were collected and centrifuged to remove the supernatant. The cells were then fixed with 4% PFA (500 μl) for 1 h, permeabilized with 0.3% Triton X-100 (400 μl) for 20 min, and blocked with 1% BSA solution (500 μl) for 1 h at room temperature. The primary antibodies ASC (1:200, Santa Cruz Biotechnology, Santa Cruz, CA) and caspase-8 (1:200, Proteintech) were then applied and incubated overnight at 4 °C. On the following day, a fluorescein-conjugated secondary antibody was introduced and incubated at room temperature for 1 h, followed by the addition of fluorescein-labeled RIP3 (1:200, Santa Cruz Biotechnology, Santa Cruz, CA), which was incubated for an additional hour at room temperature. After washing with PBS, the cells were mixed with the substrate. A slide was prepared by adding 20 μl of the cell suspension, combining it with 10 μl of 4′,6-diamino-2-phenylindole solution, and covering it with a glass slide. The colocalization of PANoptosis-related proteins was then examined using LSCM.

## Measurement of cytokine levels

Peripheral blood specimens (1 ml per mouse) were collected via retroorbital venipuncture into sodium heparin anticoagulant tubes (BD Vacutainer). Cellular components were separated through centrifugation (2,500*g*, 25 min, 4 °C) using a Beckman Coulter Allegra X-15R centrifuge. Plasma-free fractions from experimental cohorts were subjected to multiplex cytokine analysis following standardized protocols. Commercially available ELISA kits (MyBioSource) were employed for quantitative measurement of pro-inflammatory mediators, including IL-1α, IL-1β, TNF-α, HMGB1, IL-33, IL-18, and LDH. Analytical procedures were conducted with strict adherence to the manufacturer's specifications for optical density (OD) measurements at 450-nm wavelength.

## Transmission electron microscopy

Cellular specimens from treatment/control cohorts ($\geq 1 \times 10^7$ cells) underwent standardized processing: (a) initial stabilization: triple PBS rinsing cycles (300*g*, 5 min) to clear cellular debris; (b) chemical fixation: primary immersion–fixation in 4% glutaraldehyde (4 °C, 1 h), PBS buffer exchanges (3×). The ultrastructural processing workflow included ethanol gradient treatment (50% to 100%), infiltration with EPON-812 epoxy resin (37 °C overnight to 60 °C polymerization), and ultramicrotomy-derived 70-nm sections (Leica UC7). Electron microscopy analysis was performed using a double-contrast staining protocol, involving uranyl acetate (5% in methanol, 15 min) and lead citrate counterstain (Reynolds' method, 5 min). Ultrastructural analysis of PCD modalities was conducted using a JEOL JEM-1400FL TEM operated at 120 kV.

## CCK-8 assay

The experiment was conducted in strict adherence to the guidelines outlined in the CCK-8 test kit (MCE, New Jersey, USA). In brief, cells were harvested and rinsed with PBS. The cells were seeded into 96-well plates at a density of at least $2 \times 10^5$ cells per well, with 3 replicate wells per experimental group (splenic $CD4^+$ T lymphocytes stimulated by Con A), and incubated in a cell culture incubator for 24 h. Afterward, 10 μl of CCK-8 detection reagent was added to each well and incubated at a constant temperature for 2 to 4 h. Finally, the color change in each well was measured, and the OD value (450 nm) was determined using an ELISA plate reader.

## Isolation of murine PBMCs

Mononuclear cell populations were isolated by density gradient centrifugation (TBD Sciences Lymphoprep, China) following standardized procedures. The main operational procedures were as follows: (a) specimen acquisition: retroorbital blood collection (1 ml per mouse) into heparinized vacutainers and immediate dilution in chilled EDTA-supplemented PBS (1:2 v/v); (b) density gradient processing: the blood–PBS suspension was layered over Lymphoprep medium and centrifuged at 450*g*, 30 min, with the brake disengaged; (c) mononuclear cell isolation: the buffy coat layer (mononuclear cell interface) was aspirated, washed 3 times with PBS (300*g*, 10 min per wash), and resuspended in complete RPMI 1640 medium.

## Flow cytometric analysis

Surface marker staining was performed using fluorochrome-conjugated antibodies: anti-mouse CD3 (clone 17A2, BV510, catalog no. 100234, BioLegend, San Diego, CA), anti-human CD3 (clone OKT3, BV510, catalog no. 317332, BioLegend, San Diego, CA), anti-mouse CD4 [clone GK1.5, fluorescein isothiocyanate (FITC), catalog no. 100510, BioLegend, San Diego, CA], anti-human CD4 (clone SK3, FITC, catalog no. 344604, BioLegend, San Diego, CA), and anti-mouse CD25 [clone PC61, phycoerythrin (PE), catalog no. 102007, BioLegend, San Diego, CA]. Intracellular flow cytometry staining was performed using fluorochrome-conjugated antibodies: anti-mouse IFN-γ (clone W18272D, PE, catalog no. 163504, BioLegend, San Diego, CA), anti-mouse IL-4 [clone 11B11, allophycocyanin (APC), catalog no. 504106, BioLegend, San Diego, CA], anti-mouse IL-17A (clone TC11-18H10.1, PE, catalog no. 506904, BioLegend, San Diego, CA), and anti-mouse Foxp3

(clone FJK-16s, PE-Cyanine7, catalog no. 25-5773-82, Thermo Fisher, USA). Staining workflow included the following steps: (a) Cell preparation: mouse spleen lymphocyte suspension or PBMC aliquots ($3 \times 10^5$ cells per tube, $n = 3$ replicates) were washed 3 times with PBS and centrifugated (300*g*, 5 min, 4 °C), followed by resuspension in 100 μl of PBS; intracellular cytokine marker detection necessitates a 6-h stimulation with the Cell Activation Cocktail, which includes brefeldin A (catalog no. 423303, BioLegend, San Diego, CA). (b) Antibody labeling: 1 μl of anti-CD3/CD4/CD25 monoclonal antibodies were sequentially added, followed by 30-min incubation in the dark. Cells were then washed (3× PBS; 300*g*, 5 min). (c) Fixation and permeabilization: follow the instructions of the eBioscience Foxp3/Transcription Factor Staining Buffer Set (catalog no. 00-5523-00, Thermo Fisher, USA) for the relevant operations. (d) Intracellular staining: 1.5 μl of anti-IFN-γ/IL-4/IL-17A/Foxp3 monoclonal antibodies was sequentially added, followed by 30-min incubation in the dark. Cells were then washed (3× PBS; 300*g*, 5 min). (e) Acquisition: cells were fixed with 200 μl of 1% PFA for 15 min. Flow cytometric analysis was performed using a BD FACSCalibur system, with an acquisition of 10,000 events per sample.

## H&E staining

(a) Tissue processing: post-dissection murine organs (lung, heart, liver, and kidney) underwent immersion–fixation in 4% PFA at 4 °C for 24 h before paraffin embedding. Serial sections (4 to 5 μm) were prepared after standard xylene-based dewaxing protocols. (b) Histological analysis: tissue morphology was evaluated using H&E staining. High-resolution brightfield imaging was conducted using Nikon Eclipse microscopy systems (Japan) with standardized optical parameters. (c) Quantitative scoring: blinded pathological assessments were performed by 2 certified histopathologists. A semiquantitative analysis was performed using a 4-tier grading system: 0 (no pathology) to 3 (severe damage). Three representative fields per specimen were systematically evaluated, with comprehensive scoring criteria detailed in Table S2.

## TMT-based quantitative proteomics

Using Jurkat T cells and transfected lentivirus samples, 4 groups were established: standard controls, normal LPS, KD, and KD-LPS, each with 3 replicates. This portion of the study was carried out in collaboration with Novogene Co. Ltd. (Beijing, China). Detailed information on the tandem mass tagging (TMT) quantitative proteomic detection experimental procedure is provided in Supplementary Material 1.

## Dose–response experiment of SN-011

Jurkat T cells or mouse splenic $CD4^+$ T lymphocytes were seeded into 6-well plates at a density of $2 \times 10^6$ cells per well, with each well containing 1 ml of cell medium. The cells were then cultured at 37 °C in a 5% $CO_2$ atmosphere for 24 h. Different concentrations of SN-011 (0, 1, 5, 10, and 20 μM; MCE, New Jersey, USA) were prepared and incubated with the cells for 2 h. Subsequently, except for the control group, other groups with various concentrations of SN-011 were exposed to 500 ng/ml of LPS and cultured for an additional 24 h before harvesting the cells for further analysis. Based on prior literature, reagent instructions, and preliminary pre-experimental data, the in vivo administration method and dosage of SN-011 were established [55]. Ultimately, a concentration of 5 mg/kg was selected and administered to the mice via intraperitoneal injection.

## Polysome profiling

Ribosome collisions in $CD4^+$ T lymphocytes during sepsis were evaluated with polysome profiling by NeoRibo Biotechnology Co. Ltd. (Hangzhou, China). A Biocomp automatic density gradient preparation and ribosome sorting integrated machine was employed for sucrose density gradient separation of polyribosomes to map their distribution. The polysome profiling procedure included sample collection, cell lysis, hyperionization, component separation, RNA extraction, component analysis, and other related processes.

## Co-IP assay

Co-IP was performed using the classic protein A/G Co-IP kit (Biolinkedin, Shanghai, China). Briefly, the cell samples were collected, and specific antibodies targeting the desired protein were selected and combined with the proteins in the cell lysate. Protein A/G agarose beads were introduced and left to incubate at a low temperature overnight, followed by multiple washes with washing buffer. The antibody–protein complex was eluted using SDS sample loading buffer. The target protein and its binding partner were identified using WB analysis.

## Clinical sepsis and control patients

Individuals admitted to the Emergency Department of the Fourth Medical Center of the Chinese PLA General Hospital within a specified timeframe were chosen, and suitable candidates were enrolled. To ensure alignment with the treatment regimen for septic patients at the hospital and the requirements of future fundamental trials, a total of 10 septic patients and 10 nonseptic patients from the same period were ultimately selected. This clinical trial was approved by the Medical Ethics Committee of the Fourth Medical Center of the Chinese PLA General Hospital (approval no. 2023KY135-HS001), Beijing, China. The detailed inclusion and exclusion criteria were presented in Supplementary Material 2.

## Isolation of $CD4^+$ T lymphocytes from human peripheral blood

Approximately 6 to 8 ml of peripheral blood were obtained from each participant using a single-use negative pressure collection device, followed by the addition of half the volume of PBS to the blood sample for dilution (PBS:peripheral blood = 1:2 ratio). Subsequently, an equal amount of human peripheral blood lymphocyte isolation solution was placed in a 50-ml centrifuge tube, and the diluted peripheral blood was slowly layered on the separation medium (diluted peripheral blood:isolation solution = 1:1 ratio). The tube was centrifuged at 20 °C, 650*g* for 30 min, yielding a buffy coat of human lymphocytes at the top of the tube post-centrifugation. The lymphocytes were carefully extracted and washed twice with PBS at 1,500 rpm for 5 min each time, and $CD4^+$ T lymphocytes were isolated as per the manufacturer's instructions for anti-human $CD4^+$ T lymphocyte immunomagnetic beads (Miltenyi Biotec, Bergisch Gladbach, Germany). Finally, the cells were washed twice in PBS at 1,500 rpm (5 min per wash), and cell counts were recorded for documentation.

## Hoechst 33258 analysis

Cellular aliquots from experimental cohorts were processed through sequential centrifugation steps in flow cytometry-compatible tubes. Viability was validated, ensuring $>2 \times 10^6$

cells per experimental condition. The fixation and staining procedure comprised the following steps: (a) buffer exchange: cells were washed thrice with PBS (300*g*, 5 min each); (b) cross-linking: cells were fixed with 4% PFA (500 μl) for 1 h at ambient temperature; (c) permeabilization: cells were washed 3 times with PBS; (d) nuclear staining: cells were incubated with 3 ml of Hoechst 33258 nucleic acid staining solution (10 μg/ml; APExBIO) for 5 min at room temperature. Post-staining cells were washed with PBS before microscopic slide preparation. Apoptotic profiling was determined through fluorescence microscopy (Nikon Eclipse Ti2) with a λex/λem configuration of 352/461 nm.

### Statistical analysis

Statistical analyses were performed using IBM SPSS Statistics 24 and GraphPad Prism 8 software. Continuous variables were presented as means with standard error of the mean (SEM), while categorical or ordinal data were reported as counts and percentages, as appropriate. To compare 2 groups, an unpaired Student's *t* test was used, and for comparisons involving more than 3 groups, 1-way or 2-way analysis of variance (ANOVA) was applied. Flow cytometry data were processed with FlowJo version 10.0 software, while WB results were analyzed using ImageJ software. Survival curves for animals were generated with GraphPad Prism 8, and survival differences were assessed using the log-rank test. A $P$ value less than 0.05 was considered statistically significant. The experimental design followed a strict replication protocol, with each group replicated at least 3 times.

## Ethical Approval

All animal procedures were carried out in compliance with the International Guidelines for the Care and Use of Laboratory Animals and received approval from the Animal Ethics Committee of the Chinese PLA General Hospital (approval no. 2024-X20-40), Beijing, China. The clinical trial of this study was approved by the Medical Ethics Committee of Fourth Medical Center of the Chinese PLA General Hospital (approval no. 2023KY135-HS001), Beijing, China.

## Acknowledgments

We would like to sincerely acknowledge all the patients for their understanding and support of this study. We thank the Home for Researchers editorial team (www.home-for-researchers.com) for language editing services.

**Funding:** This work was supported by grants from the National Natural Science Foundation of China (nos. 82130062, 82241062, and 82372158) and the Beijing Natural Science Foundation (no. 7244296).

**Author contributions:** Y.Y., X.D., R.Y., and Z.L. designed the experiments. P.Z., J.L. and P.H. performed the experiments. P.Z. wrote the manuscript. Y.W., L.Z., X.Y., J.Y., Z.F., Y.X., N.C., and N.D. analyzed data and supervised the study. R.Y. and Y.Y. revised the manuscript. All authors read and approved the final manuscript.

**Competing interests:** The authors declare that they have no competing interests.

## Data Availability

The data and material will be available from the corresponding authors on reasonable request.

## Supplementary Materials

Supplementary Text
Tables S1 to S3
Figs. S1 to S7

## References

1. Meyer NJ, Prescott HC. Sepsis and septic shock. *N Engl J Med*. 2024;391(10141):2133–2146.
2. Rudd KE, Johnson SC, Agesa KM, Shackelford KA, Tsoi D, Kievlan DR, Colombara DV, Ikuta KS, Kissoon N, Finfer S, et al. Global, regional, and national sepsis incidence and mortality, 1990-2017: Analysis for the Global Burden of Disease Study. *Lancet*. 2020;395(10219):200–211.
3. Yao RQ, Zhao PY, Li ZX, Liu YY, Zheng LY, Duan Y, Wang L, Yang R-L, Kang H-J, Hao J-W, et al. Single-cell transcriptome profiling of sepsis identifies HLA-DR$^{low}$S100A$^{high}$ monocytes with immunosuppressive function. *Mil Med Res*. 2023;10(1):27.
4. Du X, Dong Z, Yan Y, Gong Y, Yuan M, Ma C, Xu L, Qu Y, Qu M, Pan P, et al. Immunomodulatory nanozymes eradicate intracellular infections and rescue immunoparalysis for treating multidrug-resistant bacterial sepsis. *Exploration*. 2025;20250127.
5. Zhang SY, Xu QP, Shi LN, Li SW, Wang WH, Wang QQ, Lu L-X, Xiao H, Wang J-H, Li F-Y, et al. Soluble CD4 effectively prevents excessive TLR activation of resident macrophages in the onset of sepsis. *Signal Transduct Target Ther*. 2023;8(1):236.
6. Xu L, Wei C, Chen Y, Wu Y, Shou X, Chen W, Lu D, Sun H, Li W, Yu B, et al. IL-33 induces thymic involution-associated naive T cell aging and impairs host control of severe infection. *Nat Commun*. 2022;13(1):6881.
7. Zhang H, Wu T, Ren C, Dong N, Wu Y, Yao Y. p53 promotes the expansion of regulatory T cells via DNMT3a- and TET2-mediated Foxp3 expression in sepsis. *Burns Trauma*. 2023;11:Article tkad021.
8. Christgen S, Tweedell RE, Kanneganti TD. Programming inflammatory cell death for therapy. *Pharmacol Ther*. 2022;232:108010.
9. Chen Y, Li X, Yang M, Liu SB. Research progress on morphology and mechanism of programmed cell death. *Cell Death Dis*. 2024;15(5):327.
10. Yang J, Chen N, Zhao P, Yang X, Li Y, Fu Z, Yan Y, Dong N, Li S, Yao R, et al. Diminished expression of GLS in CD4$^{+}$ T cells serves as a prognostic indicator associated with cuproptosis in septic patients. *Shock*. 2024;62(1):51–62.
11. Zhao PY, Yao RQ, Zheng LY, Wu Y, Li YX, Dong N, Li J-Y, Du X-H, Yao Y-M. Nuclear fragile X mental retardation-interacting protein 1-mediated ribophagy protects T lymphocytes against apoptosis in sepsis. *Burns Trauma*. 2023;11:tkac055.
12. Jin C, Zhang DP, Lin Z, Lin YZ, Shi YF, Dong XY, Jin M-Q, Song F-Q, Du S-T, Feng Y-Z, et al. Piezo1-mediated ferroptosis delays wound healing in aging mice by regulating the transcriptional activity of SLC7A11 through activating transcription factor 3. *Research*. 2025;8:0718.
13. Wang LX, Ren C, Yao RQ, Luo YN, Yin Y, Wu Y, et al. Sestrin2 protects against lethal sepsis by suppressing the pyroptosis of dendritic cells. *Cell Mol Life Sci*. 2021;78(24):8209–8227.
14. Wang Y, Weng L, Wu X, Du B. The role of programmed cell death in organ dysfunction induced by opportunistic pathogens. *Crit Care*. 2025;29(1):43.

15. Malireddi RKS, Kesavardhana S, Kanneganti TD. ZBP1 and TAK1: Master regulators of NLRP3 inflammasome/pyroptosis, apoptosis, and necroptosis (PAN-optosis). *Front Cell Infect Microbiol*. 2019;9:406.
16. Zhao PY, Yao RQ, Du XH, Yao YM. Research progress on the role of PANoptosis in human diseases. *Zhonghua Yi Xue Za Zhi*. 2022;102:2549–2554.
17. Wang WD, Sun ZJ. Evoking pyroptosis with nanomaterials for cancer immunotherapy: Current boom and novel outlook. *Nano TransMed*. 2022;1(1):Article e9130001.
18. Qi H, Li X, Ma J, Sun J, Liu Y, Wang X, Fan K, Shu C, Wang C. Fullerenols hijack lysosomes to disrupt inter-organellar cross-talk and block autophagy pre-activated by mTOR inhibitors for cancer cell PANoptosis. *Sci Bull*. 2025;70(8):1275–1294.
19. Zhong L, Guo X, Deng L, Wang X, He H, Wu N, Tang R, Chen L, Chen Y, Li P. Dopant-regulated piezocatalysts evoke sonopiezoelectric and enzymatic PANoptosis for synergistic cancer therapy. *Adv Sci*. 2025;12(17):Article e2500406.
20. Xu L, Ye Y, Gu W, Xu X, Chen N, Zhang L, Cai W, Hu J, Wang T, Chao H. Histone lactylation stimulated upregulation of PSMD14 alleviates neuron PANoptosis through deubiquitinating PKM2 to activate PINK1-mediated mitophagy after traumatic brain injury. *Autophagy*. 2025;21:1473–1491.
21. Shao R, Liu W, Feng Y, Guo X, Ren Z, Hou X, He B. LAMP2-FLOT2 interaction enhances autophagosome-lysosome fusion to protect the septic heart in response to ILC2. *Autophagy*. 2025;21:1888–1910.
22. Yao RQ, Ren C, Xia ZF, Yao YM. Organelle-specific autophagy in inflammatory diseases: A potential therapeutic target underlying the quality control of multiple organelles. *Autophagy*. 2021;17:385–401.
23. Xu B, Ye X, Sun K, Chen L, Wen Z, Lan Q, Chen J, Chen M, Shen M, Wang S, et al. IRAP drives ribosomal degradation to refuel energy for platelet activation during septic thrombosis. *Adv Sci*. 2025;12(13):Article e2411914.
24. Wan L, Juszkiewicz S, Blears D, et al. Translation stress and collided ribosomes are co-activators of cGAS. *Mol Cell*. 2021;81(13):2808–2822.e10.
25. Zhao PY, Yao RQ, Zhang ZC, Zhu SY, Li YX, Ren C, Du X-H, Yao Y-M. Eukaryotic ribosome quality control system: A potential therapeutic target for human diseases. *Int J Biol Sci*. 2022;18(6):2497–2514.
26. Kraft C, Deplazes A, Sohrmann M, Peter M. Mature ribosomes are selectively degraded upon starvation by an autophagy pathway requiring the Ubp3p/Bre5p ubiquitin protease. *Nat Cell Biol*. 2008;10(5):602–610.
27. Wyant GA, Abu-Remaileh M, Frenkel EM, Laqtom NN, Dharamdasani V, Lewis CA, Chan SH, Heinze I, Ori A, Sabatini DM, et al. NUFIP1 is a ribosome receptor for starvation-induced ribophagy. *Science*. 2018;360(6309):751–758.
28. Sundaram B, Pandian N, Mall R, Wang Y, Sarkar R, Kim HJ, Karki R, Janke LJ, Vogel P, Kanneganti T-D. NLRP12-PANoptosome activates PANoptosis and pathology in response to heme and PAMPs. *Cell*. 2023;186(13):2783–2801.e20.
29. Karki R, Sharma BR, Tuladhar S, Williams EP, Zalduondo L, Samir P, Zheng M, Sundaram B, Banoth B, RKS M. Synergism of TNF-α and IFN-γ triggers inflammatory cell death, tissue damage, and mortality in SARS-CoV-2 infection and cytokine shock syndromes. *Cell*. 2021;184(1):149–168.e17.
30. Zhou R, Ying J, Qiu X, Yu L, Yue Y, Liu Q, Shi J, Li X, Qu Y, Mu D. A new cell death program regulated by Toll-like receptor 9 through p38 mitogen-activated protein kinase signaling pathway in a neonatal rat model with sepsis associated encephalopathy. *Chin Med J*. 2022;135(12):1474–1485.
31. Zhou X, Yu X, Wan C, Li F, Wang Y, Zhang K, Feng L, Wen A, Deng J, Li S, et al. NINJ1 regulates platelet activation and PANoptosis in septic disseminated intravascular coagulation. *Int J Mol Sci*. 2023;24(4):4168.
32. Wang Y, Shi Y, Shao Y, Lu X, Zhang H, Miao C. S100A8/A9$^{hi}$ neutrophils induce mitochondrial dysfunction and PANoptosis in endothelial cells via mitochondrial complex I deficiency during sepsis. *Cell Death Dis*. 2024;15(6):462.
33. Wang Y, Fu X, Shang Z, Qiao Y, Liu Y, Zhou L, Liu D. In vivo and in vitro study on the regulatory mechanism of XiaoChaiHu decoction on PANoptosis in sepsis-induced cardiomyopathy. *J Ethnopharmacol*. 2025;336:118740.
34. He YQ, Deng JL, Zhou CC, Jiang SG, Zhang F, Tao X, Chen W-S. Ursodeoxycholic acid alleviates sepsis-induced lung injury by blocking PANoptosis via STING pathway. *Int Immunopharmacol*. 2023;125(Pt B):111161.
35. Luo S, Wang L, Xiao Y, Cao C, Liu Q, Zhou Y. Single-cell RNA-sequencing integration analysis revealed immune cell heterogeneity in five human autoimmune diseases. *BIO Integration*. 2023;4(4):145–159.
36. Wei S, Wu L, Xiang Z, Yang X, Pei D, Jiang L, Du Z. EIF2AK2 protein targeted activation of AIM2-mediated PANoptosis promotes sepsis-induced acute kidney injury. *Ren Fail*. 2024;46(2):2403649.
37. Shi FL, Li Q, Xu R, Yuan LS, Chen Y, Shi ZJ, Li Y-P, Zhou Z-Y, Xu L-H, Zha Q-B, et al. Blocking reverse electron transfer-mediated mitochondrial DNA oxidation rescues cells from PANoptosis. *Acta Pharmacol Sin*. 2024;45:594–608.
38. Yang Z, Kao X, Zhang L, Huang N, Chen J, He M. Exploring the anti-PANoptosis mechanism of Dachaihu decoction against sepsis-induced acute lung injury: Network pharmacology, bioinformatics, and experimental validation. *Drug Des Devel Ther*. 2025;19:349–368.
39. Chen S, Wu GD, Li T, Jiang J, Zhong Y, Sun D, Qian F, Huang LS. Targeting GSDMD JX06 inhibits PANoptosis and multiple organ injury. *Biochem Pharmacol*. 2025;233:Article 116765.
40. Chen Y, Hu J, Zhao P, Fang J, Kuang Y, Liu Z, Dong S, Yao W, Ding Y, Wang X, et al. Rpl12 is a conserved ribophagy receptor. *Nat Cell Biol*. 2025;27(3):477–492.
41. Bai M, Lei J, Li F, Wang X, Fu H, Yan Z, et al. Short-chain chlorinated paraffins may induce ovarian damage in mice via AIM2- and NLRP12-PANoptosome. *Environ Sci Technol*. 2025;59(1):163–176.
42. Kesavardhana S, Malireddi RKS, Burton AR, Porter SN, Vogel P, Pruett-Miller SM, Kanneganti T-D. The Zα2 domain of ZBP1 is a molecular switch regulating influenza-induced PANoptosis and perinatal lethality during development. *J Biol Chem*. 2020;295(24):8325–8330.
43. Luo Z, Mei J, Wang X, Wang R, He Z, Geffen Y, Sun X, Zhang X, Xu J, Wan R, et al. Voluntary exercise sensitizes cancer immunotherapy via the collagen inhibition-orchestrated inflammatory tumor immune microenvironment. *Cell Rep*. 2024;43(9):114697.
44. Malireddi RKS, Gurung P, Kesavardhana S, Samir P, Burton A, Mummareddy H, Vogel P, Pelletier S, Burgula S, Kanneganti T-D, et al. Innate immune priming in the absence

of TAK1 drives RIPK1 kinase activity-independent pyroptosis, apoptosis, necroptosis, and inflammatory disease. *J Exp Med*. 2020;217(3):Article jem20191644.
45. Mei J, Luo Z, Cai Y, Wan R, Qian Z, Chu J, Sun Y, Shi Y, Jiang Y, Zhang Y, et al. Altered atlas of exercise-responsive microRNAs revealing miR-29a-3p attacks armored and cold tumors and boosts anti-B7-H3 therapy. *Research*. 2025;8:0590.
46. Yang W, Xu Y, Liu S, Gao L, Li S, Xie X, Zhang Q, Habib O, Chen R, Sun X, et al. Mebendazole induces ZBP-1 mediated PANoptosis of acute myeloid leukemia cells by targeting TUBA1A and exerts antileukemia effect. *J Adv Res*. 2025;1232(25):00111-0.
47. Bi X, Li M, Guo Y, Hu M, Chen Y, Lian N, Chen S, Li M, Gu H, Chen X, et al. ZBP1-mediated PANoptosis is a crucial lethal form in diverse keratinocyte death modalities in UVB-induced skin injury. *Cell Death Dis*. 2025;16(1):44.
48. Lou J, Mao Y, Jiang W, Shen H, Fan Y, Yu Q, Zhou C, Wei Z, Zhou K, Jin M, et al. TRIM56 modulates YBX1 degradation to ameliorate ZBP1-mediated neuronal PANoptosis in spinal cord injury. *Adv Sci*. 2024;11(42):Article e2407132.
49. Gong T, Wang QD, Loughran PA, Li YH, Scott MJ, Billiar TR, Liu Y-T, Fan J. Mechanism of lactic acidemia-promoted pulmonary endothelial cells death in sepsis: Role for CIRP-ZBP1-PANoptosis pathway. *Mil Med Res*. 2024;11(1):71.
50. Chen W, Kanneganti TD. Heartbreakers: Innate sensors ZBP1 and cGAS linked to cardiotoxicity. *Cell Res*. 2023;33(12): 902–903.
51. Zhang Z, Zhang C. Regulation of cGAS-STING signaling and its diversity of cellular outcomes. *Nat Rev Immunol*. 2025;25(6):425–444.
52. Han Y, Qiu L, Wu H, Song Z, Ke P, Wu X. Focus on the cGAS-STING signaling pathway in sepsis and its inflammatory regulatory effects. *J Inflamm Res*. 2024;17:3629–3639.
53. Zhao C, Hu X, Bao L, Wu K, Zhao Y, Xiang K, et al. Gut dysbiosis induces the development of mastitis through a reduction in host anti-inflammatory enzyme activity by endotoxemia. *Microbiome*. 2022;10:205.
54. Liu N, Pang X, Zhang H, Ji P. The cGAS-STING pathway in bacterial infection and bacterial immunity. *Front Immunol*. 2022;12:Article 814709.
55. Hong Z, Mei J, Li C, Bai G, Maimaiti M, Hu H, Yu W, Sun L, Zhang L, Cheng D, et al. STING inhibitors target the cyclic dinucleotide binding pocket. *Proc Natl Acad Sci USA*. 2021;118:Article e2105465118.
56. Maimaiti M, Li C, Cheng M, Zhong Z, Hu J, Yang L, Zhang L, Hong Z, Song J, Pan M, et al. Blocking cGAS-STING pathway promotes post-stroke functional recovery in an extended treatment window via facilitating remyelination. *Med*. 2024;5(6):622–644.e8.
