# Supplementary material for: NUFIP1-Mediated Ribophagy Alleviates PANoptosis of CD4+ T Lymphocytes in Sepsis via the cGAS-STING Pathway
Source: Research (Wash D C). 2025 Sep 23;8:0895. doi: 10.34133/research.0895 (PMC12454940; doi:10.34133/research.0895)
Supplement: Supplementary 1 — Supplementary Text Tables S1 to S3 Figs. S1 to S7 [file research.0895.f1.zip › Corrected Supplementary Information.pdf]

Supporting Information

Supplemental Material 1 Experimental method for TMT-based quantitative proteomics analysis.

Supplemental Material 2 Inclusion and exclusion criteria of clinical patients.

Supplemental Table 1 Specific antibodies utilized in Western blot analysis.

Supplemental Table 2 Criteria for the microscopic scoring of organ injuries.

Supplemental Table 3 TOP 20 differentially expressed proteins screened by TMT labeled quantitative proteomics sequencing.

Supplemental Figure 1 The time- and dose-response experiments of T lymphocyte PANoptosis in sepsis.

Supplemental Figure 2 The interaction between NUFIP1 and ZBP1 detected by Co-IP.

Supplemental Figure 3 Impact of conditional deletion of *NUFIP1* on PANoptosis of splenic $CD4^+$ T lymphocytes in septic mice.

Supplemental Figure 4 Impact of conditional deletion of *NUFIP1* on the immune response of $CD4^+$ T lymphocytes stimulated with LPS.

Supplemental Figure 5 TMT proteomic sequencing to explore the differentially expressed proteins of Jurkat T cells in the standard control and KD groups stimulated with LPS.

Supplemental Figure 6 The molecular mechanism of NUFIP1-mediated ribophagy in activating cGAS-STING pathway.

Supplemental Figure 7 Dose-response experiment of Jurkat T cells treated with SN-011 and regulatory effects of cGAS-STING signaling pathway on Jurkat T cells PANoptosis in sepsis.

Supplemental File 1 The original uncropped WB images for the Figures.

Supplemental File 2 Certificate of English Editing.

Supplementary Material 1

Experimental method for TMT-based quantitative proteomics analysis

1. Total protein extraction

The samples of Jurkat T cells were transferred into a 1.5 mL centrifuge tube and lysed with DB lysis buffer (8 M urea, 100 mM TEAB, pH 8.5), followed by 5 min of ultrasonication on ice. The lysate was centrifuged at 12000 g for 15 min at 4°C. The supernatant was reduced with 10 mM DTT for 1 h at 56°C, and subsequently alkylated with sufficient iodoacetamide for 1 h at room temperature in the dark.

2. Protein quality test

BSA standard protein solution was prepared according to the instructions of the Bradford protein quantitative kit, with a gradient concentration ranging from 0 to 0.5 g/L. BSA standard protein solutions and sample solutions with different dilution multiples were added into a 96-well plate to fill up the volume to 20 μL, respectively. Each gradient was repeated three times. The plate was quickly added to 180 μL G250 dye solution and then placed at room temperature for 5 min. The absorbance at 595 nm was detected. The standard curve was drawn with the absorbance of the standard protein solution, and the protein concentration of the sample was calculated. 20 μg of the protein sample was loaded onto a 12% SDS-PAGE gel, wherein the concentrated gel was performed at 80 V for 20 min, and the separation gel was performed at 120 V for 90 min. The gel was stained with Coomassie Brilliant Blue R-250 and decolored until the bands were visualized clearly.

3. TMT labeling of peptides

Each protein sample was taken, and the volume was made up to 100 μL with DB dissolution buffer (8 M urea, 100 mM TEAB, pH 8.5). Trypsin and 100 mM TEAB buffer were added, the sample was mixed, and digested at 37°C for 4 h. Then, trypsin and $CaCl_2$ were added, and the sample was digested overnight. Formic acid was mixed with the digested sample, adjusted to a pH under 3, and centrifuged at 12000 g for 5 min at room temperature. The supernatant was slowly loaded into the C18 desalting column, washed with washing buffer (0.1% formic acid, 3% acetonitrile) three times,

and eluted with elution buffer (0.1% formic acid, 70% acetonitrile). The eluents of each sample were collected and lyophilized. 100 μL of 0.1 M TEAB buffer was added to reconstitute, and 4 μL of acetonitrile-dissolved TMT labeling reagent was added. The sample was mixed with shaking for 2 h at room temperature. Thereafter, the reaction was stopped by adding 8% ammonia. All labeling samples were mixed with an equal volume, desalted, and lyophilized. Note: for multiple labelled groups, a standard reference was created by pooling an equal quantity of each sample.

4. Separation of fractions

Mobile phase A (2% acetonitrile, adjusted pH to 10.0 using ammonium hydroxide) and B (98% acetonitrile) were used to develop a gradient elution. The lyophilized powder was dissolved in solution A and centrifuged at 12000 g for 10 min at room temperature. The sample was fractionated using a C18 column (Waters BEH C18, 4.6 × 250 mm, 5 μm) on a Rigol L3000 HPLC system; the column oven was set to 45°C. The detail of the elution gradient was shown as follows: 3% B, 0 min; 3%-5% B, 10 min; 5%-20% B, 20 min; 20%-40% B, 18 min; 40%-50% B, 2 min; 50%-70% B, 3 min; 70%-100% B, 1 min. The eluates were monitored at UV 214 nm, collected at a rate of one tube per minute, and then combined into 10 fractions. All fractions were dried under vacuum and then reconstituted in 0.1% (v/v) formic acid (FA) in water.

5. LC-MS/MS analysis

For transition library construction, shotgun proteomics analyses were performed using an EASY-nLC^{TM} 1200 UHPLC system (Thermo Fisher, USA) coupled with a Q Exactive^{TM} series mass spectrometer (Thermo Fisher, USA) operating in the data-dependent acquisition (DDA) mode. 1 μg sample was injected into a home-made C18 Nano-Trap column (4.5 cm × 75 μm, 3 μm). Peptides were separated in a home-made analytical column (15 cm × 150 μm, 1.9 μm), using a linear gradient elution as follows: 6% B, 0 min; 6%-15% B, 2 min; 15%-40% B, 78.5 min; 40%-50% B, 2 min; 50%-55% B, 1 min; 55%-100% B, 8.5 min. The separated peptides were analyzed by Q Exactive^{TM} series mass spectrometer (Thermo Fisher, USA), with an iron source of Nanospray Flex™ (ESI), spray voltage of 2.3 kV, and ion transport capillary temperature of 320°C. Full scan ranges from *m/z* 350 to 1500 with a resolution of 60000

(at *m/z* 200), an automatic gain control (AGC) target value was 3×10^6, and a maximum ion injection time was 20 ms. The top 40 precursors of the highest abundant in the full scan were selected and fragmented by higher energy collisional dissociation (HCD) and analyzed in MS/MS, where resolution was 45000 (at *m/z* 200) for 10 plex, the AGC target value was 5×10^4 the maximum ion injection time was 86 ms, a normalized collision energy was set as 32%, an intensity threshold was 1.2×10^5, and the dynamic exclusion parameter was 20 s. The raw data of MS detection was named as ".raw".

6. Data analysis

6.1 The identification and quantitation of protein

The resulting spectra from each run were searched separately against homo_sapiens_uniprot_2021_7_15.fasta.fasta (202195 sequences) database by the search engines: Proteome Discoverer 2.2 (PD 2.2, Thermo). The searched parameters were as follows: a mass tolerance of 10 ppm for precursor ion scans and a mass tolerance of 0.02 Da for the product ion scans was used. Carbamidomethyl was specified in PD 2.2 as a fixed modification. Oxidation of methionine, acetylation of the N-terminus, and TMT of lysine were specified in PD 2.2 as variable modifications. A maximum of 2 miscleavage sites were allowed.

To improve the quality of analysis results, PD 2.2 software further filtered the retrieval results, identifying Peptide Spectrum Matches (PSMs) with a credibility of more than 99%. The identified protein contains at least 1 unique peptide. The identified PSMs and proteins were retained and performed with FDR no more than 1.0%. A t-test statistically analyzed the protein quantitation results. The proteins whose quantitation was significantly different between experimental and control groups ($p < 0.05$ and $|\log_2 FC| > 1.5$ (FC > 1.5 or FC <0.67 [foldchange, FC])) were defined as differentially expressed proteins (DEP).

6.2 The functional analysis of protein and DEP

Gene Ontology (GO) and InterPro (IPR) functional analysis were conducted using the InterProScan program against the non-redundant protein database (including Pfam, PRINTS, ProDom, SMART, ProSite, PANTHER), and the databases of COG (Clusters of Orthologous Groups) and KEGG (Kyoto Encyclopedia of Genes and Genomes) were

used to analyze the protein family and pathway. DPEs were used for Volcanic map analysis, cluster heat map analysis, and enrichment analysis of GO, IPR and KEGG. The probable protein-protein interactions were predicted using the STRING-db server (http://string.embl.de/).

References

[1] Kachuk C, Stephen K, Doucette A. Comparison of sodium dodecyl sulfate depletion techniquesfor proteome analysis by mass spectrometry. Journal of chromatography, A: Including electrophoresis and other separation methods, 2015, 1418:158-166.

[2] Wisniewski JR, Zougman A, Nagaraj N, et al. Universal sample preparation method for proteome analysis. Nature Methods, 2009, 6:359-362.

[3] Gillette MA, Satpathy S, Cao S, et al. Proteogenomic characterization reveals therapeutic vulnerabilities in lung adenocarcinoma. Cell, 2020, 82:200-225.e35.

[4] Zhang H, Liu T, Zhang Z, et al. Integrated proteogenomic characterization of human high-grade serous ovarian cancer. Cell, 2016, 166:755-765.

[5] Jones P, Binns D, Chang H Y, et al. InterProScan 5: genome-scale protein function classification. Bioinformatics, 2014, 30:1236-1240.

[6] Huang DW, Sherman BT, Lempicki RA. Bioinformatics enrichment tools: paths toward the comprehensive functional analysis of large gene lists. Nucleic Acids Research, 2009, 37:1-13.

[7] Franceschini A, Szklarczyk D, Frankild S, et al. STRING V9.1: protein-protein interaction networks, with increased coverage and integration. Nucleic Acids Research, 2012, 41:D808-815.

Supplementary Material 2

Inclusion and exclusion criteria for clinical patients

1. Inclusion criteria

1) Critically ill patients admitted to the EICU of our hospital from June 1, 2023, to December 31, 2023;

2) Meet the diagnostic criteria of Sepsis 3.0;

3) Age ≥18 years and ≤ 80 years.

2. Exclusion criteria

1) EICU hospitalization time <24 hours;

2) Repeated and multiple stays in EICU;

3) Long-term use of immunosuppressants and glucocorticoids;

4) Malignant tumor patients;

5) HIV positive;

6) Incomplete case information;

7) Pregnant or lactating women;

8) Receive chemotherapy within 4 weeks;

9) Not willing to sign the agreement.

Supplemental Table 1 Specific antibodies utilized in Western blot analysis.

Antibodies	Source	Identifier
Anti-β-actin (host: mouse)	Protintech	HRP-66009
Anti-GSDMD (host: rabbit)	CST	#39754
Anti-NLRP3 (host: rabbit)	Huaxingbio	HX23650
Anti-ASC (host: rabbit)	CST	#13833 #67824
Anti-Bax (host: mouse)	Protintech	60267-1-Ig
Anti-Blc-2 (host: mouse)	Protintech	68103-1-Ig
Anti-cleaved-Caspase-3 (host: rabbit)	CST	#9661
Anti-p-MLKL (host: rabbit)	CST	#91689 #37333
Anti-p-RIPK3 (host: rabbit)	CST	#93654 #91702
Anti-NUFIP1 (host: rabbit)	Protintech	12515-1-AP
Anti-RPL7 (host: rabbit)	Abcam	ab72550
Anti-RPL23 (host: rabbit)	Protintech	16086-1-AP
Anti-RPL26 (host: rabbit)	Protintech	17619-1-AP
Anti-LC3B (host: rabbit)	CST	#4108
Anti-p62 (host: rabbit)	CST	#5114
Anti-t-MLKL (host: mouse)	CST	#26539
Anti-t-RIPK3 (host: mouse)	Santa Cruz	SC-374639
Anti-ZBP1 (host: mouse)	AdipoGen	AG-20B-0010-C100
Anti-AIM2 (host: rabbit)	Protintech	20590-1-AP
Anti-RIPK1(host: rabbit)	CST	#3493
Anti-NLRP12 (host: rabbit)	Protintech	30998-1-AP
Anti-cGAS (host: rabbit)	Protintech	29958-1-AP
Anti-t-STING (host: rabbit)	Protintech	19851-1-AP
Anti-p-STING (host: rabbit)	CST	#19781 #72971
Anti-t-TBK1 (host: rabbit)	CST	#38066
Anti-p-TBK1 (host: rabbit)	CST	#5483
Anti-t-IRF3 (host: rabbit)	CST	#4302

Anti-p-IRF3 (host: rabbit)	CST	#29047

Abbreviations: GSDMD: Gasdermin D; NLRP3: nucleotide binding oligomerization domain-like receptor protein 3; ASC: apoptosis speck-like protein containing a caspase recruitment domain; Bax: BCL2-associated X protein; Bcl-2: B-cell lymphoma 2; p-MLKL: phosphorylated pseudo kinase mixed family kinase-like domain; p-RIPK3: phosphorylated receptor interacting protein kinase 3; NUFIP1: nuclear fragile X mental retardation-interacting protein 1; RPL7: 60S ribosomal protein L7; RPL23: 60S ribosomal protein L23; RPL26: 60S ribosomal protein L26; LC3B: microtubule-associated protein light chain 3; p62: nucleoporin 62; t-MLKL: total pseudo kinase mixed family kinase-like domain; t-RIPK3: total receptor interacting protein kinase 3; ZBP1: Z-DNA-binding protein 1; AIM2: absent in melanoma 2; RIPK1: receptor interacting protein kinase 1; NLRP12: nucleotide binding oligomerization domain-like receptor protein 12; cGAS: cyclic GMP-AMP synthase; t-STING: total stimulator of interferon genes; p-STING: phosphorylated stimulator of interferon genes; t-TBK1: total TANK-binding kinase 1; p-TBK1: phosphorylated TANK-binding kinase 1; t-IRF3: total interferon regulatory factor 3; p-IRF3: phosphorylated interferon regulatory factor 3.

Supplemental Table 2 Criteria for the microscopic scoring of organ injuries.

Organ injuries	Scoring items
**Cardiac injury**	Parenchymal congestion
	Necrosis
	Inflammation
**Lung injury**	Pulmonary edema
	Parenchymal congestion
	Alveolar hemorrhage
	Peribronchial inflammation
	Perivascular inflammation
	Interstitial inflammation
**Liver injury**	Ischemic necrosis
	Parenchymal congestion
	Hepatocellular injury
	Periportal inflammation
	Vacuolar degeneration
**Kidney injury**	Congestion
	Glomerular necrosis
	Tubular necrosis

Scores for each criterion are given as 0, none; 1, mild; 2, moderate; 3, severe. At least three microscopic areas were examined to score each specimen.

Supplemental Table 3 TOP 20 differentially expressed proteins screened by TMT-labeled quantitative proteomics sequencing.

Ranks	Upregulated expression	Down-regulated expression
1	Beta-galactosidase (fragment)	Zinc finger HIT domain
2	40S ribosomal protein SA	Hemoglobin subunit gamma (fragment)
3	Chromosomal protein HMG-17	Endoplasmic reticulum-Golgi intermediate compartment protein 1
4	cDNA FLJ00127	cAMP-dependent protein kinase type I
5	Phosphomannomutase	cDNA FLJ53848
6	Thymosin	cDNA FLJ60629
7	Twinfilin-1	Apolipoprotein A-I
8	Ubiquitin-conjugating enzyme E2D	cDNA FLJ59619
9	cDNA FLJ93365	cDNA FLJ54622
10	Sclerostin domain containing 1	Cathepsin G
11	Protrudin	cDNA FLJ35376
12	Autophagy-related protein 2	ATIII-T1
13	THRAP3 protein	Methyl sterol monooxygenase 1
14	Nuclear ribonucleoprotein A3	Bc1-2
15	G protein-regulated inducer	Serpin B8
16	Caspase-8	Glutathione peroxidase 3
17	60S ribosomal protein L23	Transmembrane protein 14C variant
18	MLKL	cDNA FLJ51412
19	Histone H1.5	Regucalcin
20	Bax	cDNA FLJ60629

Abbreviations: HMG-17: high mobility group-17; THRAP3: thyroid hormone receptor-associated protein 3; MLKL: phosphorylated pseudo kinase mixed family kinase-like; Bax: BCL2-associated X protein; ATIII-T1: antithrombin III-T1; Bcl-2: B-cell lymphoma 2.

Supplementary Figure 1

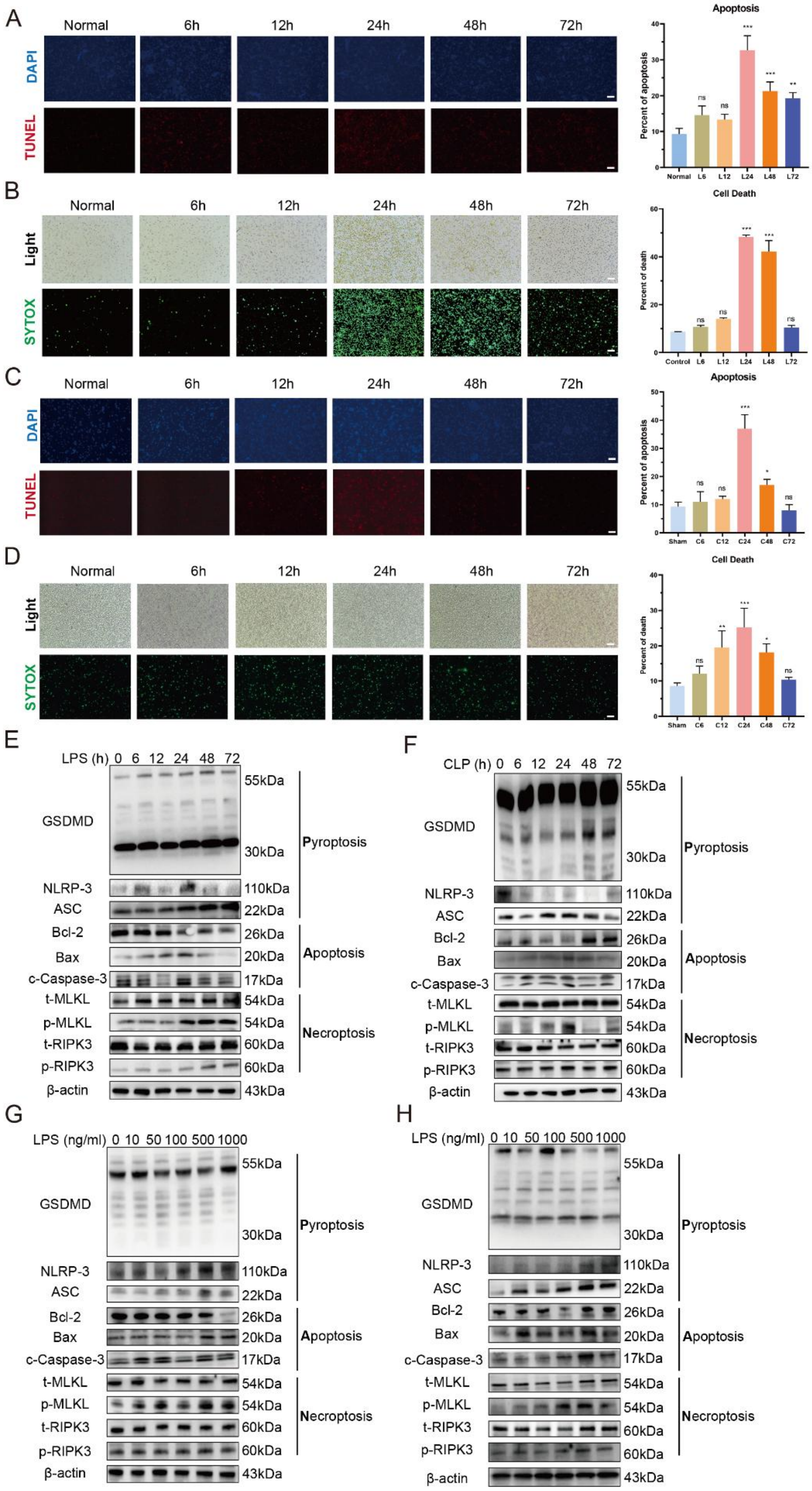

Fig. S1. The time- and dose-response experiments of CD4$^+$ T lymphocytes' PANoptosis in sepsis. (A) Apoptosis of CD4$^+$ T lymphocytes was detected by TUNEL after LPS stimulation at different time points. The scale bar represents 100 μm. n = 3 technical repetitions. (B) Necrosis of CD4$^+$ T lymphocytes was detected by SYTOX-Green after LPS stimulation at different time points. The scale bar represents 100 μm. n = 3 technical repetitions. (C) Apoptosis of CD4$^+$ T lymphocytes was detected by TUNEL at different intervals after CLP. The scale bar represents 100 μm. n = 3 technical repetitions. (D) Necrosis of CD4$^+$ T lymphocytes was detected by SYTOX-Green at different intervals after CLP. The scale bar represents 100 μm. n = 3 technical repetitions. (E) The expression of PANoptosis-related proteins in CD4$^+$ T lymphocytes was determined by WB after LPS stimulation at different time points. (F) The expression of PANoptosis-related proteins in CD4$^+$ T lymphocytes was determined by WB after different intervals after CLP. (G) The expression of PANoptosis-related proteins in Jurkat T cells was determined by WB at various LPS concentrations. (H) The expression of PANoptosis-related proteins in CD4$^+$ T lymphocytes was determined by WB at various LPS concentrations. Data were expressed as means ± SEM. An unpaired two-sided Student's t test was applied to test the statistical significance. *$P < 0.05$, **$P < 0.01$, ***$P < 0.001$.

Supplementary Figure 2

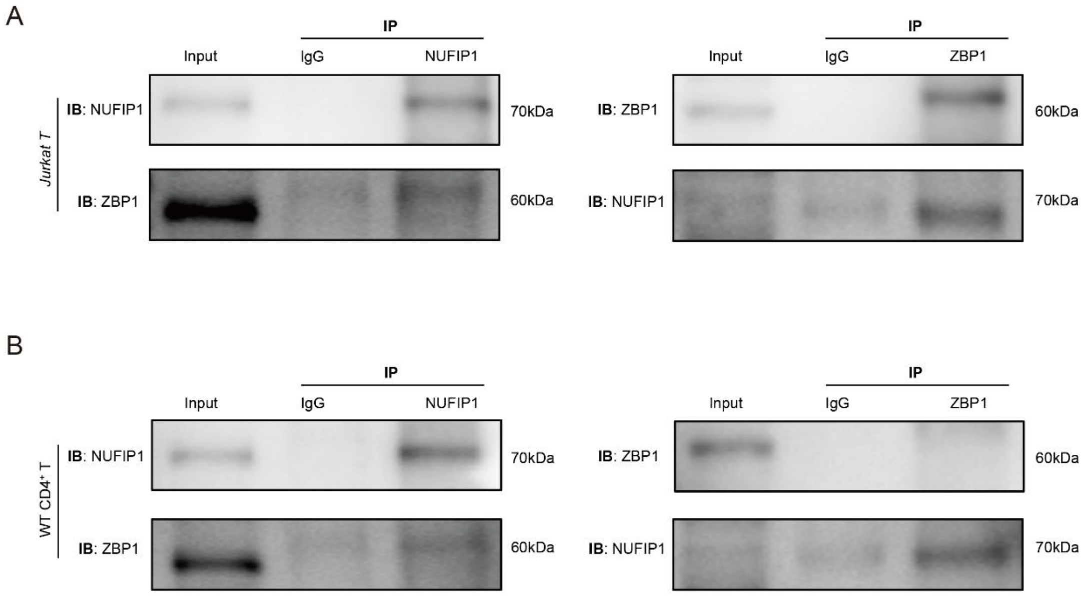

Fig. S2. The interaction between NUFIP1 and ZBP1 was detected by Co-IP. (A) The interaction between NUFIP1 and ZBP1 in Jurkat T cells was detected by Co-IP. (B) The interaction between NUFIP1 and ZBP1 in primary $CD4^{+}$ T lymphocytes isolated from mouse spleen was detected by Co-IP.

Supplementary Figure 3

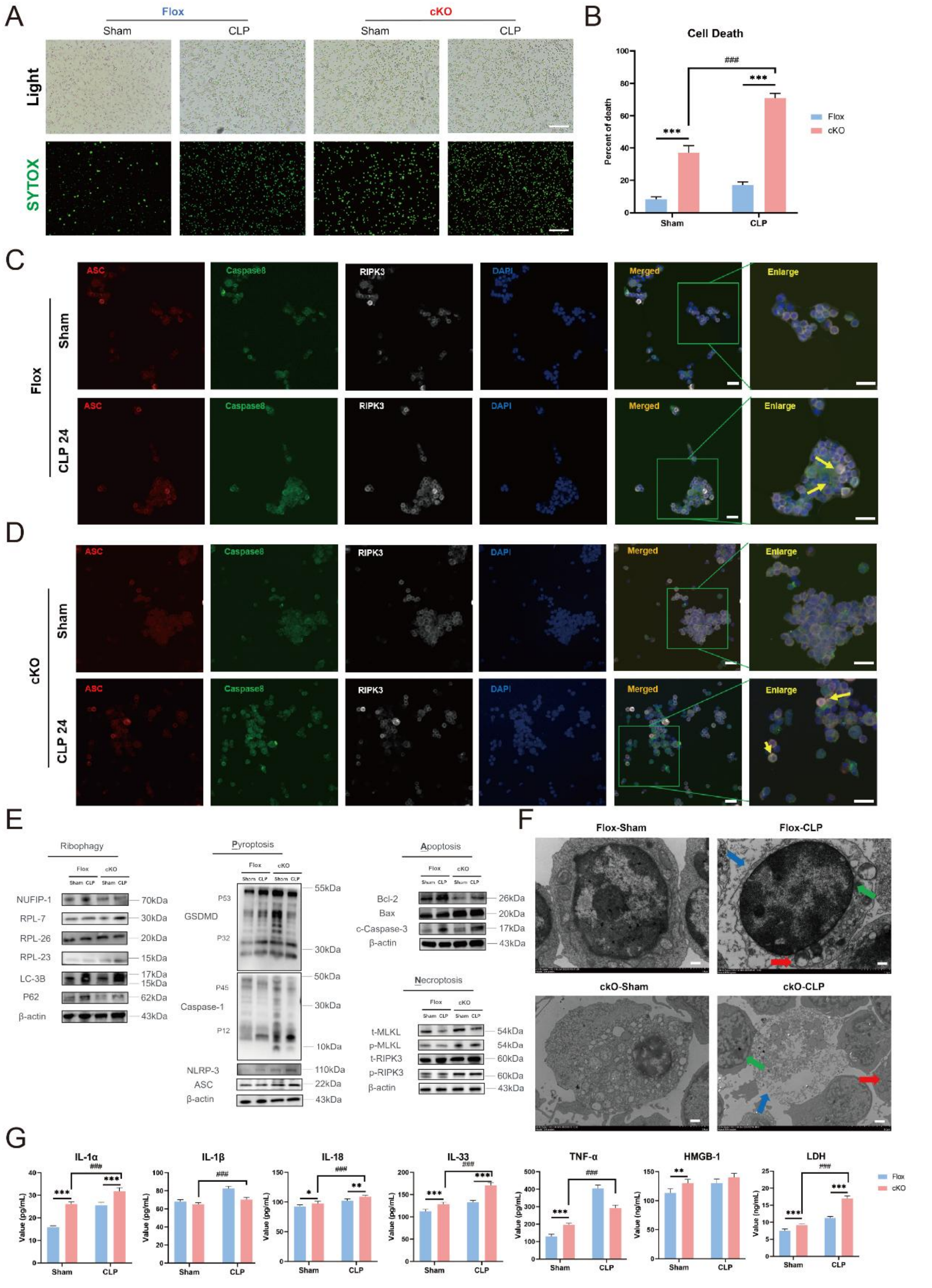

Fig. S3. Impact of conditional deletion of *NUFIP1* on PANoptosis of splenic CD4$^+$ T lymphocytes in septic mice. (A and B) The necrosis of spleen CD4$^+$ T lymphocytes in mice was detected by SYTOX-Green after CLP. The scale bar represents 100 μm. *n* =

3 technical repetitions. (C) The expression and co-localization of ASC/Caspase8/RIPK3 in splenic $CD4^+$ T lymphocytes of Flox mice were determined by LSCM after CLP operation. The yellow arrows represent PANoptosomes, and the scale bar represents 25 μm. (D) The expression and co-localization of ASC/Caspase8/RIPK3 in splenic $CD4^+$ T lymphocytes of cKO mice were determined by LSCM after CLP operation. The yellow arrows represent PANoptosomes, and the scale bar represents 25 μm. (E) The expression of PANoptosis-related proteins in splenic $CD4^+$ T lymphocytes of cKO mice by WB after CLP. (F) The morphological characteristics of PANoptosis in splenic $CD4^+$ T lymphocytes of Flox and cKO mice under TEM after CLP. The red arrow denotes the intracellular vesicles associated with pyroptosis; the green arrow signifies the nuclear condensation and chromatin shrinkage characteristic of cell apoptosis; the blue arrow indicates the loss of cell membrane integrity in necroptosis, and the scale bar represents 500 nm. (G) Serum cytokine levels in Flox and cKO groups were measured by ELISA after CLP operation. $n = 6$ technical repetitions. Data were expressed as means ± SEM. A two-way ANOVA test was applied to test the statistical significance. $^{*}P < 0.05$, $^{**}P < 0.01$, $^{***}P < 0.001$ compared with the Flox group. $^{\#\#\#}P < 0.001$ compared with the cKO-Sham group.

Supplementary Figure 4

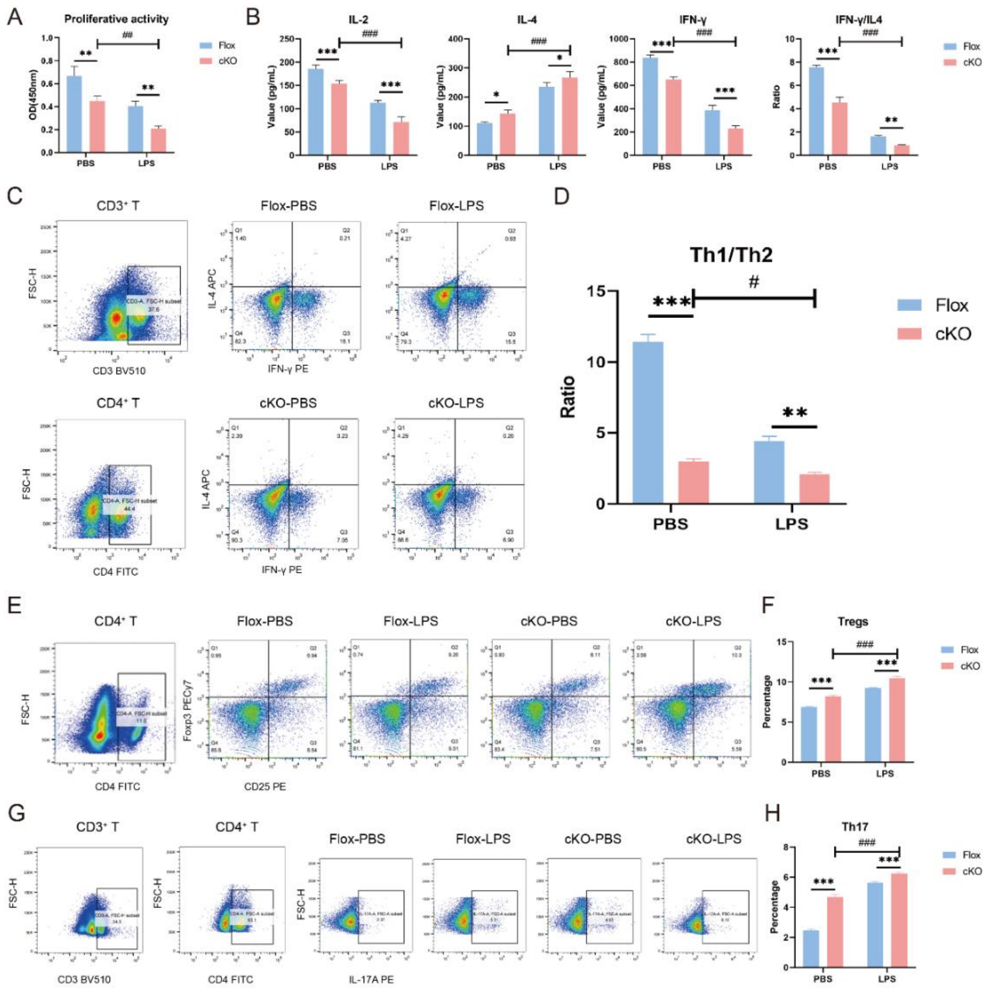

Fig. S4. Impact of conditional deletion of *NUFIP1* on the immune response of CD4$^+$ T lymphocytes stimulated with LPS. (A) The proliferative activity of splenic CD4$^+$ T cells was detected by CCK-8 after LPS stimulation. $n = 3$ technical repetitions. (B) Cytokine secretion levels of splenic CD4$^+$ T cells culture supernatant were measured by ELISA in the Flox and cKO groups. $n = 4$ technical repetitions. (C and D) The ratio of Th1/Th2 of splenic CD4$^+$ T cells was detected by flow cytometry. $n = 3$ technical repetitions. (E and F) The percentage of Tregs of splenic CD4$^+$ T cells was detected by flow cytometry. $n = 3$ technical repetitions. (G and H) The percentage of Th17 cells among splenic CD4$^+$ T cells was detected by flow cytometry. $n = 3$ technical repetitions. Data were expressed as means ± SEM. A two-way ANOVA test was applied to test the statistical significance. $^*P < 0.05$, $^{**}P < 0.01$, $^{***}P < 0.001$ compared with the Flox group. $^{\#\#}P < 0.01$, $^{\#\#\#}P < 0.001$ compared with the cKO-PBS group.

Supplementary Figure 5

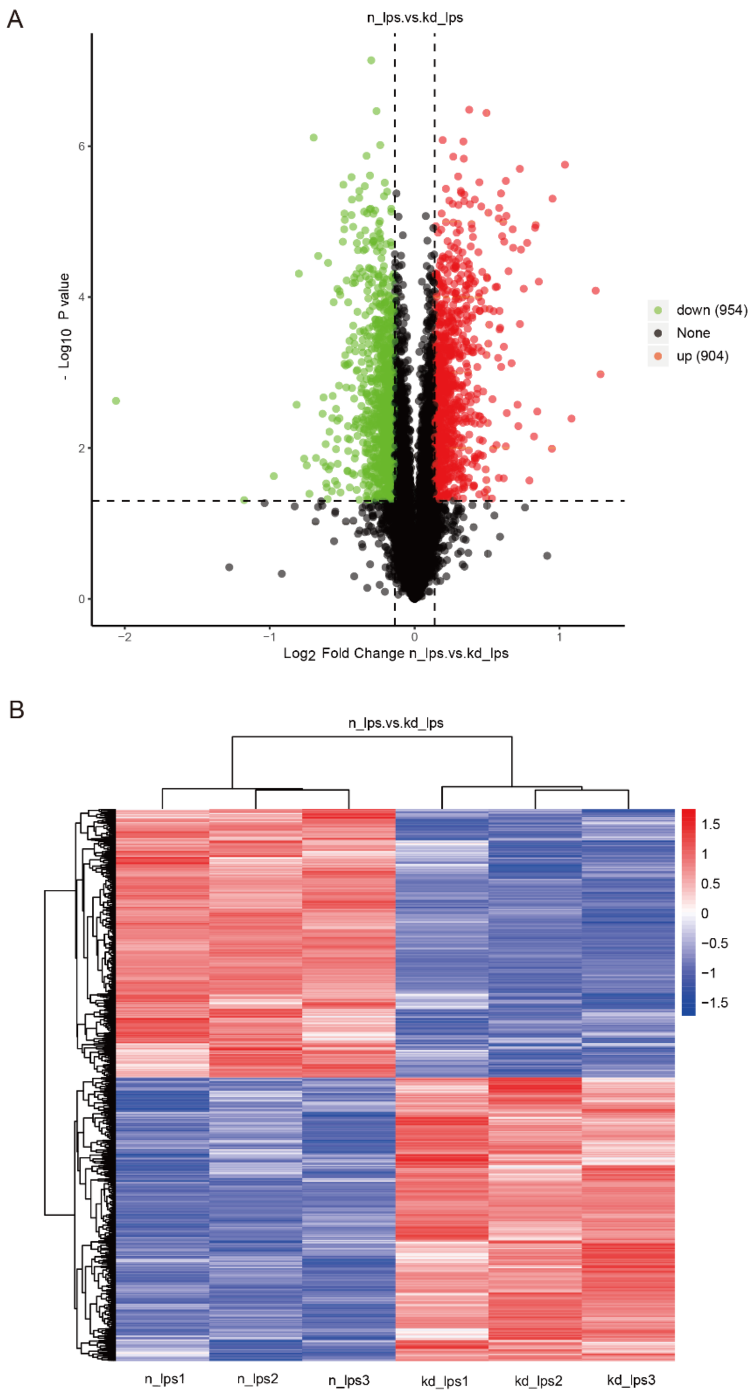

Fig. S5. TMT proteomic sequencing to explore the differentially expressed proteins of Jurkat T cells in the standard control and KD groups stimulated with LPS. (A) Differentially expressed proteins volcano maps of Jurkat T cells in the standard control and KD groups stimulated with LPS. (A) Differentially expressed proteins clustering heat maps of Jurkat T cells in Normal and KD groups stimulated by LPS.

Supplementary Figure 6

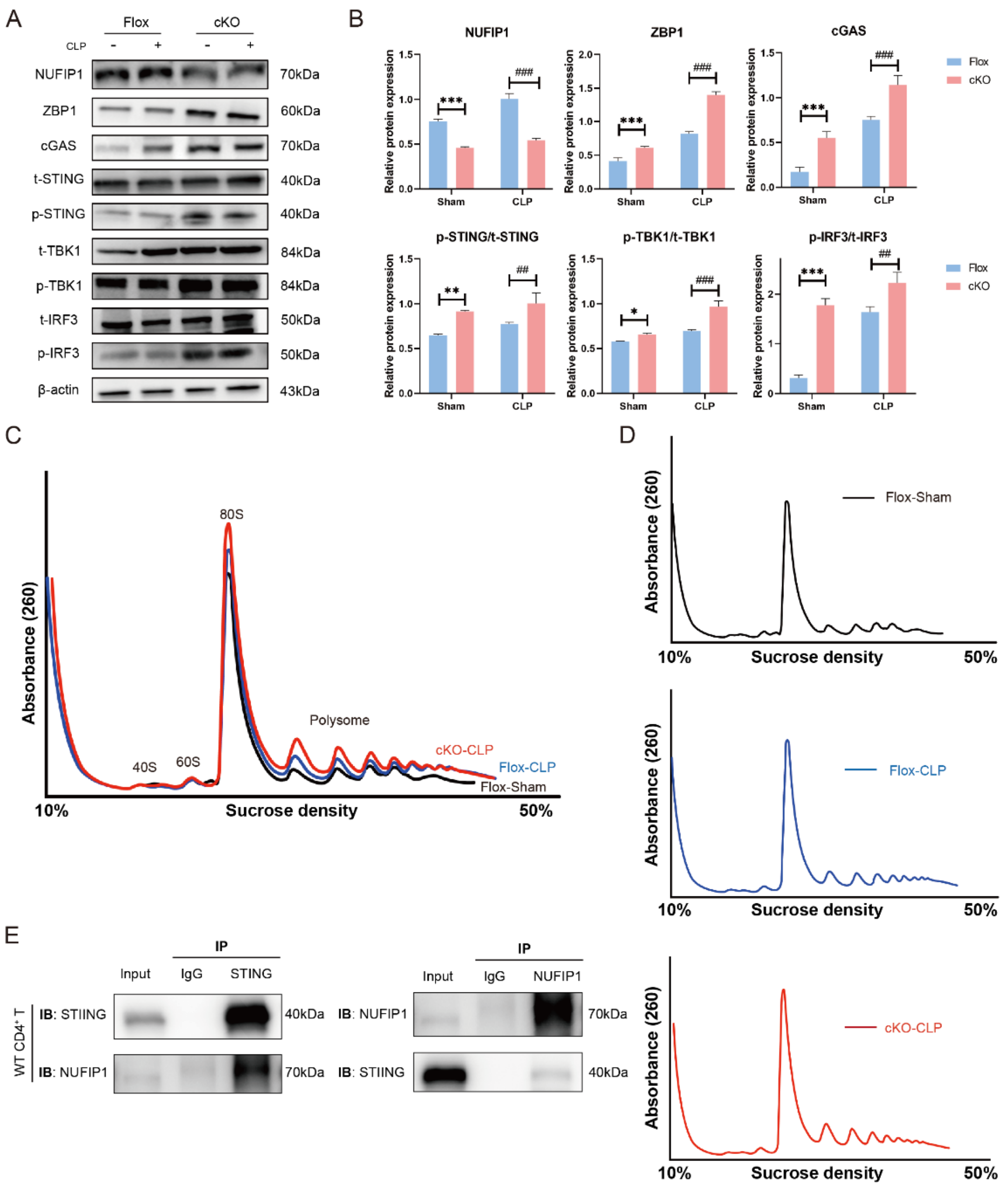

Fig. S6. The molecular mechanism of NUFIP1-mediated ribophagy in activating the cGAS-STING pathway. (A and B) The expression of cGAS-STING signaling-related proteins in splenic CD4$^+$ T cells of the Flox and cKO groups by WB after CLP operation. $n = 3$ technical repetitions. (C and D) Ribosomal collision events of CD4$^+$ T cells were assessed by polysome profiling. (E) The interaction between NUFIP1 and STING of CD4$^+$ T cells was determined by Co-IP. Data were expressed as means ± SEM. A two-way ANOVA test was applied to test the statistical significance. $^*P < 0.05$, $^{**}P < 0.01$, $^{***}P < 0.001$ compared with the Flox-Sham group; $^{\#}P < 0.05$, $^{\#\#}P < 0.01$, $^{\#\#\#}P < 0.001$ compared with the Flox-CLP group.

Supplementary Figure 7

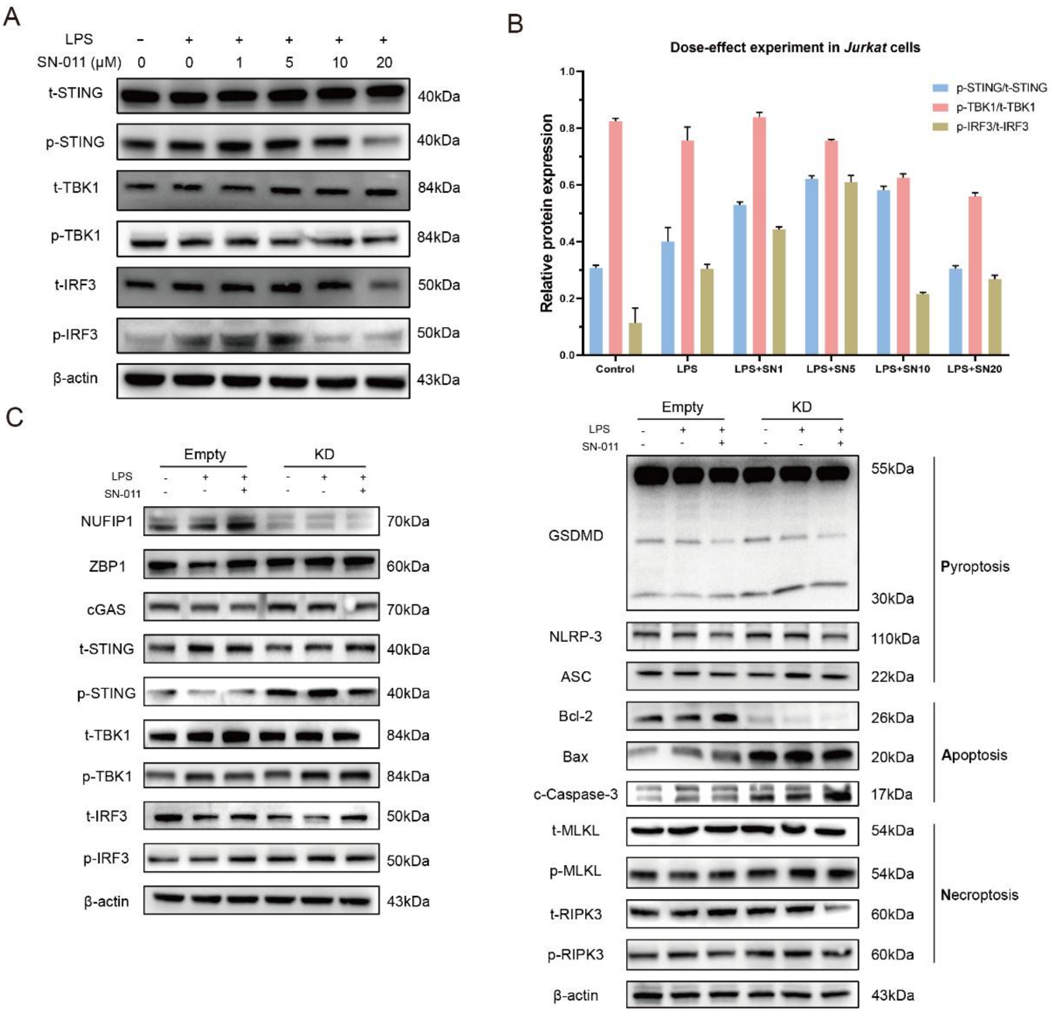

Fig. S7. Dose-response experiment of Jurkat T cells treated with SN-011 and the regulatory effects of cGAS-STING signaling pathway on Jurkat T cells PANoptosis in sepsis. (A and B) The optimal concentration of SN-011 on Jurkat T cells under LPS stimulation was determined by WB. (C) The effect of SN-011 on cGAS-STING-signaling and PANoptosis-related protein expression in Jurkat T cells under LPS stimulation was detected by WB. Data were expressed as means ± SEM.
