# Supplementary material for: NUFIP1-Mediated Ribophagy Alleviates PANoptosis of CD4+ T Lymphocytes in Sepsis via the cGAS-STING Pathway
Source: Research (Wash D C). 2025 Sep 23;8:0895. doi: 10.34133/research.0895 (PMC12454940; doi:10.34133/research.0895)

Figure 1

Fig 1C

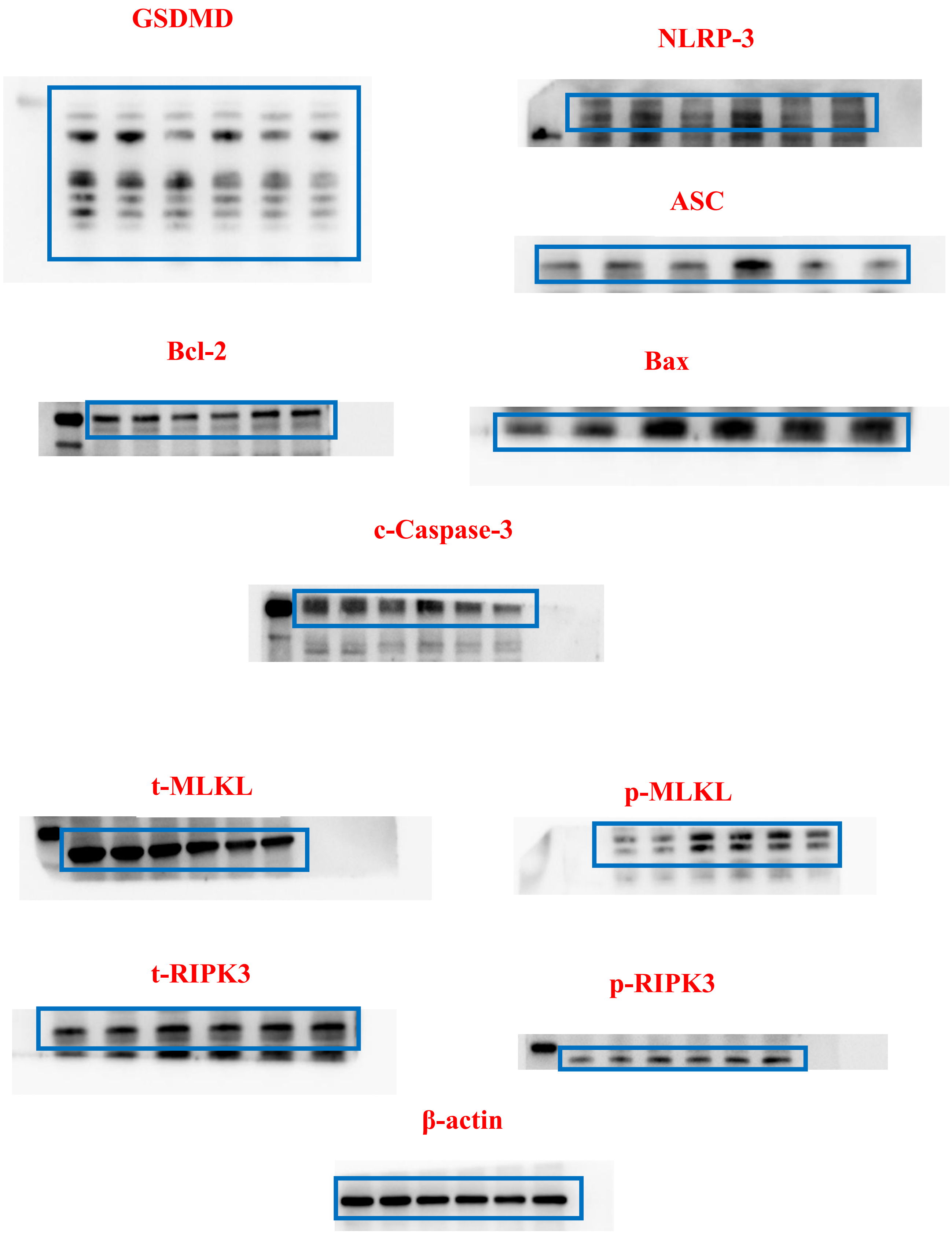

Fig 1G

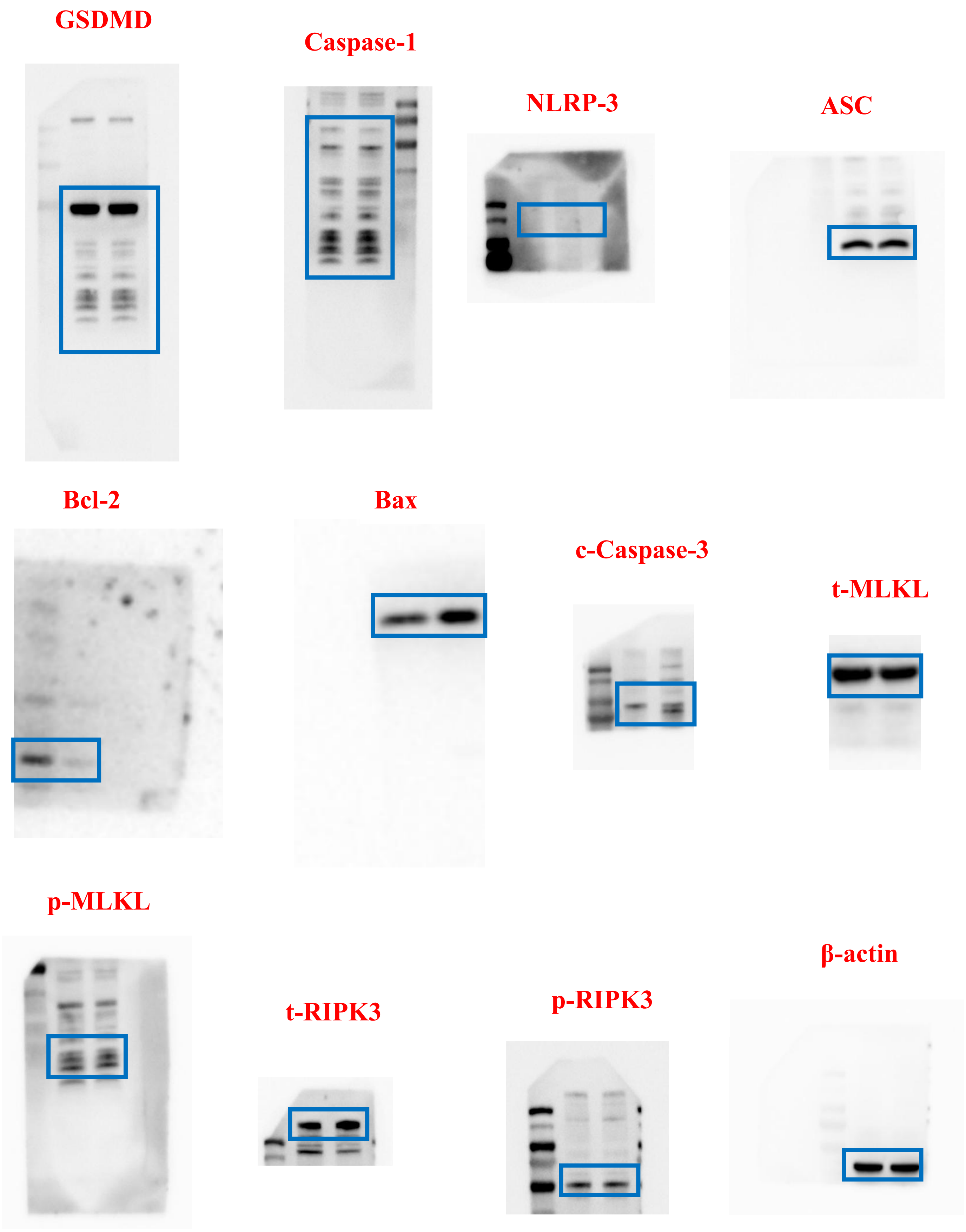
GSDMD
Caspase-1
NLRP-3
ASC
Bcl-2
Bax
c-Caspase-3
t-MLKL
p-MLKL
t-RIPK3
p-RIPK3
β-actin

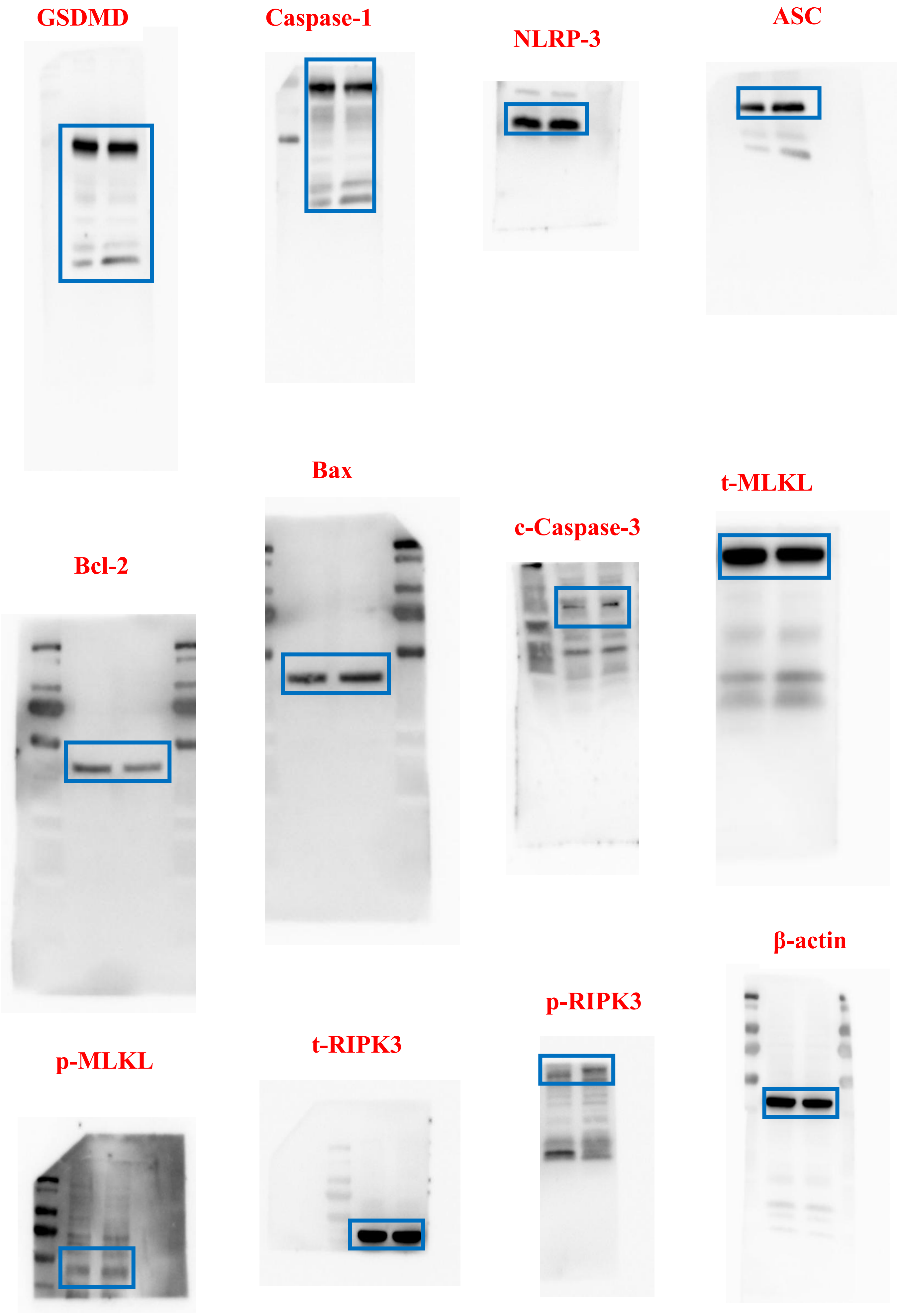
GSDMD
Caspase-1
NLRP-3
ASC
Bax
Bcl-2
c-Caspase-3
t-MLKL
β-actin
p-RIPK3
t-RIPK3
p-MLKL

GSDMD

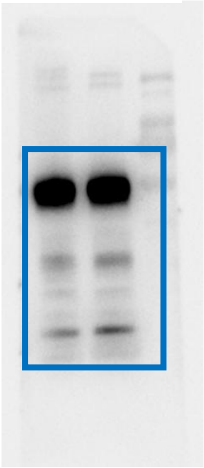

Caspase-1

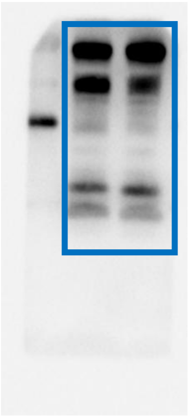

NLRP-3

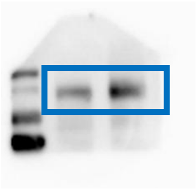

ASC

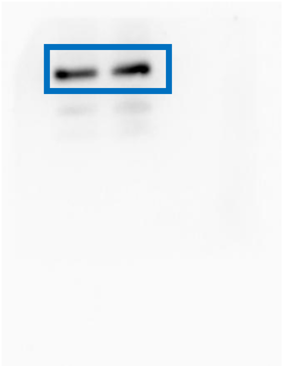

Bcl-2

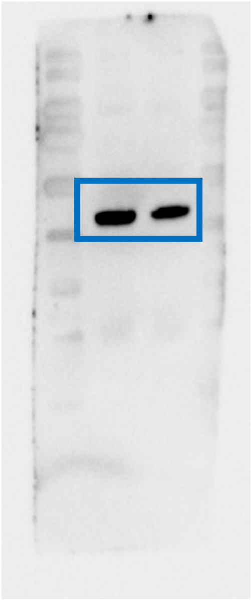

Bax

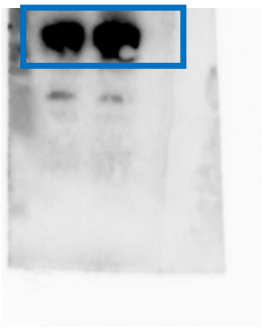

c-Caspase-3

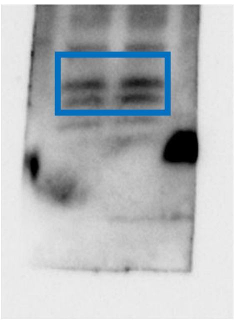

t-MLKL

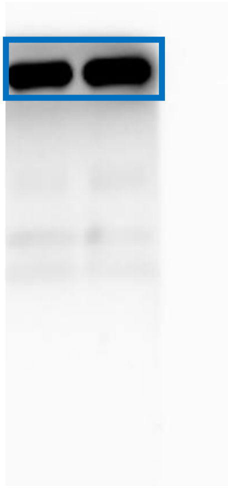

β-actin

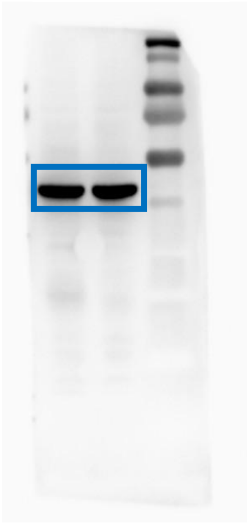

p-MLKL

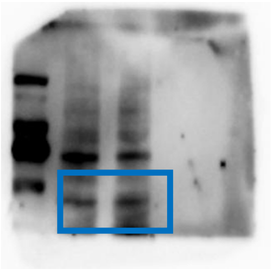

t-RIPK3

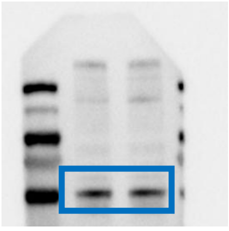

p-RIPK3

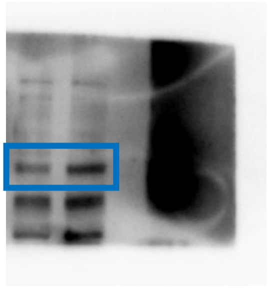

Figure 2

Fig 2B

NUFIP1

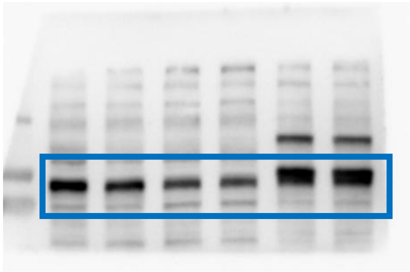

β-actin

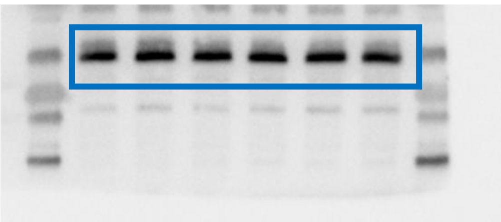

Fig 2F

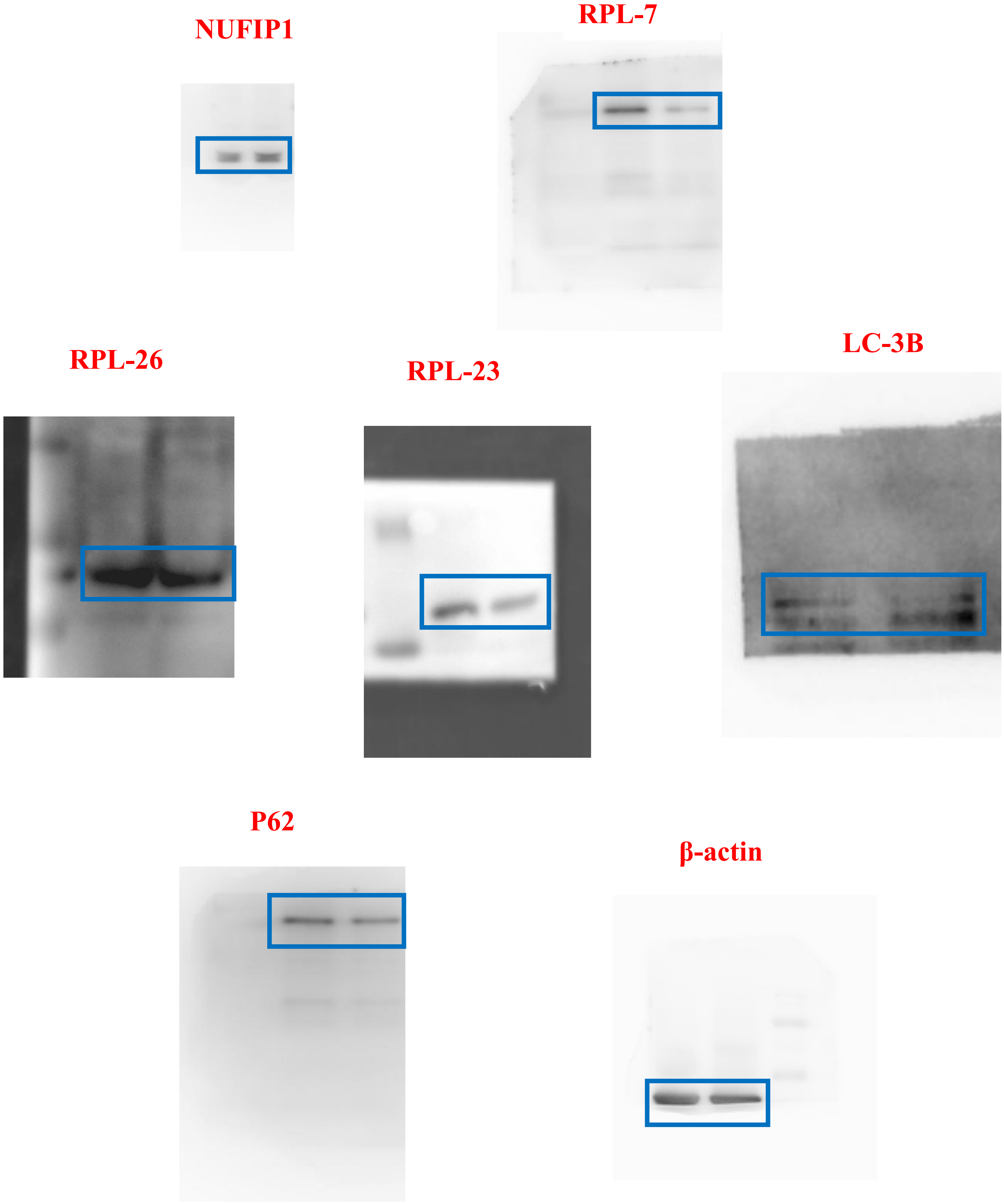

Fig 2G

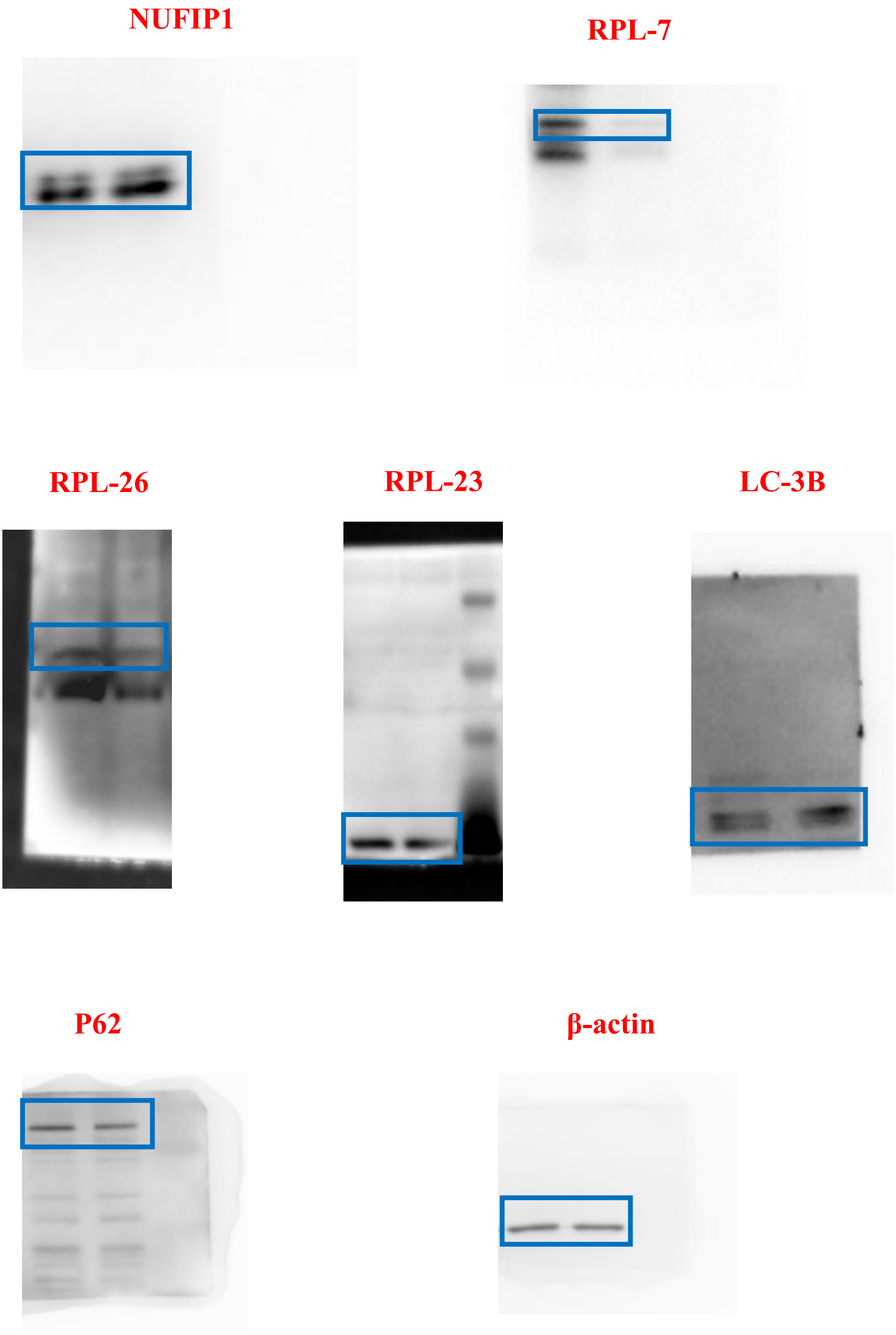

Fig 2H

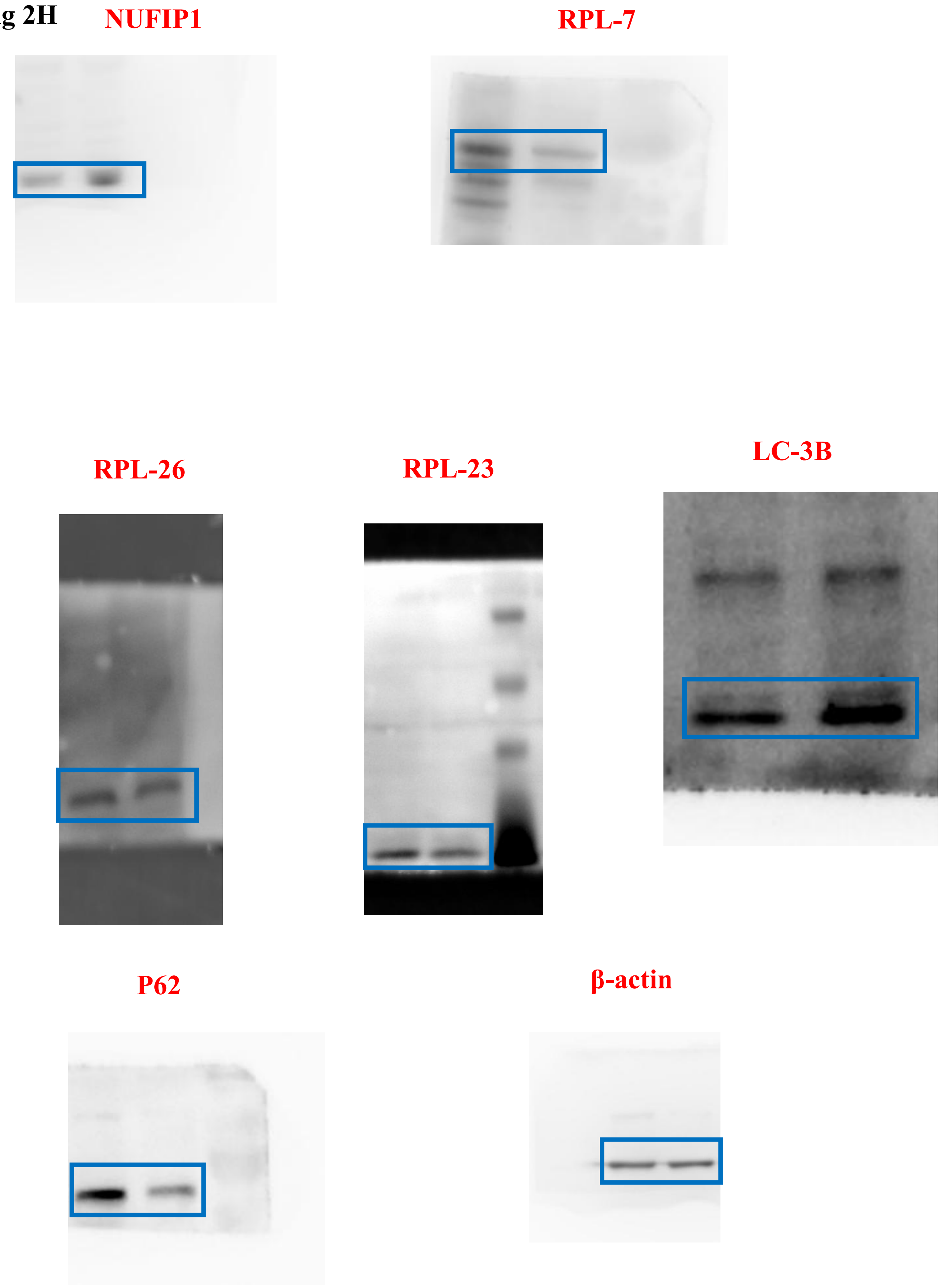

Figure 3

Fig 3C

GSDMD

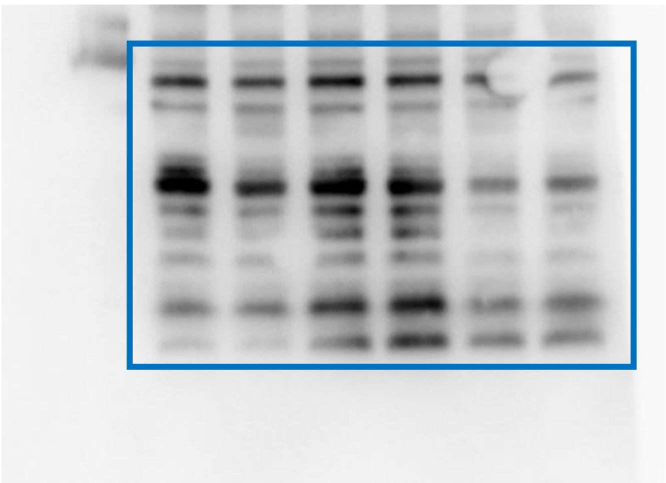

Caspase-1

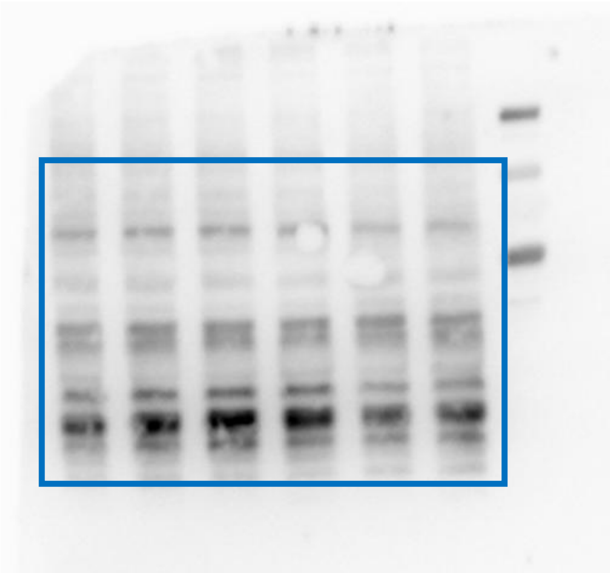

NLRP-3

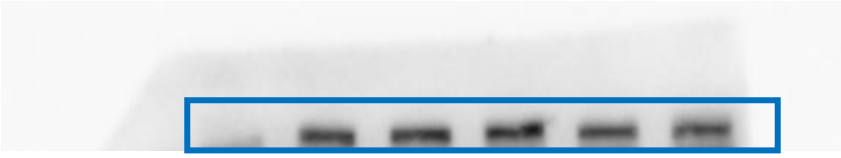

ASC

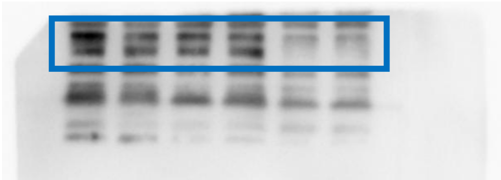

β-actin

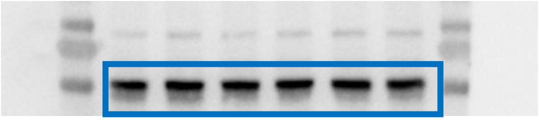

Bcl-2

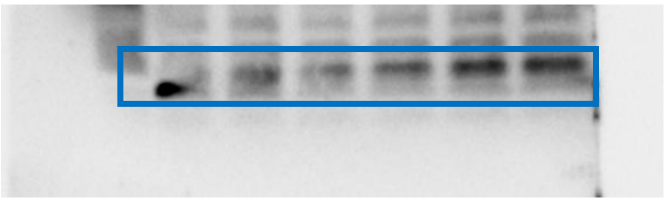

Bax

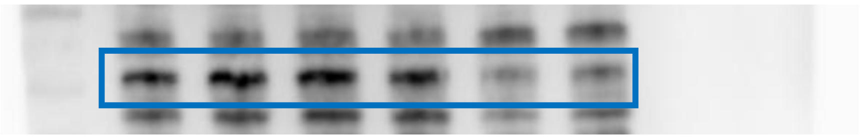

c-Caspase-3

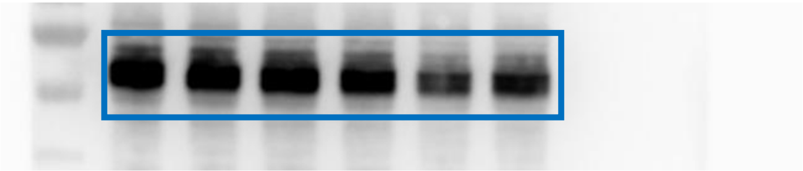

β-actin

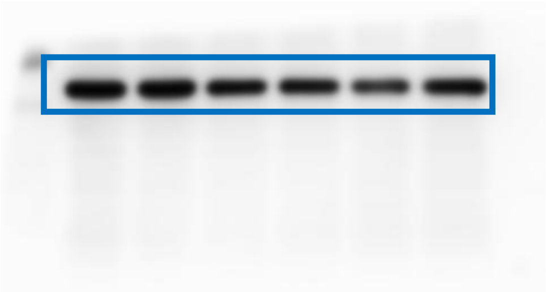

t-MLKL

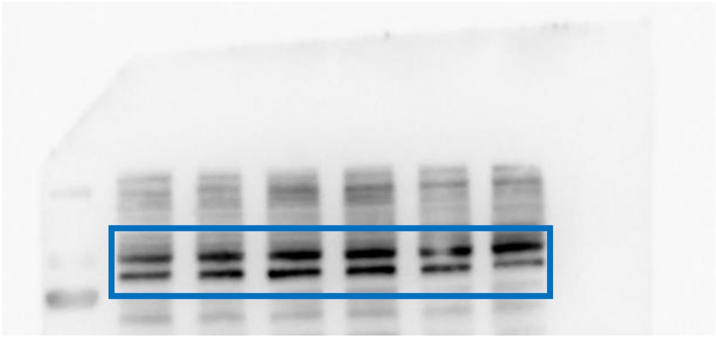

p-MLKL

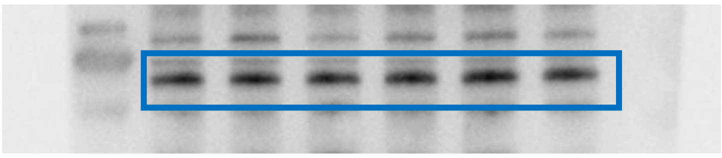

t-RIPK3

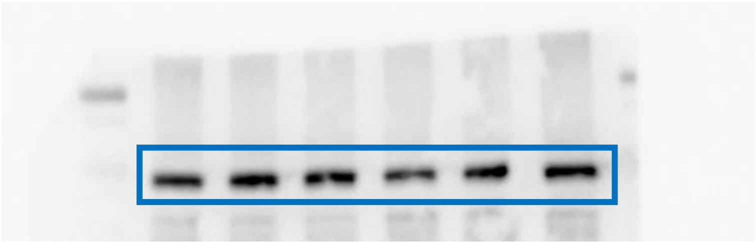

p-RIPK3

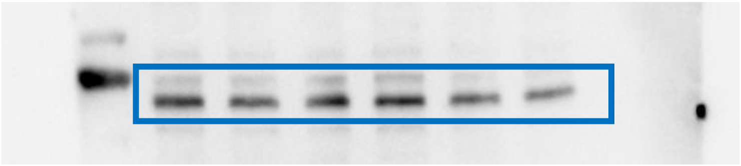

β-actin

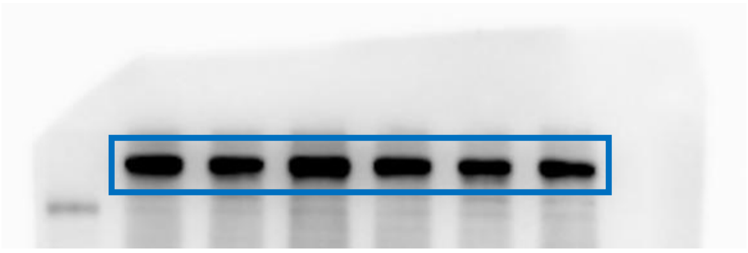

Fig 3E

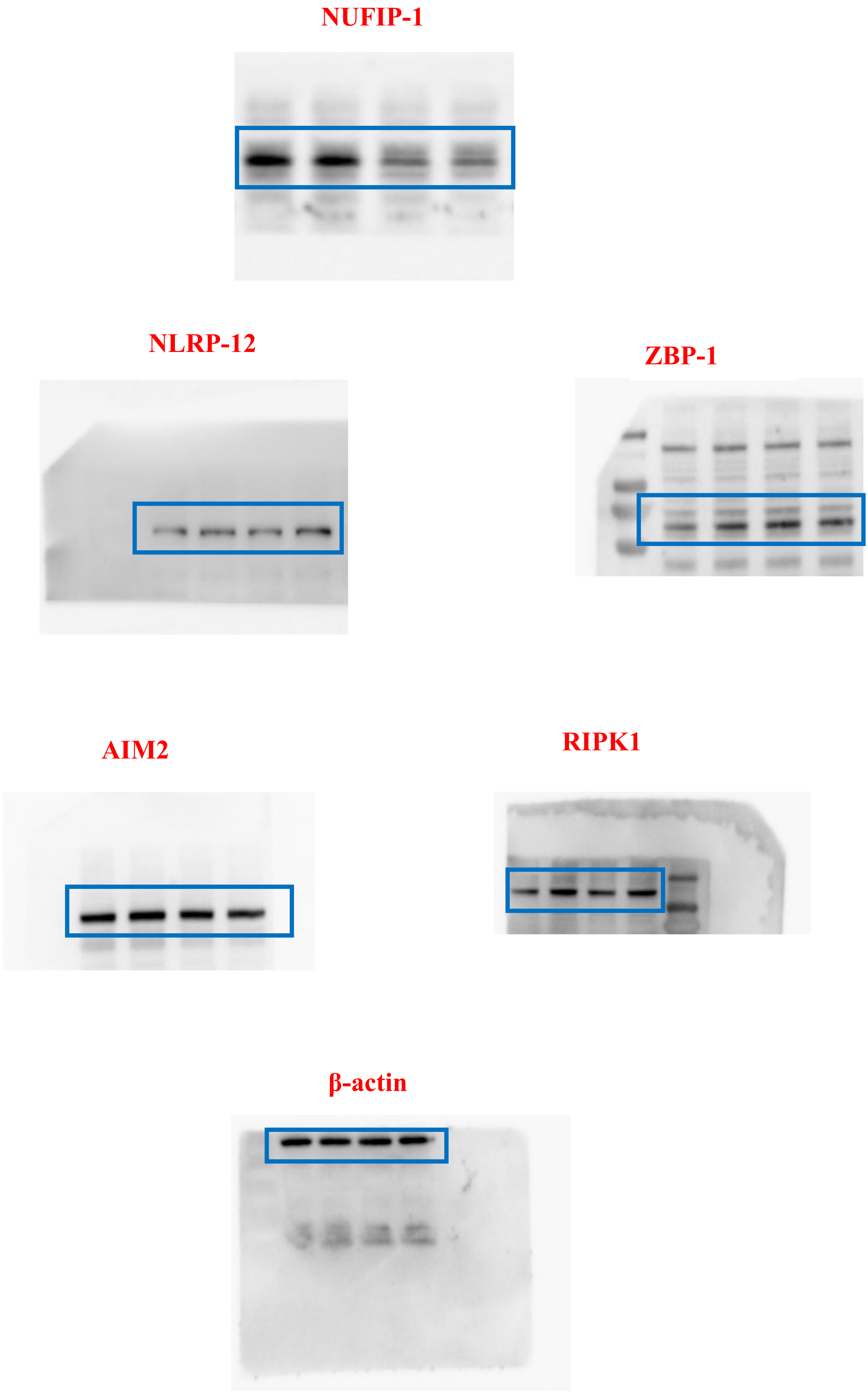

Fig 3G

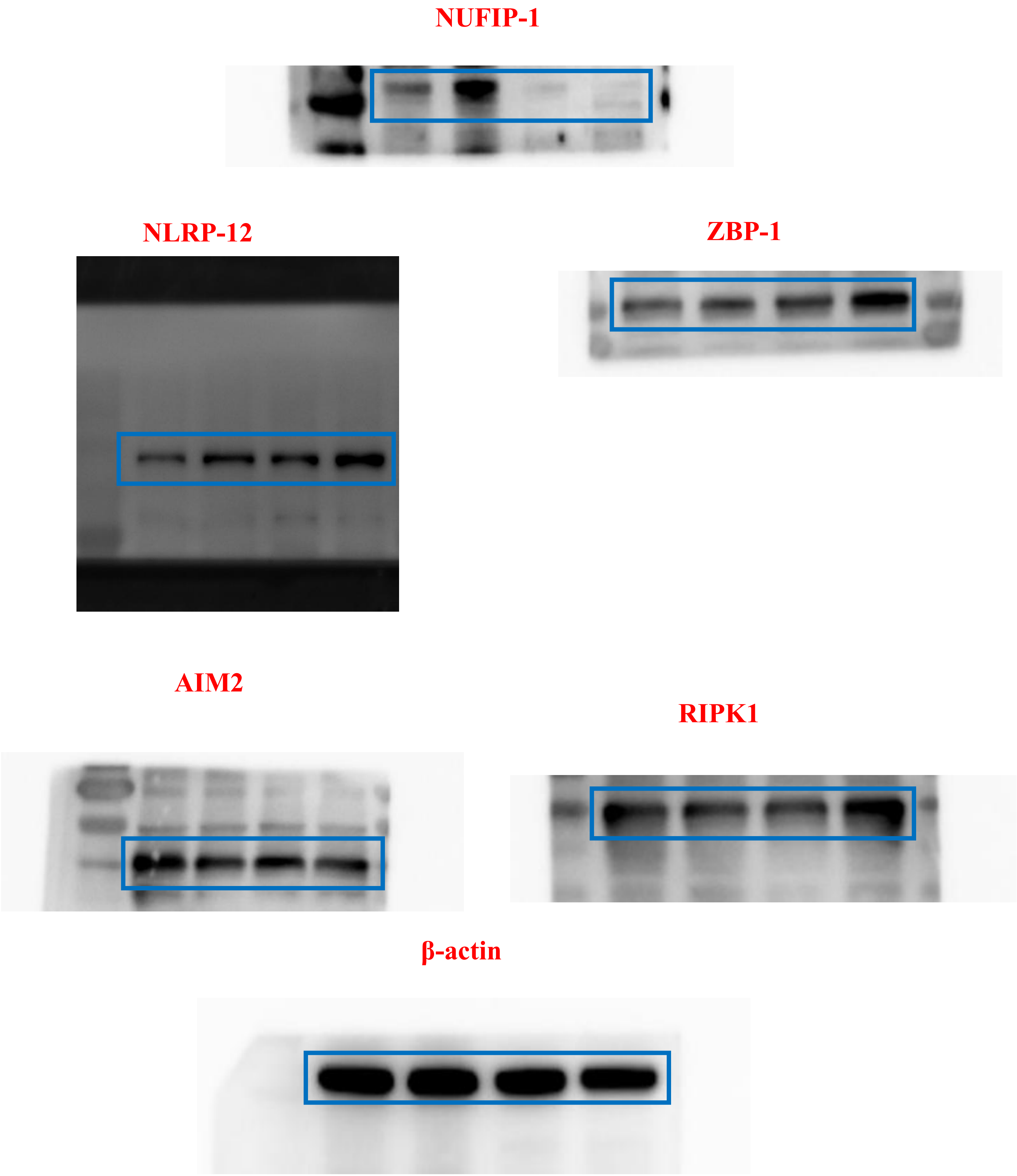

Fig 3I

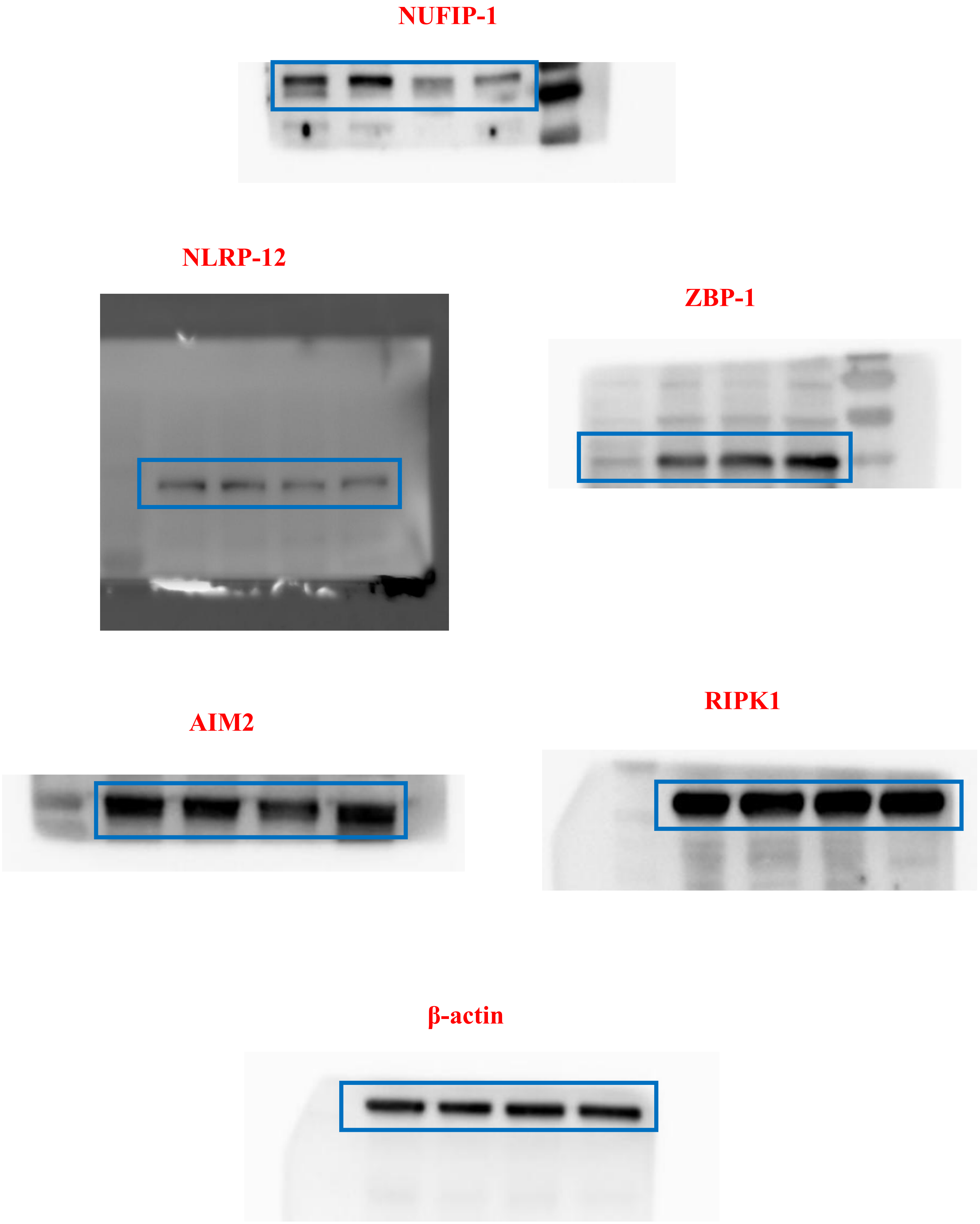

Figure 4

Fig 4E

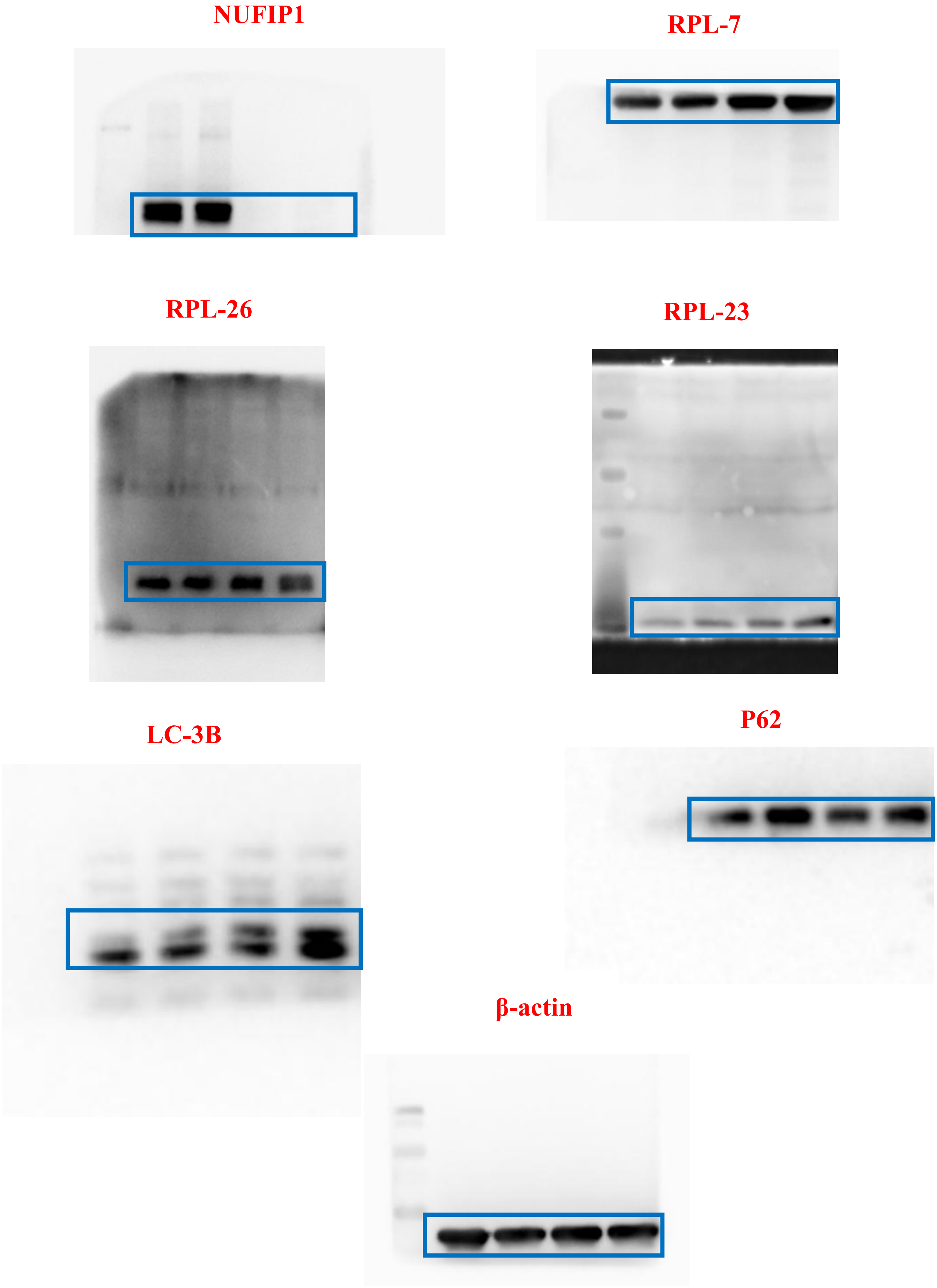

GSDMD

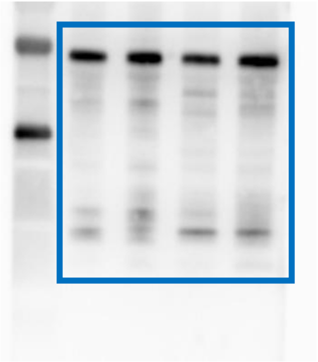

Caspase-1

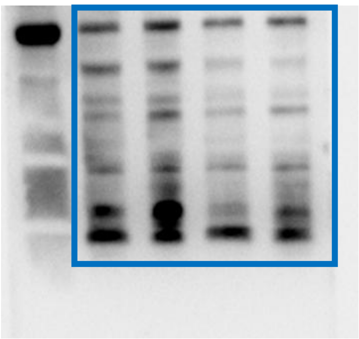

NLRP-3

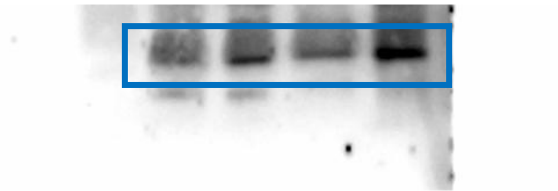

ASC

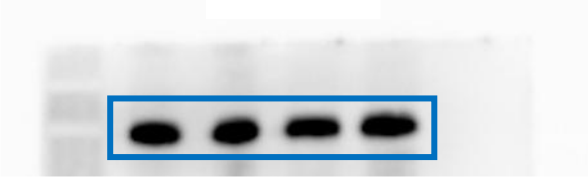

β-actin

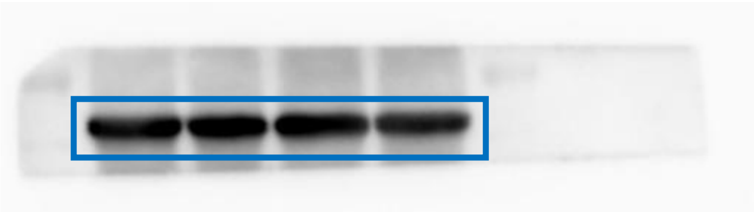

Bcl-2

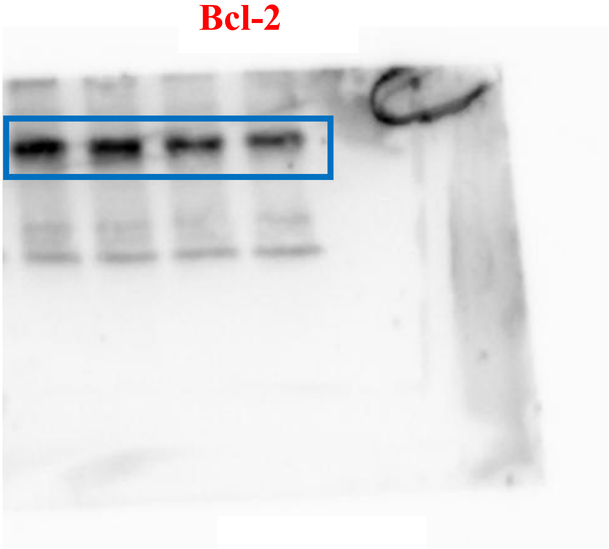

Bax

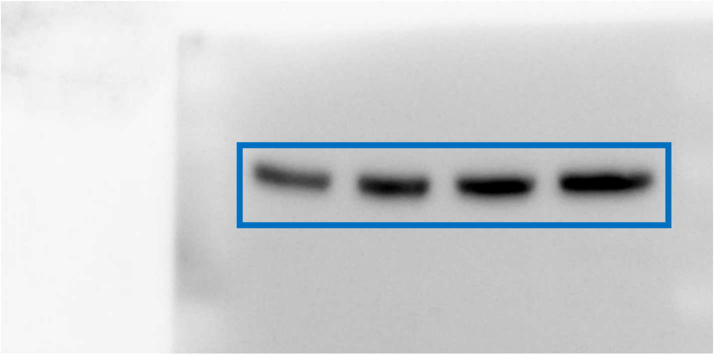

c-Caspase-3

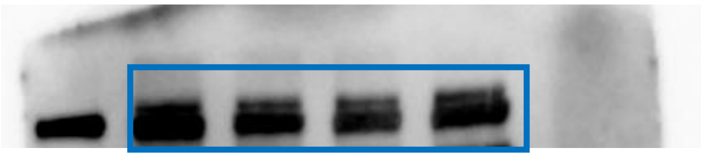

β-actin

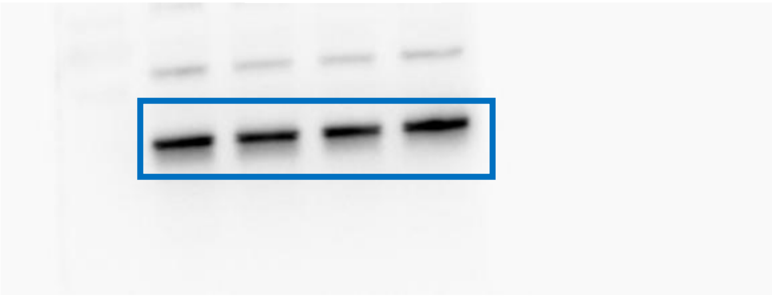

t-MLKL

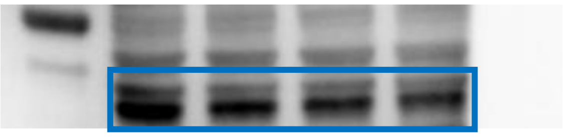

p-MLKL

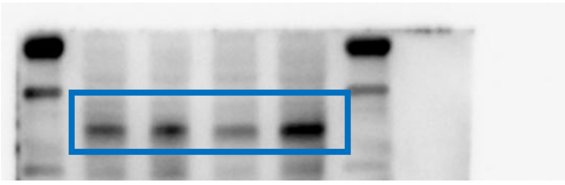

t-RIPK3

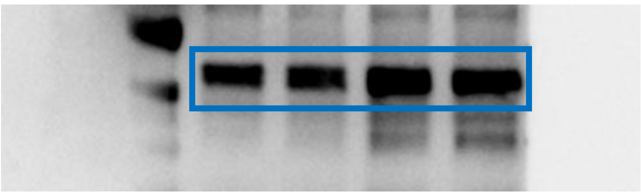

p-RIPK3

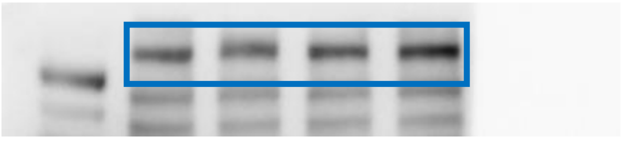

β-actin

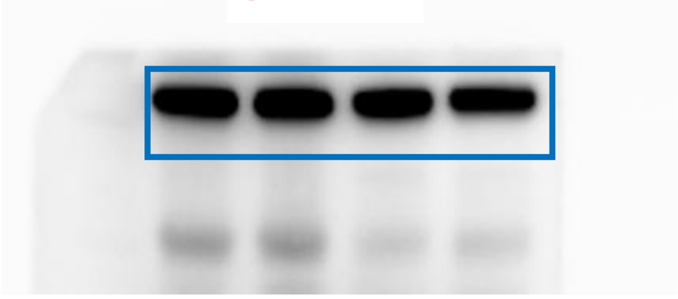

Figure 6

Fig 6C

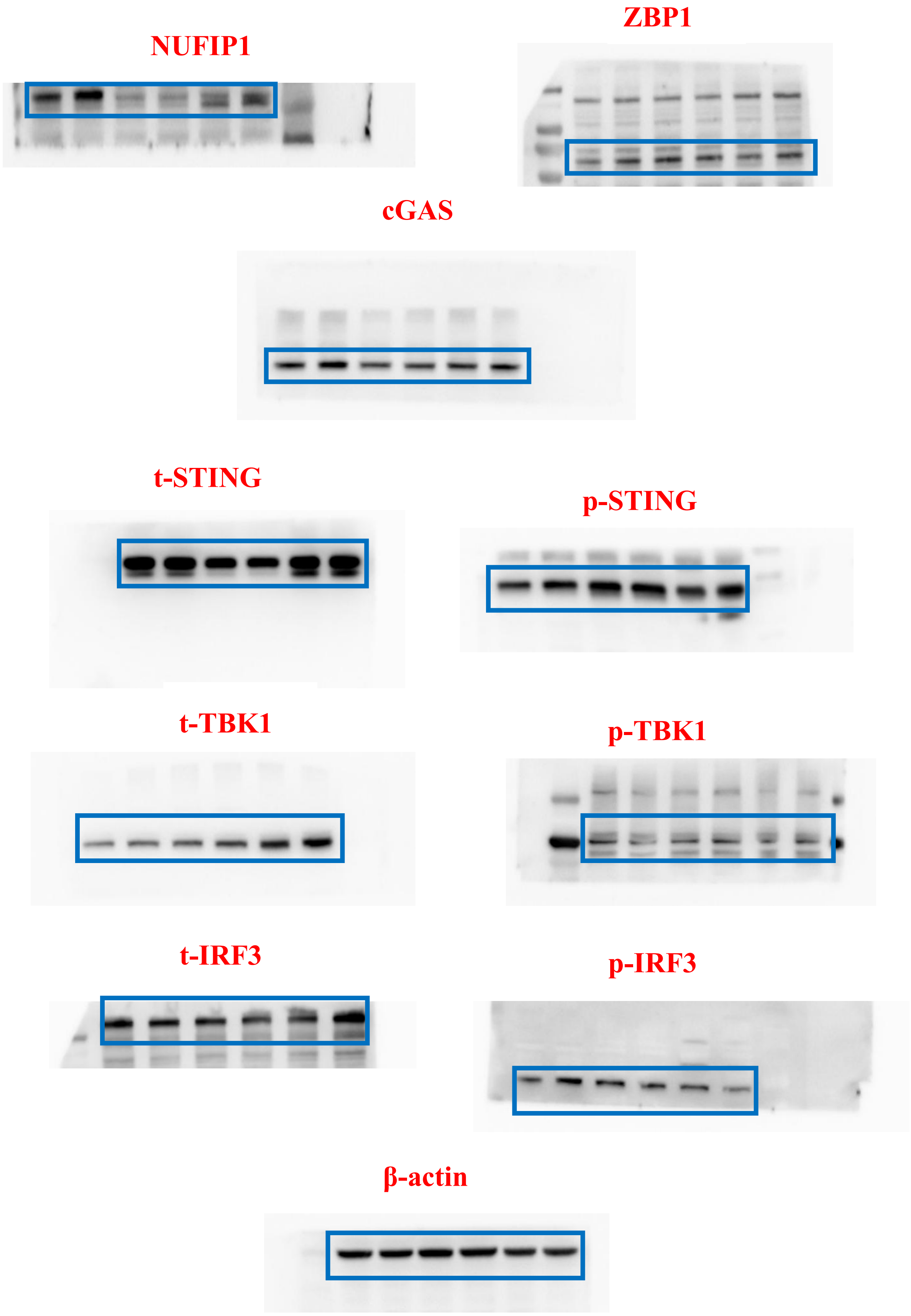

Fig 6E

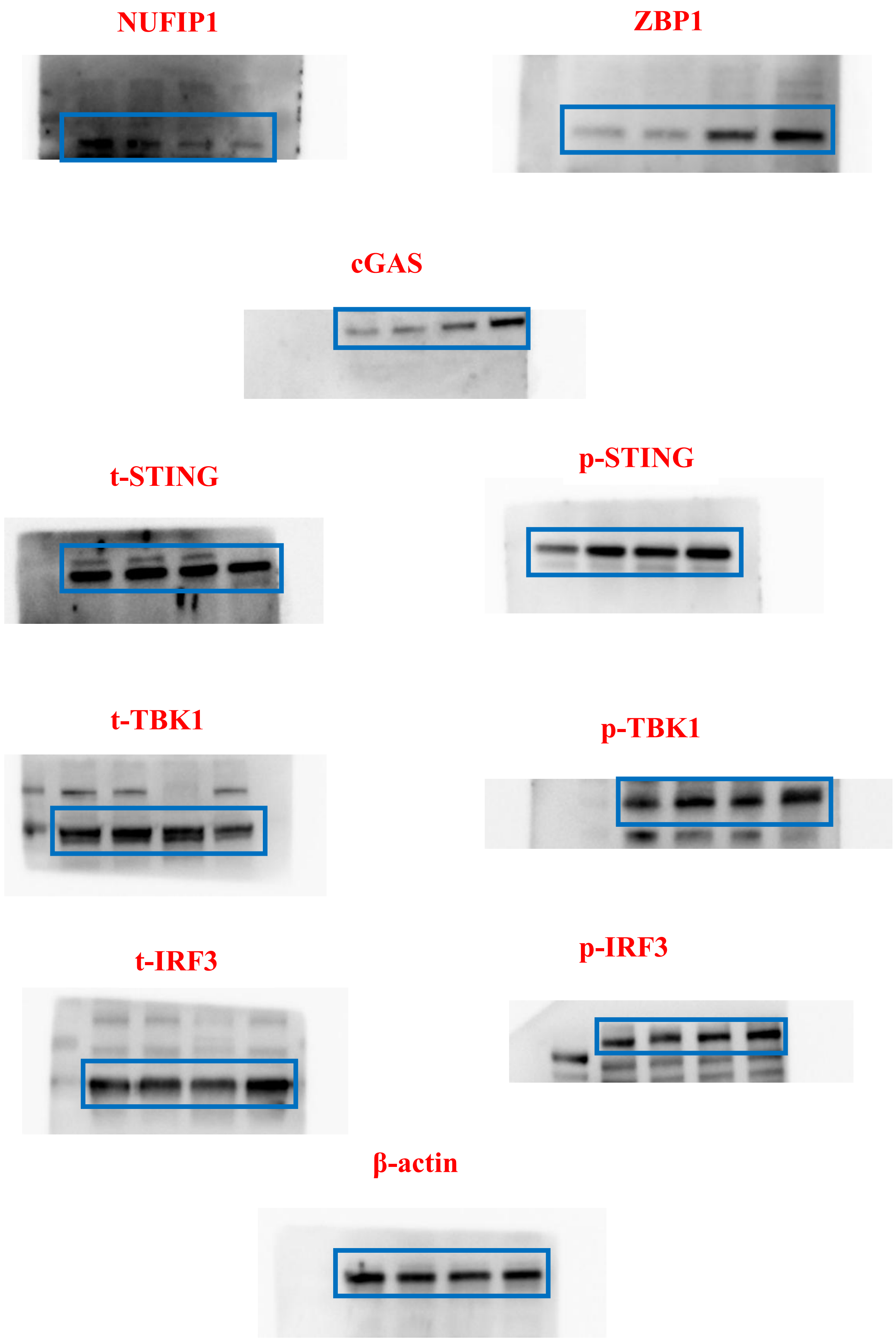
NUFIP1
ZBP1
cGAS
t-STING
p-STING
t-TBK1
p-TBK1
t-IRF3
p-IRF3
β-actin

Fig 6J

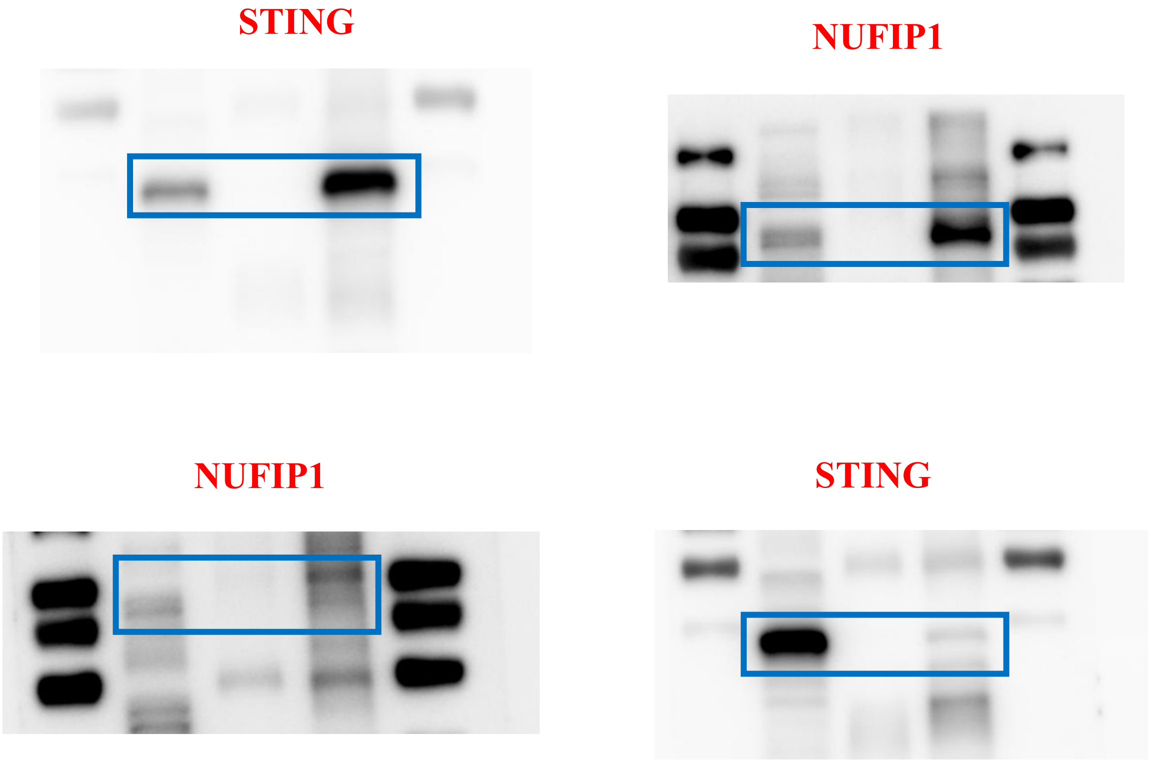

Figure 7

Fig 7A

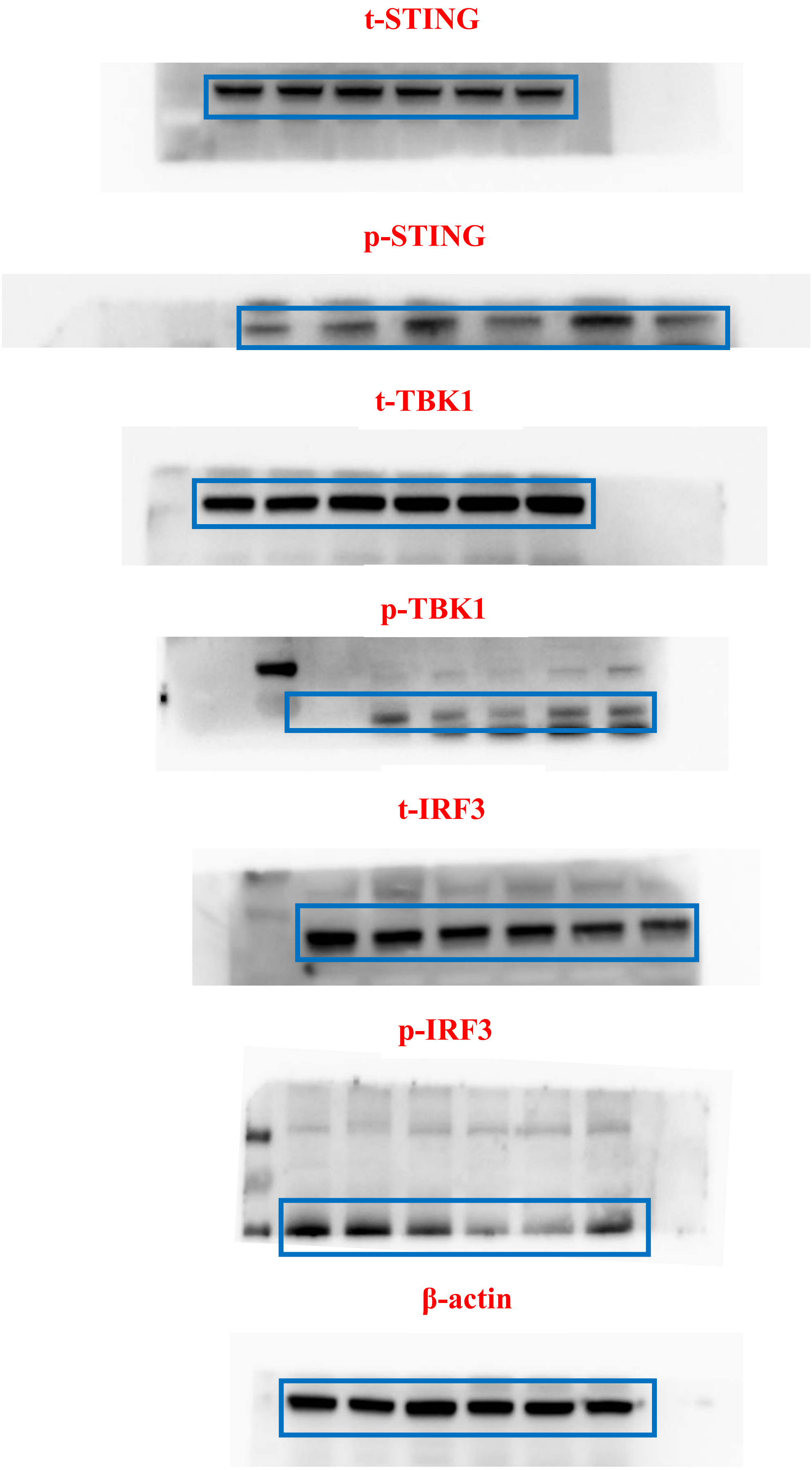

Fig 7D

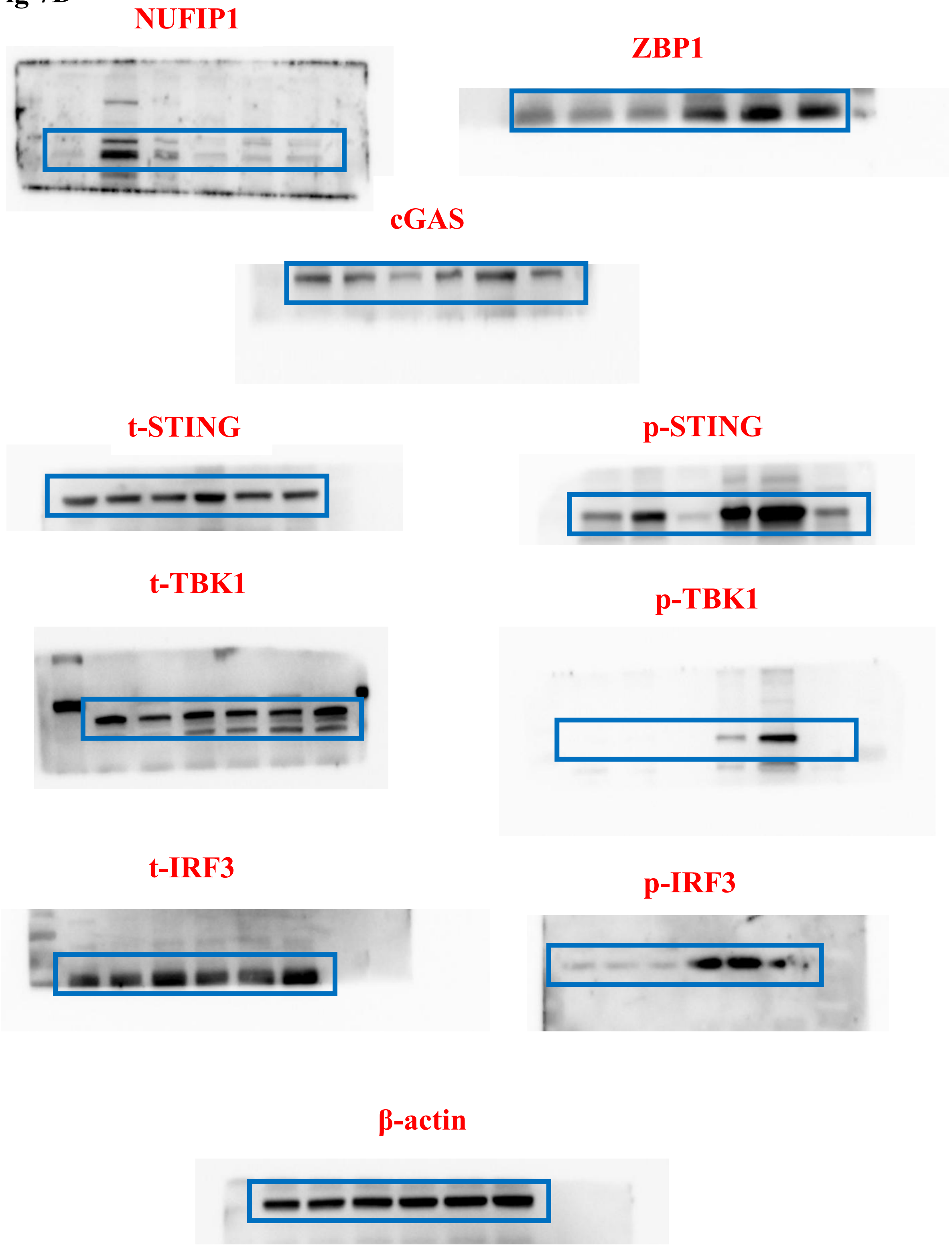
NUFIP1
ZBP1
cGAS
t-STING
p-STING
t-TBK1
p-TBK1
t-IRF3
p-IRF3
β-actin

GSDMD

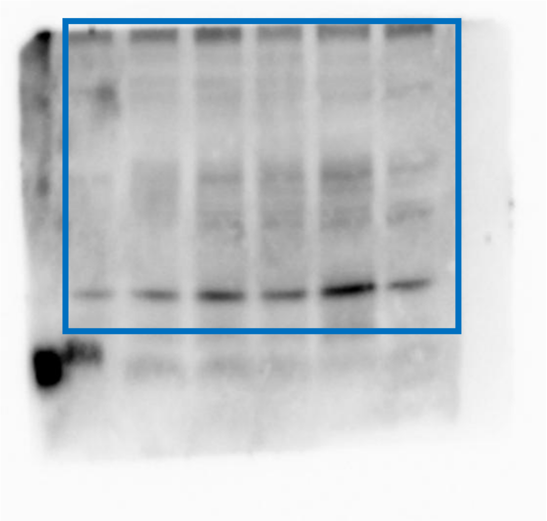

NLRP3

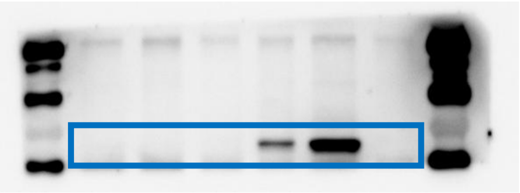

ASC

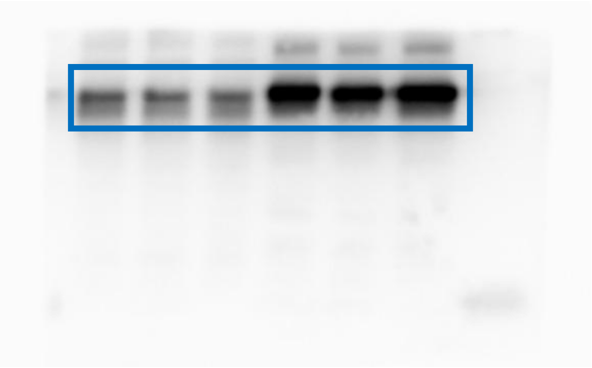

Bcl-2

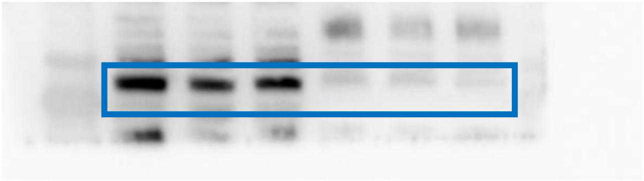

Bax

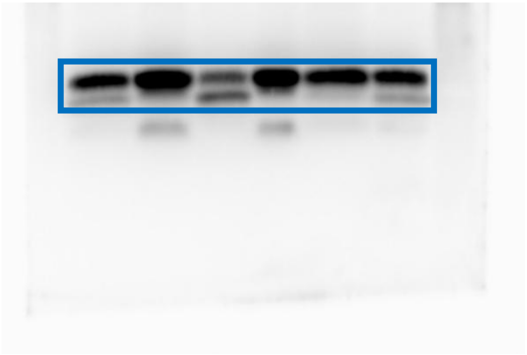

c-Caspase3

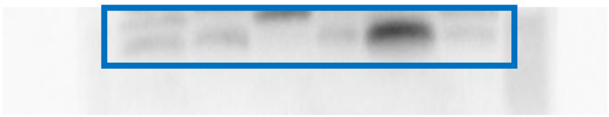

t-MLKL

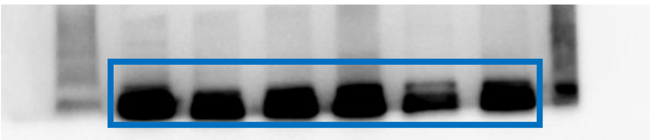

p-MLKL

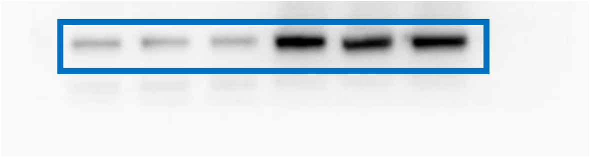

t-RIPK3

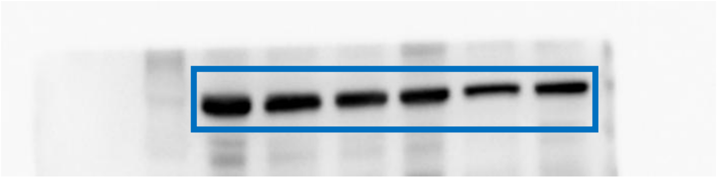

p-RIPK3

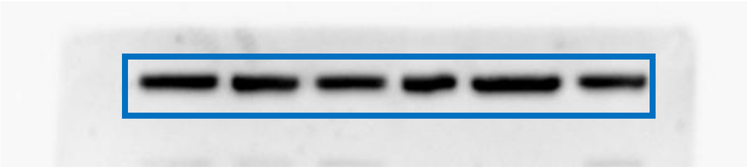

β-actin

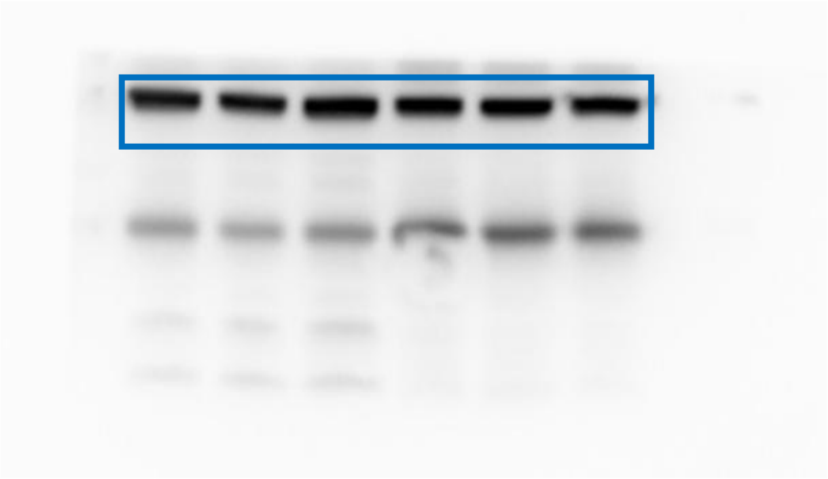

Fig 7E

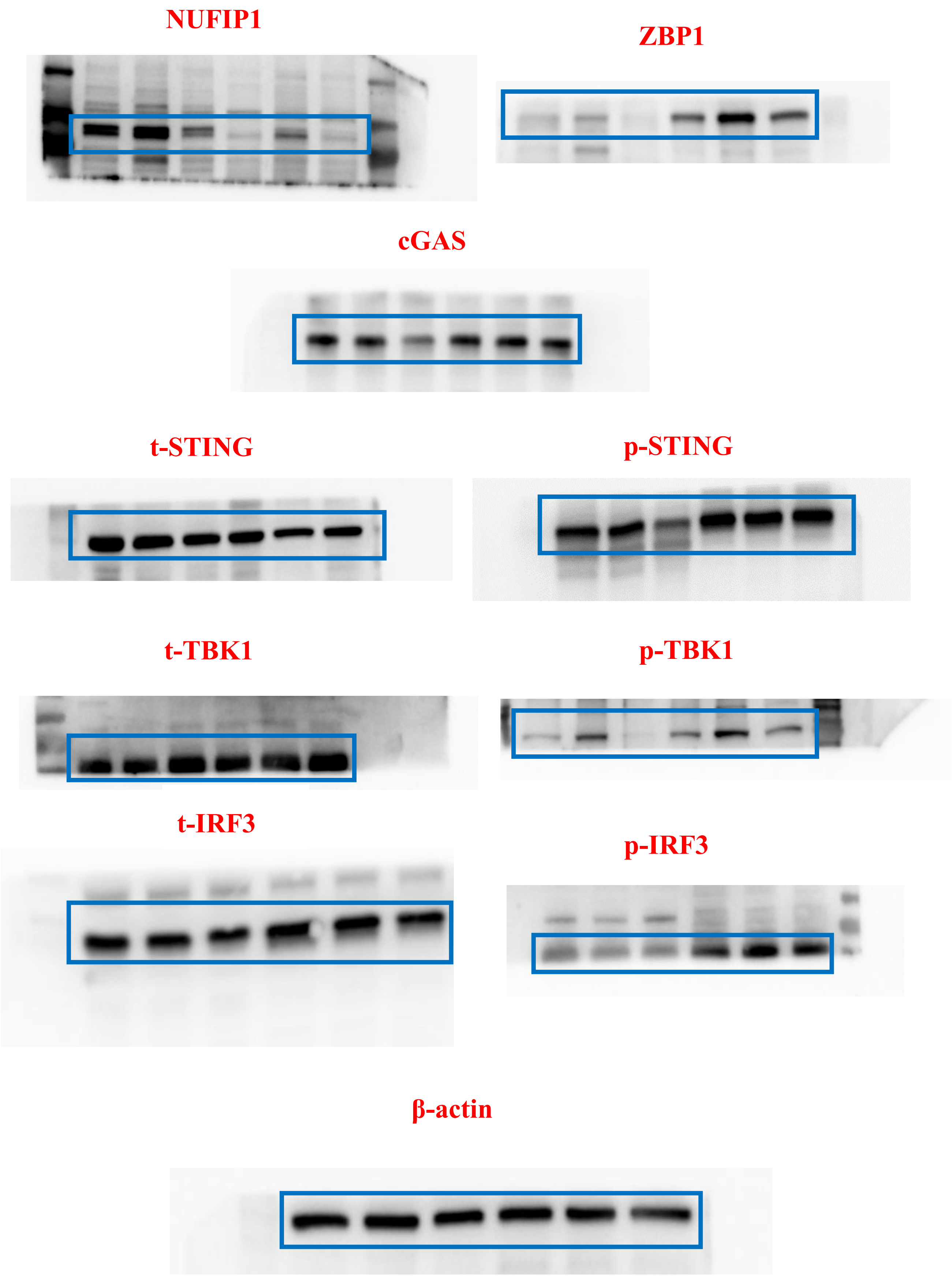
NUFIP1
ZBP1
cGAS
t-STING
p-STING
t-TBK1
p-TBK1
t-IRF3
p-IRF3
β-actin

GSDMD

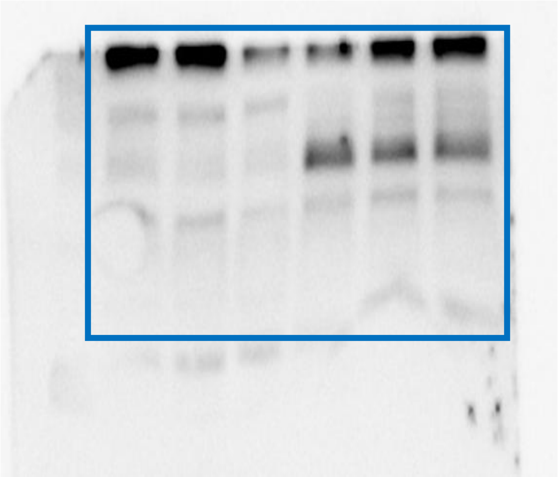

NLRP3

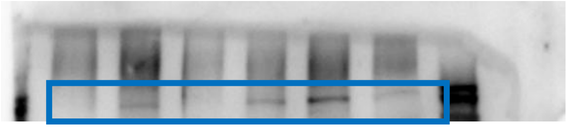

ASC

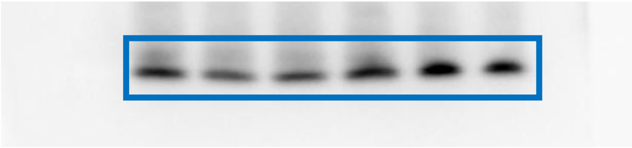

Bcl-2

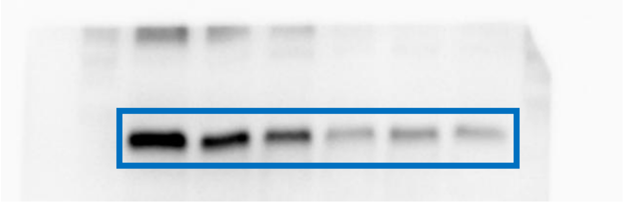

Bax

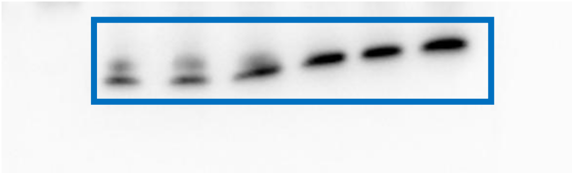

c-Caspase3

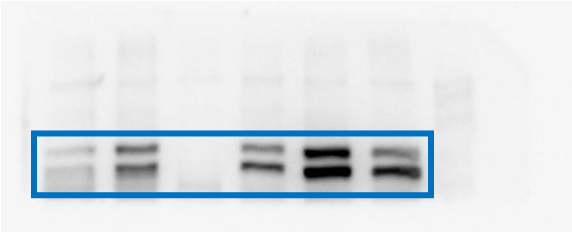

t-MLKL

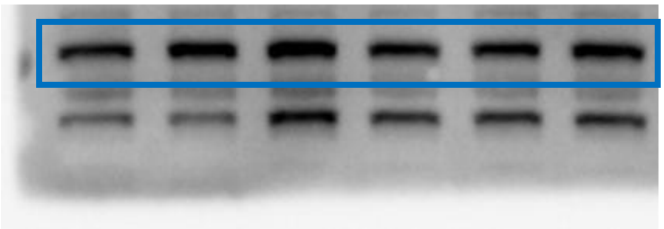

p-MLKL

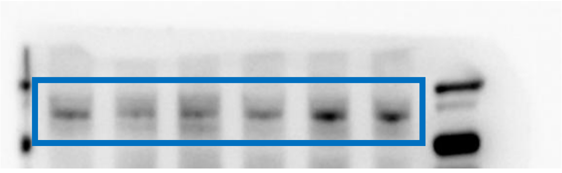

t-RIPK3

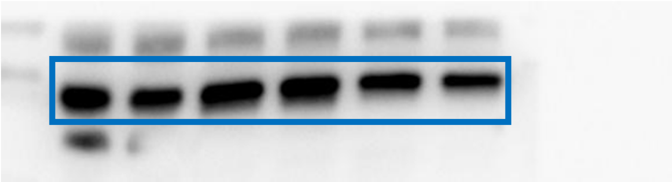

p-RIPK3

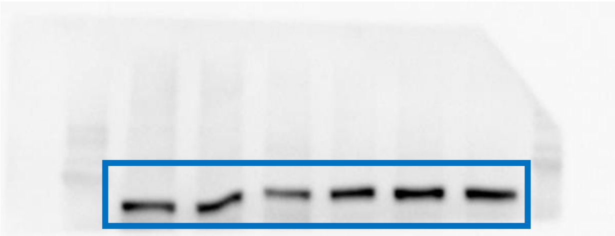

β-actin

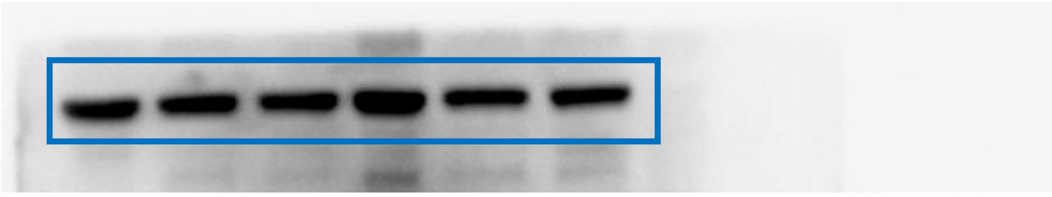

Figure 8

Fig 8K

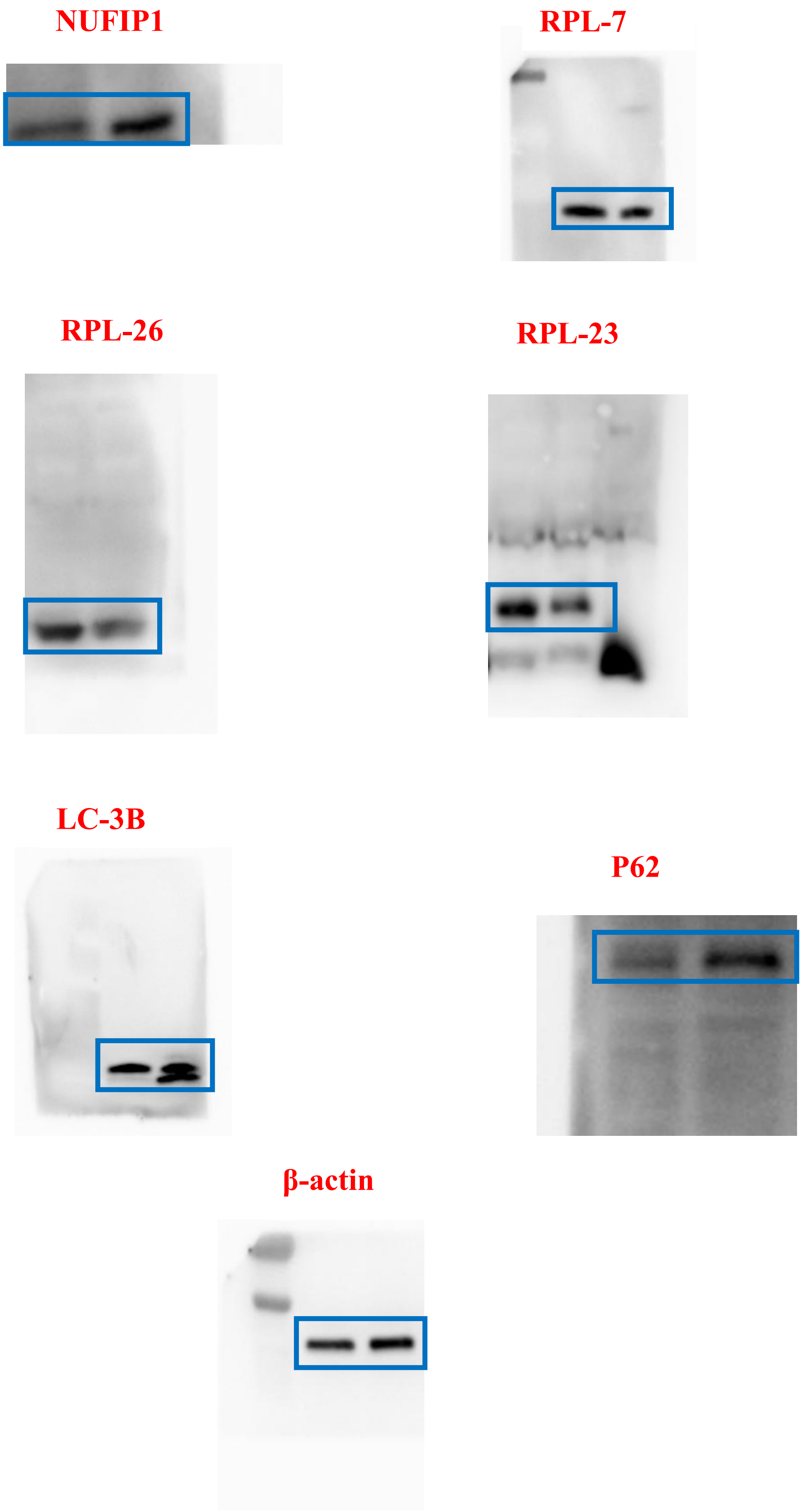

GSDMD

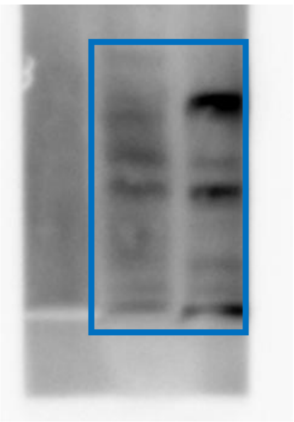

NLRP3

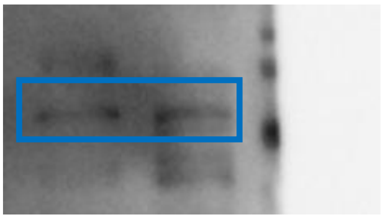

ASC

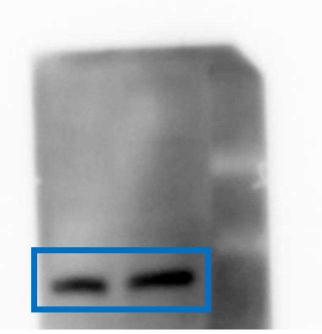

Bcl-2

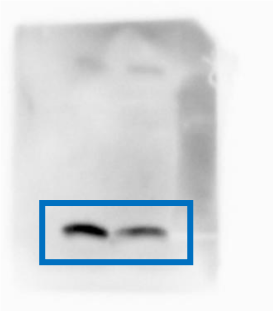

Bax

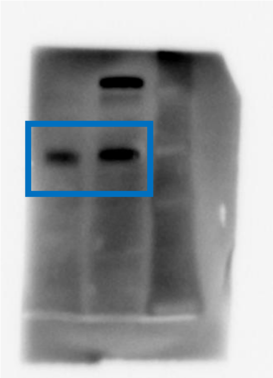

c-Caspase3

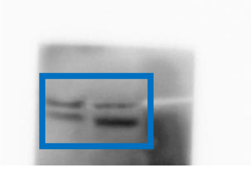

t-MLKL

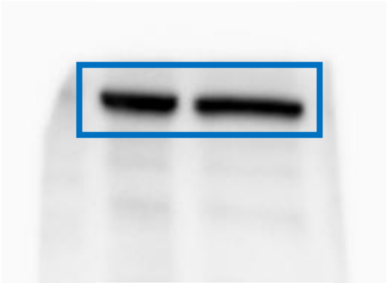

p-MLKL

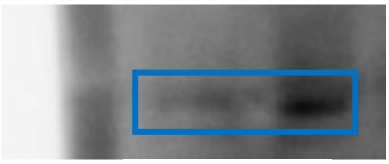

t-RIPK3

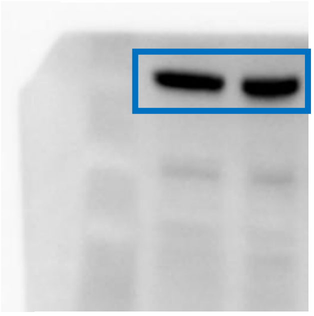

p-RIPK3

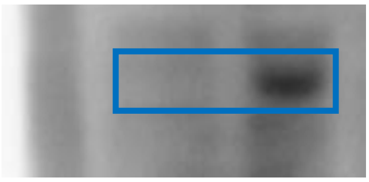

β-actin

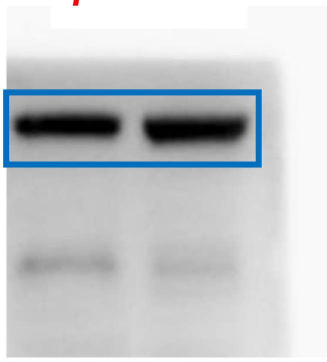

Supplement: Supplementary 1 — Supplementary Text Tables S1 to S3 Figs. S1 to S7 [file research.0895.f1.zip › Supplemental File 1 Original data of the WB.pdf]
