# Supplementary material for: NUFIP1-Mediated Ribophagy Alleviates PANoptosis of CD4+ T Lymphocytes in Sepsis via the cGAS-STING Pathway
Source: Research (Wash D C). 2025 Sep 23;8:0895. doi: 10.34133/research.0895 (PMC12454940; doi:10.34133/research.0895)
Supplement: Supplementary 1 — Supplementary Text Tables S1 to S3 Figs. S1 to S7 [file research.0895.f1.zip › Supplemental File 2 Certificate of English Editing..pdf]

Freescience Editorial Team

Certificate of English Editing

Paper Title

NUFIP1-mediated Ribophagy Alleviates PANoptosis of $CD4^{+}$ T lymphocytes in Sepsis via the cGAS-STING Pathway

Authors

Pengyue Zhao, Jingyan Li, Pengyi He, Yao Wu, Liyu Zheng, Xingpeng Yang, Jiaqi Yang, Ze Fu, Yun Xia, Ning Chen, Ning Dong, Zhiwen Luo, Renqi Yao, Xiaohui Du, and Yongming Yao

This certificate is issued as a confirmation that the paper mentioned above has been proofread and edited for language clarity and grammar by professional editors of our company.

We guarantee that the original message was not distorted, and that the paper is understandable and free of errors assuming that the changes and suggestions given are accepted, and text is not altered without our knowledge.

Date of Editing: 2025-08-05
