# Supplementary figures and images for: NUFIP1-Mediated Ribophagy Alleviates PANoptosis of CD4+ T Lymphocytes in Sepsis via the cGAS-STING Pathway
Source: Research (Wash D C). 2025 Sep 23;8:0895. doi: 10.34133/research.0895 (PMC12454940; doi:10.34133/research.0895)

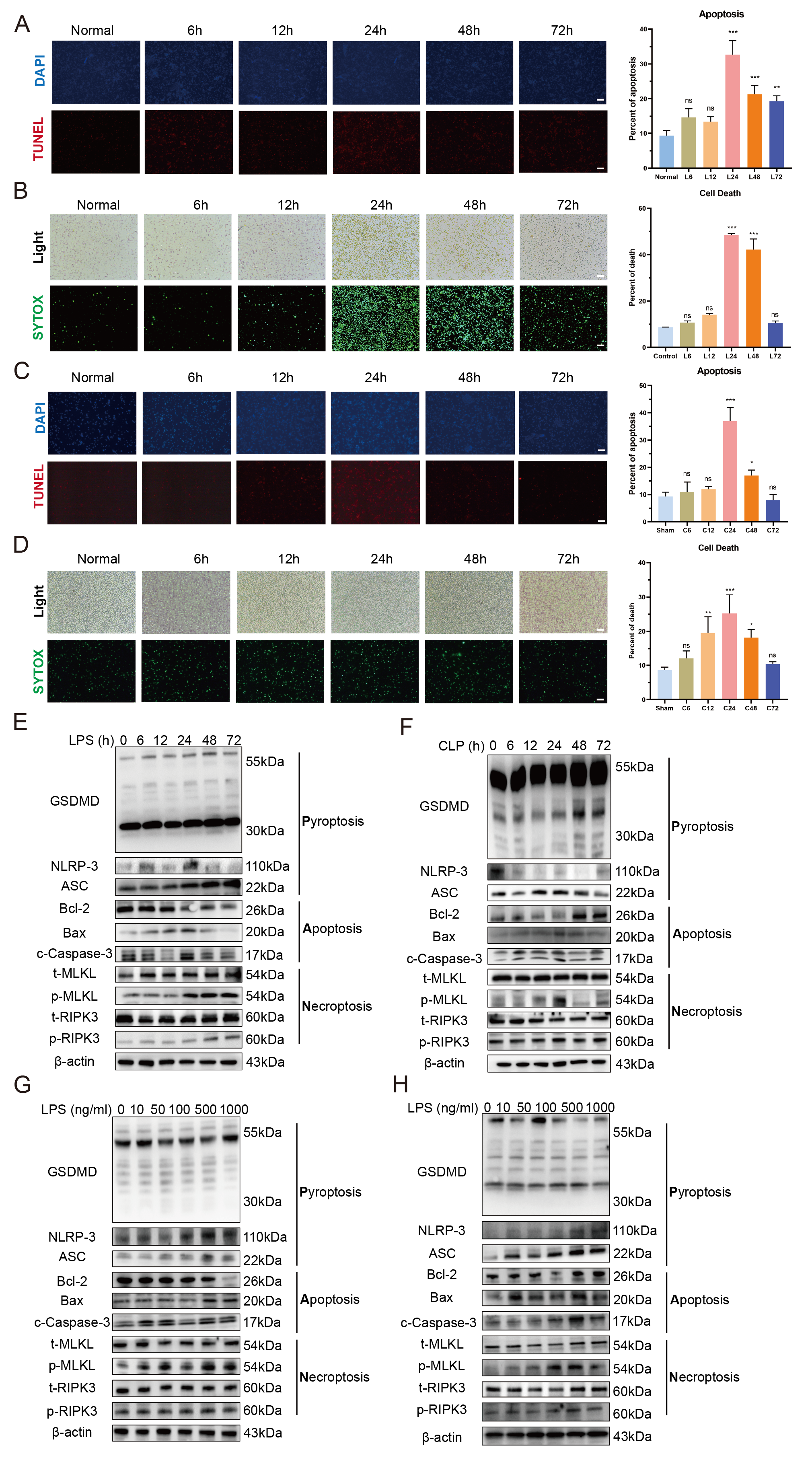

Supplement: Supplementary 1 — Supplementary Text Tables S1 to S3 Figs. S1 to S7 [file research.0895.f1.zip › Fig. S1.tif]

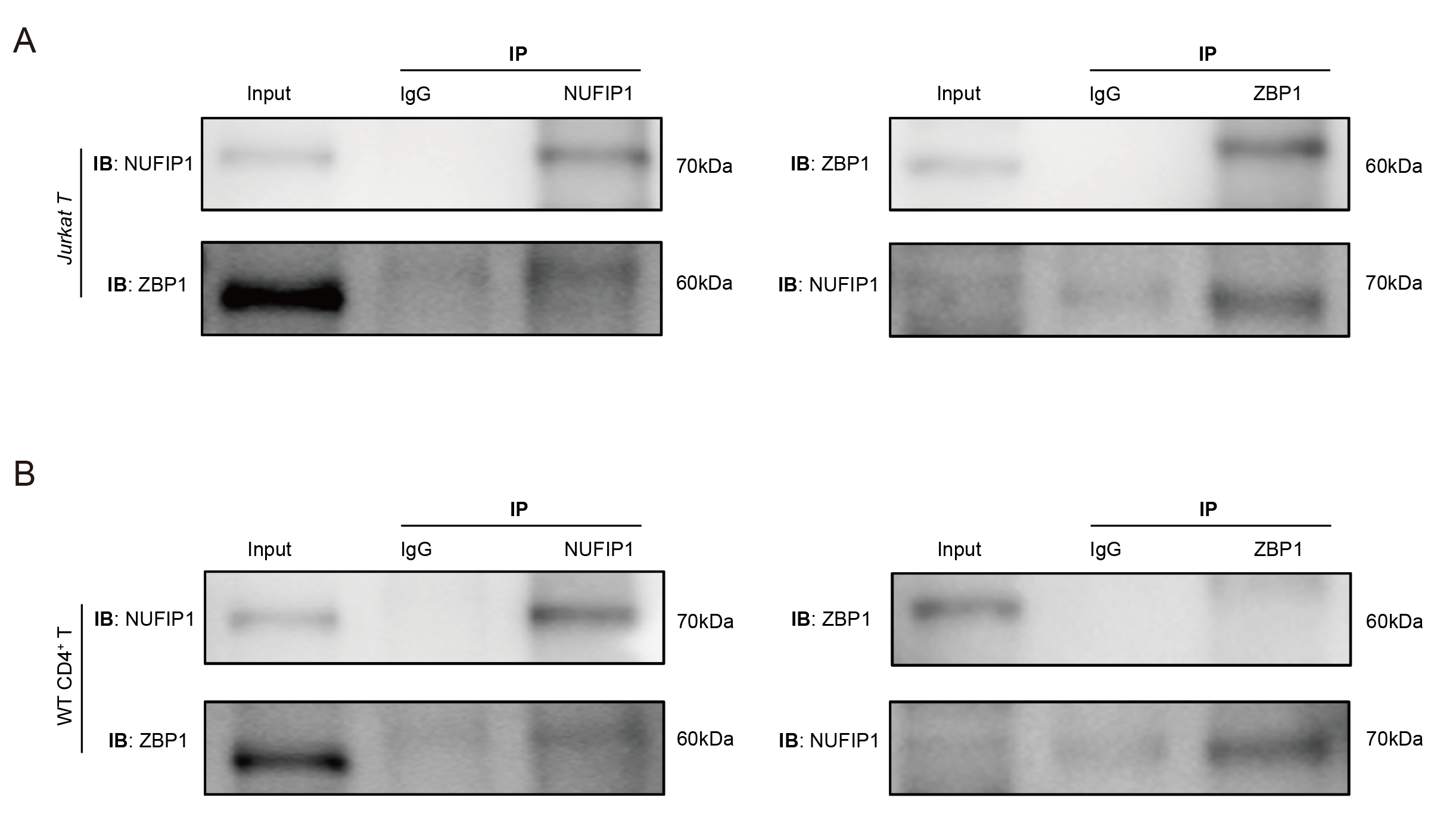

Supplement: Supplementary 1 — Supplementary Text Tables S1 to S3 Figs. S1 to S7 [file research.0895.f1.zip › Fig. S2.tif]

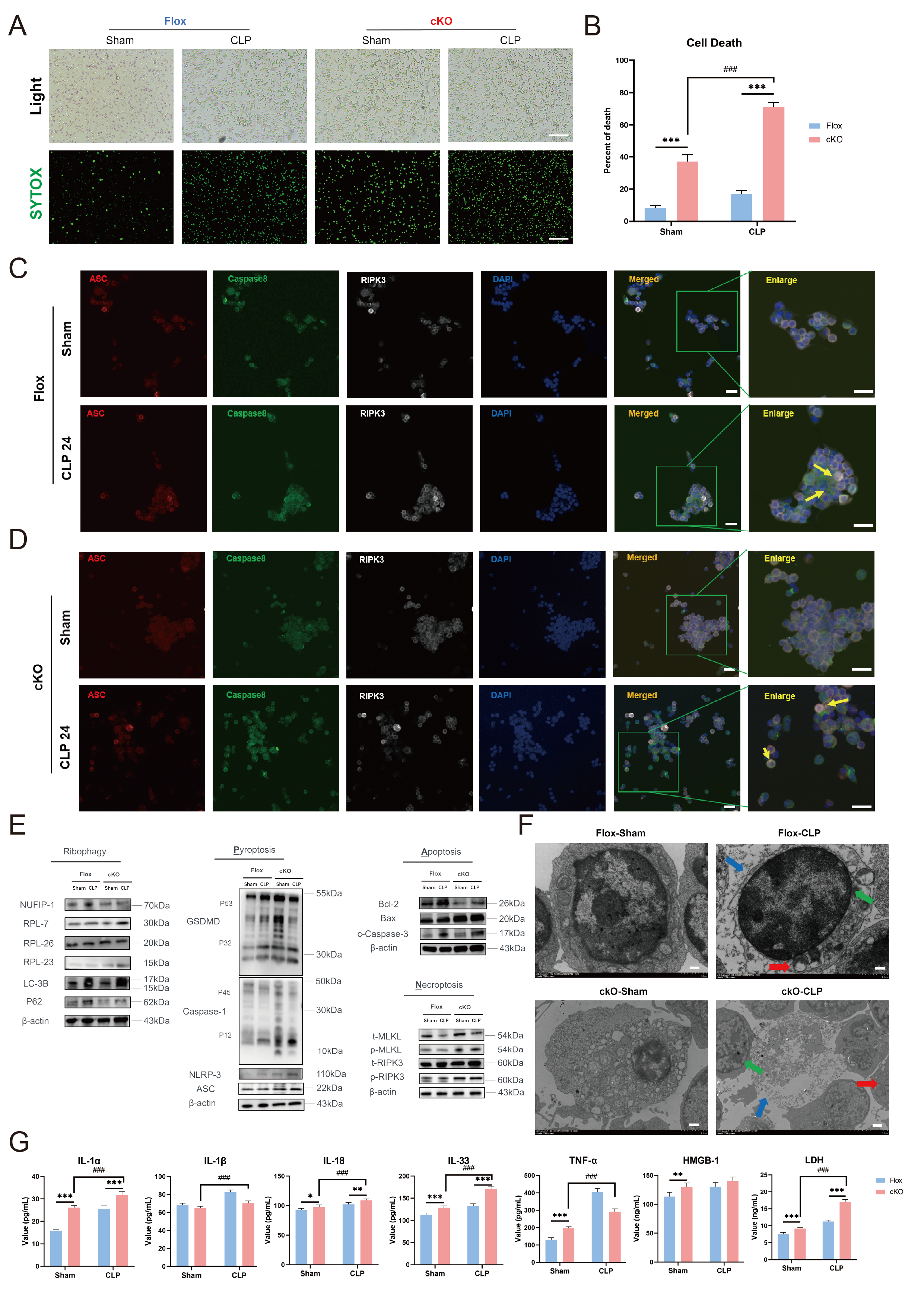

Supplement: Supplementary 1 — Supplementary Text Tables S1 to S3 Figs. S1 to S7 [file research.0895.f1.zip › Fig. S3.tif]

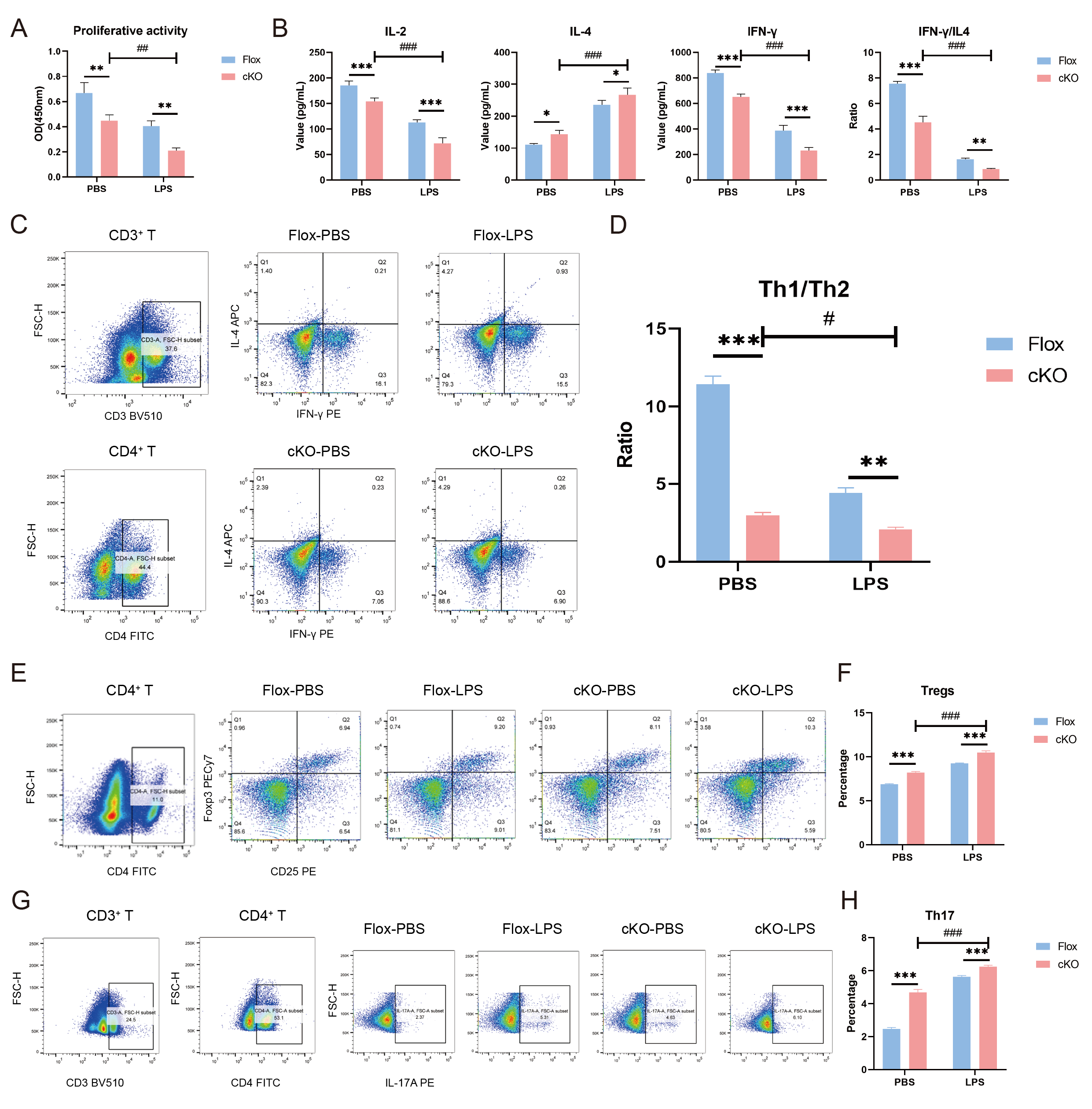

Supplement: Supplementary 1 — Supplementary Text Tables S1 to S3 Figs. S1 to S7 [file research.0895.f1.zip › Fig. S4.tif]

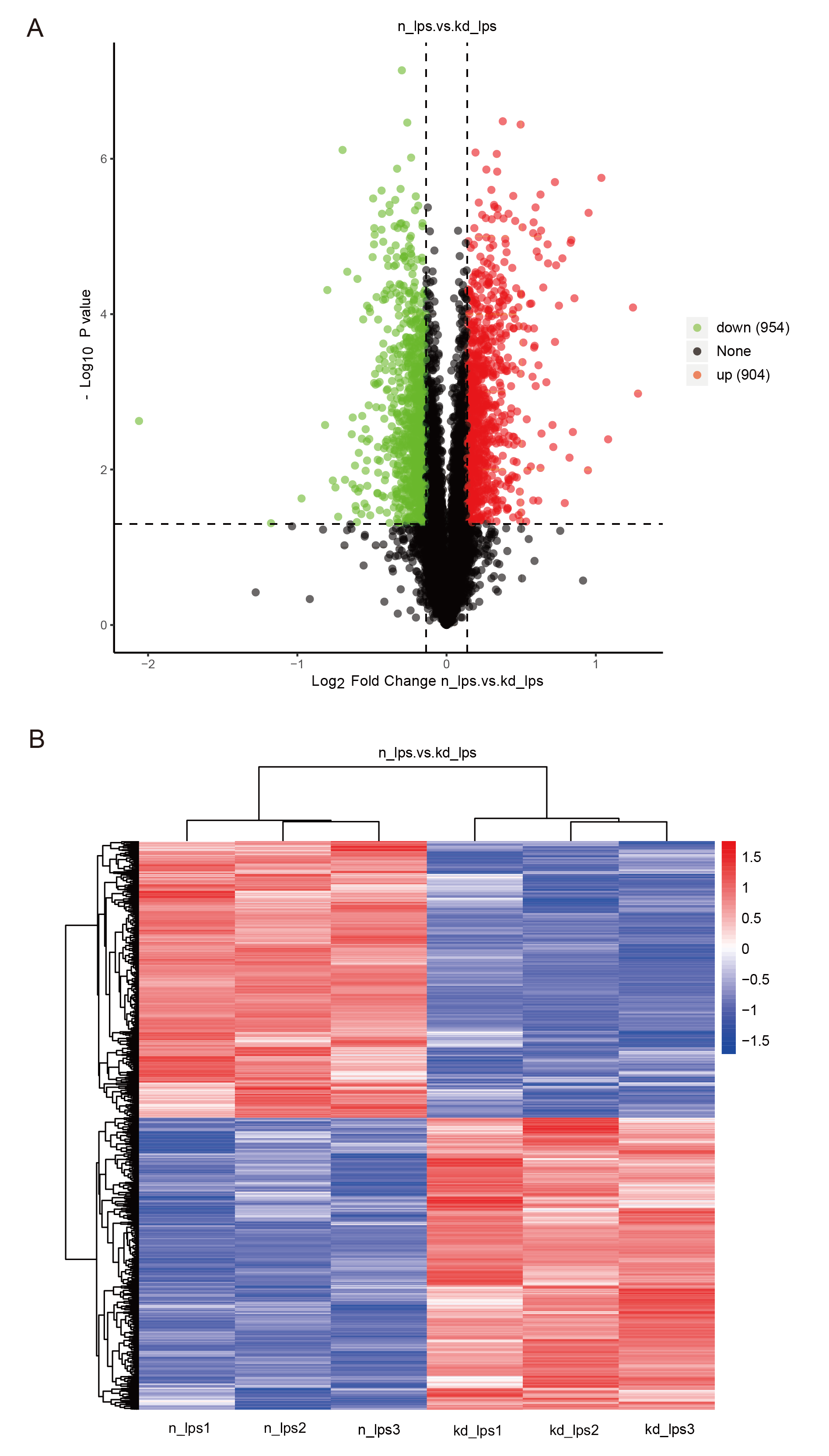

Supplement: Supplementary 1 — Supplementary Text Tables S1 to S3 Figs. S1 to S7 [file research.0895.f1.zip › Fig. S5.tif]

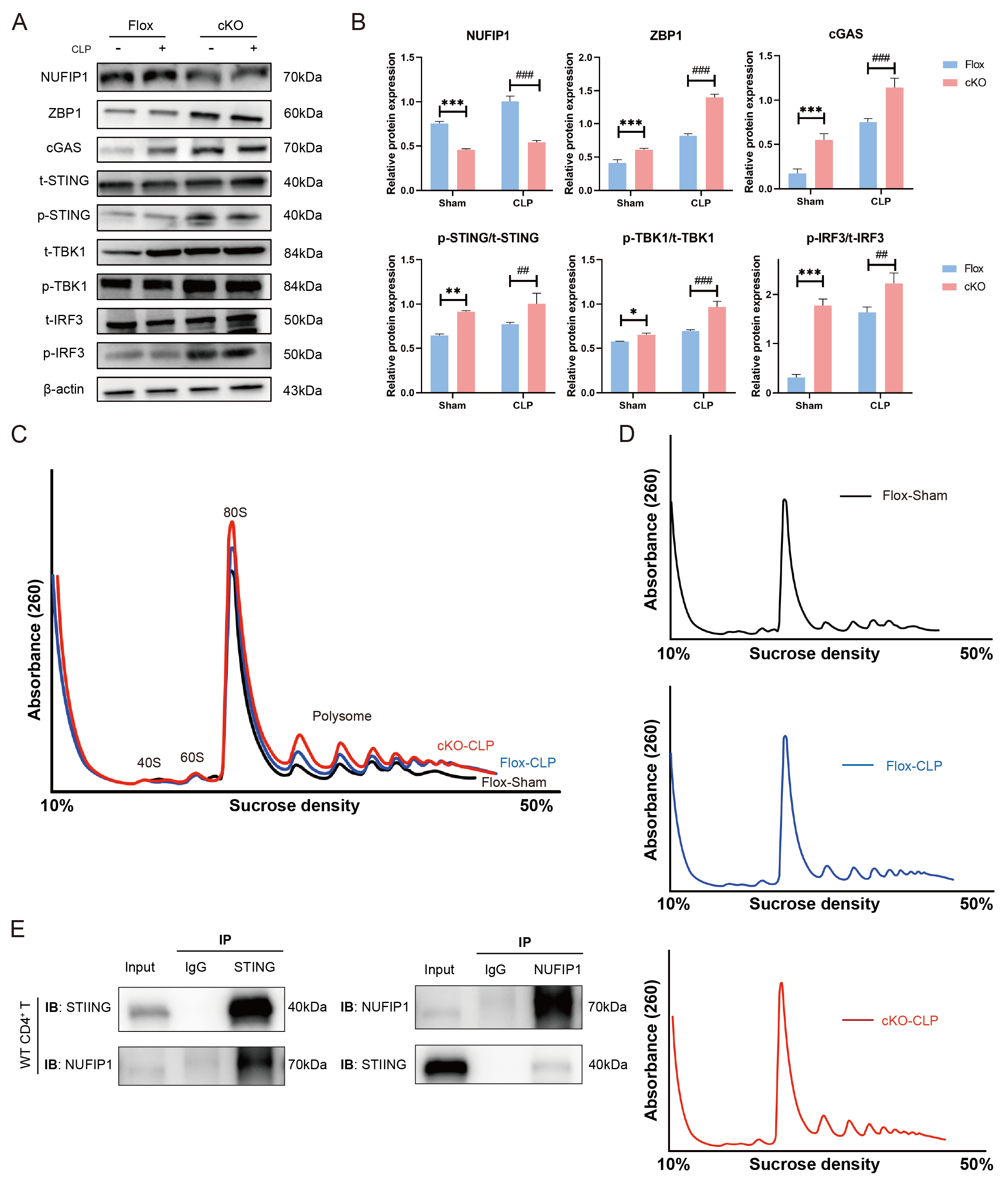

Supplement: Supplementary 1 — Supplementary Text Tables S1 to S3 Figs. S1 to S7 [file research.0895.f1.zip › Fig. S6.tif]

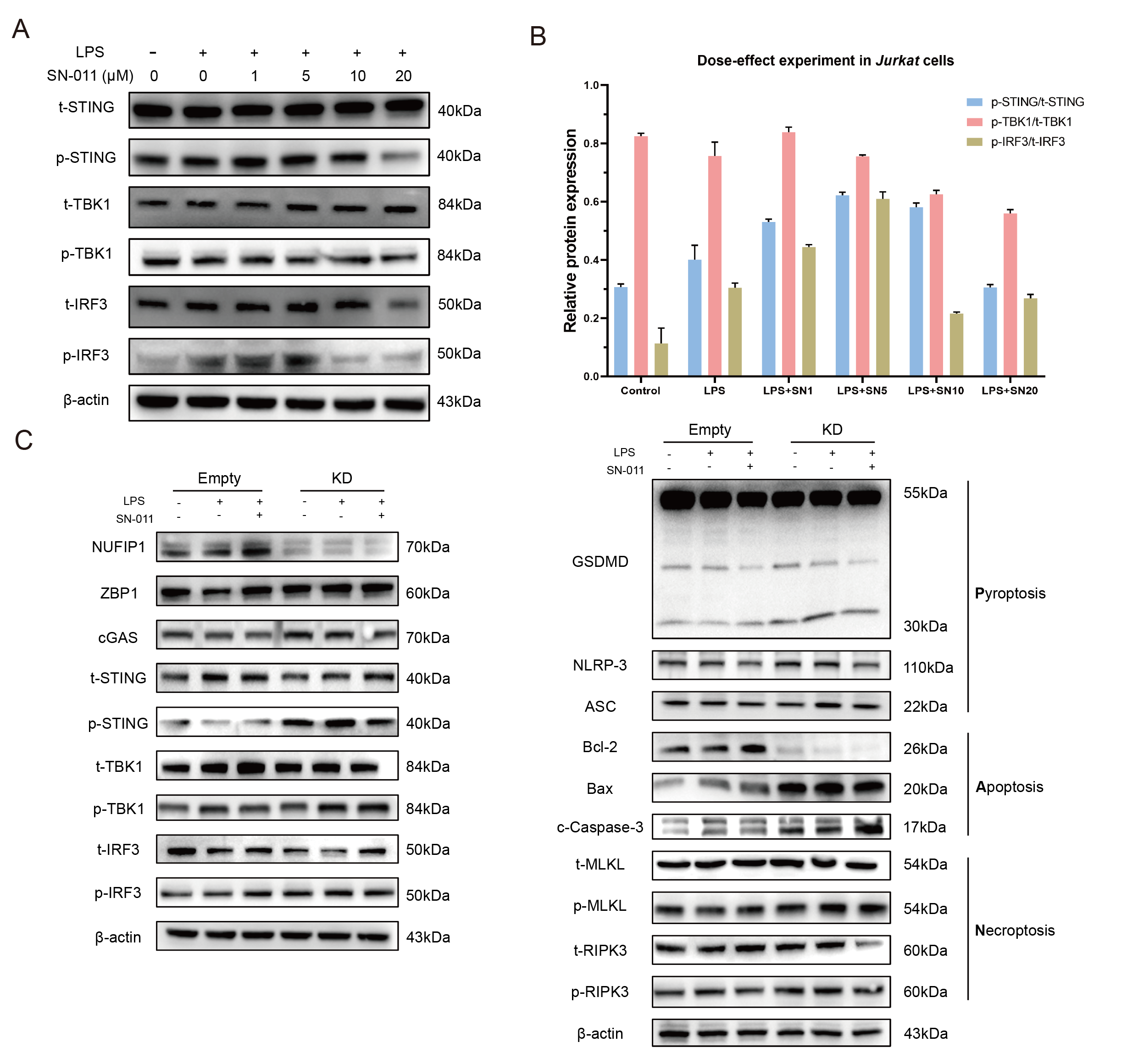

Supplement: Supplementary 1 — Supplementary Text Tables S1 to S3 Figs. S1 to S7 [file research.0895.f1.zip › Fig. S7.tif]
